# Sn‐Based Perovskite Halides for Electronic Devices

**DOI:** 10.1002/advs.202203749

**Published:** 2022-10-18

**Authors:** Towhid H. Chowdhury, Youjin Reo, Abd Rashid Bin Mohd Yusoff, Yong‐Young Noh

**Affiliations:** ^1^ Department of Chemical Engineering Pohang University of Science and Technology 77 Cheongam‐Ro, Nam‐Gu Pohang 37673 Republic of Korea

**Keywords:** field‐effect transistors, lead‐free perovskites, solar cells, tin halide perovskite

## Abstract

Because of its less toxicity and electronic structure analogous to that of lead, tin halide perovskite (THP) is currently one of the most favorable candidates as an active layer for optoelectronic and electric devices such as solar cells, photodiodes, and field‐effect transistors (FETs). Promising photovoltaics and FETs performances have been recently demonstrated because of their desirable electrical and optical properties. Nevertheless, THP's easy oxidation from Sn^2+^ to Sn^4+^, easy formation of tin vacancy, uncontrollable film morphology and crystallinity, and interface instability severely impede its widespread application. This review paper aims to provide a basic understanding of THP as a semiconductor by highlighting the physical structure, energy band structure, electrical properties, and doping mechanisms. Additionally, the key chemical instability issues of THPs are discussed, which are identified as the potential bottleneck for further device development. Based on the understanding of the THPs properties, the key recent progress of THP‐based solar cells and FETs is briefly discussed. To conclude, current challenges and perspective opportunities are highlighted.

## Graphical abstract

Tin halide perovskite (THP) is a promising material for active layer in optoelectronic and electric devices, due to its less toxicity and analogous electronic structure to that of lead. This review provides a basic understanding of the material, including lattice structure, energy band, doping and chemical instability, and presents key progress and perspectives of THP‐based high performance solar cells and transistors.

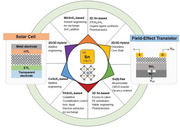

## 1 Introduction

To date, metal halide perovskite (MHP) can be considered as a promising candidate for various next‐generation optoelectronics and electronics, such as solar cells,^[^ ^1^ ^]^ field‐effect transistors (FETs),^[^ ^2^ ^]^ light‐emitting diodes,^[^ ^3^ ^]^ photodetectors,^[^ ^4^ ^]^ energy storage,^[^ ^5^ ^]^ lasers,^[^ ^6^ ^]^ memories,^[^ ^7^ ^]^ and piezoelectric applications.^[^ ^8^ ^]^ Such prompt progress of MHPs in a broad spectrum of the scientific community is due to not only its impressive optoelectronic properties but also promising cost‐effective, high‐throughput, and low‐temperature manufacturing processes.^[^ ^9^ ^]^ The bulky three‐dimensional (3D) form of MHPs comprises of a corner‐sharing BX_6_ octahedral network with the chemical stoichiometry ABX_3_, where A is an organic or inorganic monovalent cation, B is a divalent metal ion, and X is a halide anion. The B‐X‐B configuration in a BX_6_ octahedron has a bonding angle of 180°, inducing a large dispersion of both the conduction and valence bands originating from super degeneracy. This electronic structure implies that MHPs possess intrinsically outstanding and desirable electrical and optoelectronic properties such as high charge‐carrier mobility, tunable bandgaps, large optical absorption coefficient, long exciton diffusion length, and ambipolar charge transport.^[^ ^10^, ^11^, ^12^, ^13^, ^14^ ^]^ Remarkable performances from MHP‐based FETs and MHP‐based solar cells (PSCs) (power conversion efficiency (PCE) > 25%) imply the potential scopes for developing future functional optoelectronic devices.

Despite these exciting properties and advances, most MHP devices today are manufactured using Pb as the divalent metal in the ABX_3_ structure, the toxicity issue of Pb remains a long‐lasting debate. The concentration of immediately dangerous to life or health of Pb is 100 mg m^−3^, which means there is a risk of human exposure to Pb in the fabrication and handling of perovskite devices.^[^ ^15^ ^]^ Moreover, our biological systems might be in great jeopardy due to the water‐soluble properties of Pb. Thus, to prevent any perturbing issues related to Pb, the European Union has publicly announced that exposures of up to 1000 ppm are allowed for all electronic devices.^[^ ^15^ ^]^ Systematic encapsulation protocol and numerous encapsulation approaches have been given serious consideration to inhibit any possible leakage of Pb; however, it is difficult to ensure how safe it is when it comes to device fabrication as well as long‐term storage. Even with a sophisticated encapsulation procedure, there is no guarantee that potential Pb leakage can be prevented. Therefore, controlling, handling, and securing safety during the manufacturing of any related Pb‐based MHPs devices remain a huge challenge.

Apart from the encapsulation method, recent attention has been devoted to replacing Pb with low toxicity and non‐toxicity cations. Several potential low‐toxic and chemically compatible materials such as Sn,^[^ ^16^ ^]^ Bi,^[^ ^17^ ^]^ and Ge^[^ ^18^ ^]^ have been proposed to replace Pb, not only to reduce its toxicity but also to maintain the unique optoelectronic properties of perovskite. Among these materials, eco‐friendly material Sn has been widely utilized in various promising optoelectronic devices, including solar cells and FETs. Similar to that of Pb, Sn has an inactive outer shell *s* orbital, which is vital to realizing the distinctive electrical and optical properties of MHP.^[^ ^19^ ^]^ The effects of Sn substances may vary, and it is relatively safe for a human when the hydrogen bonds grow longer in Sn substances.^[^ ^20^ ^]^ Still, the influences and their adverse effect on human health have raised some eyebrows, there is a big question regarding its use.

**Scheme 1:**
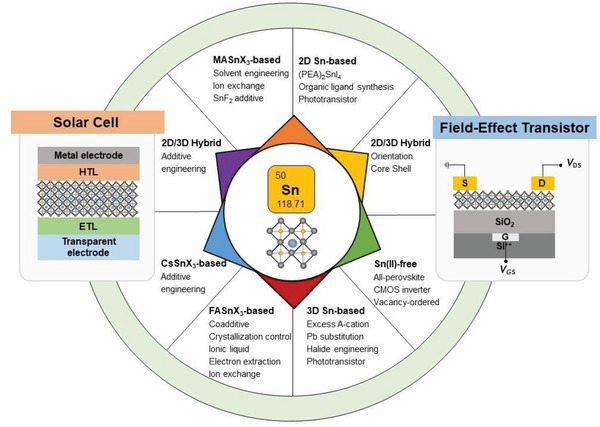
Summary of the THP‐based perovskite materials, and their specific engineering for device performance enhancement of PSCs and FETs.

The first Sn‐based perovskite (tin halide perovskite; THP) was successfully developed by Fisher et al. in 1974,^[^ ^21^ ^]^ followed by a systematic study from Donaldson's research group.^[^ ^22^, ^23^ ^]^ Later, the first hybrid THP was proposed by Yamada et al.^[^ ^24^, ^25^, ^26^ ^]^ in which Mitzi and colleagues expanded the work and conducted a study in the context of dimensional reduction, and demonstrated the first structural properties under pressure.^[^ ^27^ ^]^ These initial discoveries stimulate researchers to devote huge attention to THP‐based devices until now. In 2014, Kanatzidis et al. reported the first Sn‐based perovskite solar cells (TPSCs).^[^ ^28^ ^]^ Subsequently, numerous studies have utilized Sn instead of Pb for various type of optoelectronics devices; for instance, the latest breakthrough in TPSCs have witnessed the PCE of 14.8%, whereas the highest hole and electron mobilities of 55 and 2.1 cm^2^ V^−1^ s^−1^ for THP‐based FETs have been demonstrated.^[^ ^20^ ^]^ The rapid development in THP‐based optoelectronics devices makes it urgently necessary to review and assess their potential for several optoelectronic and electronic applications. In this regard, earlier published reviews have highlighted the progress and potential of THP's applications with solar cells,^[^ ^29^, ^30^, ^31^, ^32^ ^]^ photodetectors,^[^ ^33^ ^]^ light‐emitting devices, and radiation detectors.^[^ ^34^ ^]^ With intense research for various applications, THP‐based electronic devices show encouraging performances. However, the stability of the THP‐based electronic devices is still poor as compared to the Pb‐based ones. In this article, we aim to provide a comprehensive understanding of THP and highlight the impressive latest results for their functional device applications. First, we discuss the structure, chemistry, and thin‐film formation of THP. Second, we introduce the structure‐electrical property relationship of THP with energy band structure and electrical doping through intrinsic and extrinsic doping. Third, we review the recent progress of TPSCs. In this section, we also discuss the various chemical bonding and chemical structure of THP and their effect on the fabrication of TPSCs. Fourth, we discuss recent progress in THP‐based FETs, including two‐dimensional (2D) perovskite, hybrid 2D/3D perovskite, and 3D perovskite. Finally, we review the prospects for THP‐based PSCs and FETs, aiming to motivate the scientific community and better understand the development of future optoelectronic devices (**Scheme** **1**).

## 2 THP

### 2.1 Structure of THP

**Figure 1:**
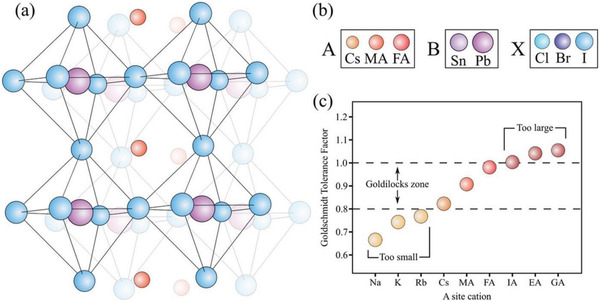
a) ABX_3_ structure of MHP, b) size of various cations and anions, and c) tolerance factor of MHPs with a variation of cations.Adapted with permission.^[^ ^43^ ^]^ Copyright 2017, American Association for the Advancement of Science.

THPs consist of ABX_3_ stoichiometry (**Figure** **1**a). To maintain the neutrality of the structure, the sum of the oxidation states of the A and B cations must be 3^+^, thus requiring a 3^−^ charge, provided by the three monovalent anions is required. A stoichiometry outside this restriction forms other dimensions of perovskite or non‐perovskite structures. The chemical and structural versatility of perovskites offers many opportunities for research and applications. The inorganic sublattice is composed of [BX_6_]^4−^ octahedra, where B is a divalent metal such as Pb^2+^, Sn^2+^, and Ge^2+^ or a mixture of monovalent and trivalent metals (e.g., Ag^+^ and In^3+^) and X is a halide: I^−^, Br^−^, or Cl^−^. By permutation of the A‐site cations and anions, a huge number of THPs are possible.^[^ ^35^ ^]^ Besides the widely used Cs and Rb, 13 protonated amines can be incorporated into the perovskite framework, as A‐site cations and eight anions, to obtain different types of perovskites. Additional structures can also be achieved through doping with various compounds. Nevertheless, to realize stable THPs, much theoretical understanding and fundamental calculation are necessary. At room temperature, the amine cations (e.g., methylammonium (MA), formamidinium (FA)) in the perovskite cavity are generally disordered, and the systems exhibit a remarkable spectrum of ferroelectric and multiferroic characteristics when cooled.^[^ ^36^ ^]^ However, to realize an ideal perovskite structure, the cations must be well‐matched with the anions. The Goldschmidt model provides an ideal method to determine the stable perovskite structure.^[^ ^37^ ^]^

(1) $$t=\frac{R_{a}+R_{x}}{\sqrt{2}\left(R_{b}+R_{x}\right)}$$

where *R_a_*, *R_b_*, and *R_x_* are the ionic radii of the A, B, and X sites, respectively. Hence, the tolerance factor (*t*) assesses whether the A‐cation can fit within the framework of corner‐sharing octahedra. Experimental data suggest that *t* values have to be in the range between 0.8 and 1.0 to maintain a 3D perovskite structure. If the *t* values are within 0.9 to 1.0, an ideal cubic phase is expected. For the largest value of *R_b_* and *R_x_* (*R*_Pb_ = 1.19 Å, *R*_I_ = 2.20 Å) within the optimum *t* limit (0.8 < *t* < 1), the ionic radius for A‐site cation to form perovskite framework was calculated at 1.60–2.60 Å.^[^ ^38^, ^39^ ^]^ Hence, MA (2.17 Å) and FA (2.53 Å) are the most suitable organic cations for developing effective perovskite compounds.^[^ ^40^, ^41^ ^]^ Incorporation of A‐cation beyond the above size limit could reduce the dimensionality of perovskite from 3D to 2D and 1D.^[^ ^42^ ^]^ Interestingly, when divalent B‐cation is replaced from large Pb to small Sn cation, the values of *t* increase and approach toward 1. One of the remarkable aspects making the crystal structures of perovskites versatile is the structural flexibility of organic groups (Figure 1b). A *t* value close to 1 can be achieved by replacing a larger monovalent A‐cation (FA^+^) with a smaller cation (Cs^+^) too. The doping of FASnI_3_ with Cs could tune the *t* downward from 1.04 to 1.02.^[^ ^43^, ^44^ ^]^ THPs have high electrical conductivity (*σ*) because of the marginal crossing of Sn 5*s* and Sn 5*p* bands near the Brillouin zone boundary (due to the large dispersion of Sn 5*s* hybridized band) point ({½, ½, ½} 2*π*/a).^[^ ^45^ ^]^ Relativistic effects (e.g., spin‐orbital coupling, effective masses of electrons and holes, orbital contraction, or inert pair effect)^[^ ^46^ ^]^ are stronger in Pb than Sn, which leads to stabilization of 6*s* ^2^ orbital of Pb relative to 5*s* ^2^ of Sn.

#### 2.1.1 MASnI_3_

**Figure 2:**
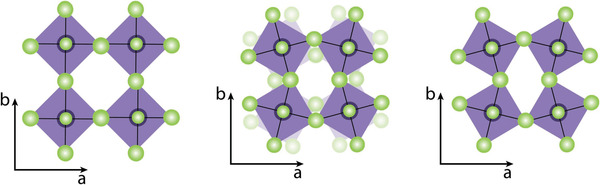
Rotation of adjacent octahedral layers along the *c*–axis according to crystal phase. a) Cubic, b) tetragonal, and c) orthorhombic phase. a–c) Reproduced under the terms of the Creative Commons CC‐BY license.^[^ ^52^ ^]^ Copyright 2022, SciELO Brazil.

MASnX_3_ perovskites were studied intensively because of their similarity with MAPbI_3_. MASnI_3_ shows a cubic *(Pm3m*) phase at 295 K; however, a phase transition from cubic to lower symmetry tetragonal (*I4/mcm*) occurs at 275 K.^[^ ^47^, ^48^ ^]^ Because the SnI_6_ octahedra tilt about the vertical axis, the tetragonal structure corresponds to a √2a × √2a × √2a supercell extension of the cubic lattice. By lowering the temperature to 108–114 K, another phase transition from tetragonal to orthorhombic (*Pbn21 or Pbnm*) can be obtained.^[^ ^49^ ^]^ In the adjacent planes, the SnI_6_ octahedra tilt in the same direction around the *c*–axis (**Figure** **2**). The A‐cation has no rotational flexibility in this arrangement. Similarly, holes become more localized, extending the lifetime of charge carriers.^[^ ^50^ ^]^ According to the first principle calculations, this phase has extremely low conductivity,^[^ ^51^ ^]^ which makes them an ideal candidate for low‐temperature electronics.

#### 2.1.2 FASnX_3_

**Figure 3:**
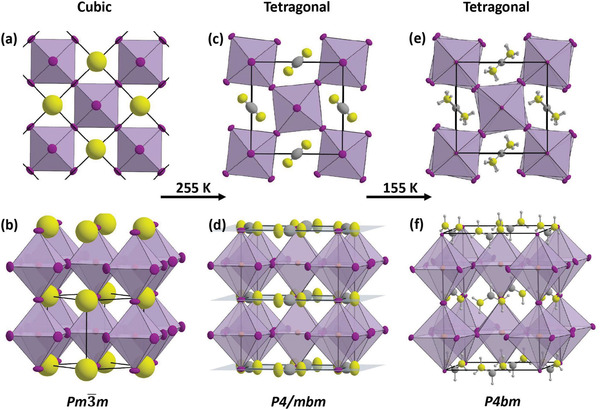
FASnI_3_ undergoes two phase transitions in the temperature range from 300 to 85 K. a,b) The cubic phase at room temperature contains fully rotationally disordered FA molecules. c,d) The structure becomes tetragonal at 255 K because of an in‐plane rotation of the SnI_6_ octahedra. The orientation of the FA molecules is two‐fold disordered due to mirror planes (shaded light blue). e,f) Doubling of the *c*‐lattice parameter occurs at 155 K, as the mirror planes perpendicular to the *c*–axis are removed and the FA molecules become fully ordered. There are now two distinct layers of SnI_6_ octahedra with different rotation angles. a–f) Reproduced with permissio.^[^ ^55^ ^]^ Copyright 2020, American Chemical Society.

At room temperature, MASnI_3_ and FASnI_3_ are isostructural. Because their symmetry does not match the O_h_ site symmetry of the cubic perovskite structure, the FA molecules are orientationally disordered.^[^ ^53^ ^]^ Because of the steric effect of the FA cation, the bond length of Sn‐I is 3.158 Å, whereas, in the MASnI_3_ system, the Sn—I bond length is 3.121 Å. FASnI_3_ forms a pseudocubic *Amm2* crystal structure at 340 K. Interestingly, a phase transition to orthorhombic phase with *Imm2* space group can be observed below 180 K for the FASnI_3_ system.^[^ ^28^ ^]^ However, clear conscientious experiments below 100 K to understand the crystal structure of FASnI_3_ have not been reported yet. Nevertheless, gradual cooling to room temperature revealed some phase transition properties for FASnI_3_ perovskites. The first phase transition upon cooling from room temperature was observed between 225 K and 250 K in cubic *Pm*3*m* to tetragonal *P4/mbm*, and a further phase transition to orthorhombic *Pnma* structure occurs between 125 and 150 K was confirmed by Schueller et al.^[^ ^54^ ^]^ Another study showed that a phase transition from a cubic room temperature to a tetragonal phase is possible at 255 K and a second tetragonal phase below 155 K.^[^ ^55^ ^]^ However, it must be considered that the variation of the crystal phases has been identified with either single crystals or polycrystalline films, which might have an impact. **Figure** **3** shows the phase transitions of FASnI_3_. At room temperature, the cubic phase contains rotationally disordered FA molecules. When the temperature is deduced to 255 K, an in‐plane rotation of the SnI_6_ octahedra occurs. A further deduction of temperature to 155 K depicts the fully ordered FA molecules. At this stage, instead of one, there are two distinct layers of SnI_6_ octahedra with different rotation angles. The inclusion of a pseudohalide SCN partially with the FASnI_3_ system can change the orthorhombic phase to monoclinic and triclinic structures.^[^ ^56^ ^]^

#### 2.1.3 CsSnX_3_

**Figure 4:**
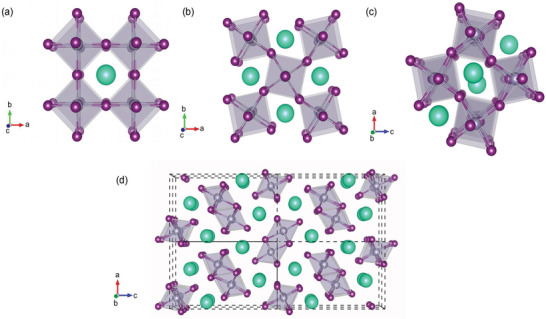
The four polymorphs of CsSnI_3_. On the top row, the three black perovskite structures are shown: a) cubic B‐*α*, b) tetragonal B‐*β*, and c) orthorhombic B‐*γ*. d) The bottom row shows the yellow crystal where the tin halide octahedral networks are fragmented into 1D chains. In all four images, the green spheres represent the Cs atom, whereas the purple polyhedra represent the octahedral perovskite cage formed by the bonding of the Sn (steel blue) and I (dark purple) atoms. a–d) Reproduced with permission.^[^ ^59^ ^]^ Copyright 2015, The American Physical Society.

When compared with MA^+^ or FA^+^, Cs^+^ is smaller in size which leads to rotation and tilt of SnI_6_ octahedral. This tilt influences the structural stability of the CsSnI_3_ at room temperature. Interestingly, at a higher temperature (500 K), the CsSnI_3_ perovskite converts to a black cubic (B‐*α*) phase (**Figure** **4**a). Upon gradual decrease of temperature to 380 K, the black cubic phase transforms into low symmetry black tetragonal phase (B‐*β*) (Figure 4b) phase. At 300 K, the phase transition to the black orthorhombic (B‐*γ*) phase is stable at room temperature.^[^ ^57^ ^]^ In this aspect, the B‐*γ* phase of CsSnI_3_ perovskite is most suitable for device applications. Nonetheless, studies suggest that exposure to ambient air or organic solvents in this phase tends to transform into a double‐chain structure as shown in Figure 4d. The formation of such a double‐chain structure is susceptible to air and often decomposes to Cs_2_SnI_6_, which has poor optoelectric and electric properties making it unsuitable for photovoltaic and FET applications. Kubicki et al. performed a detailed solid‐state nuclear magnetic resonance (ssNMR) study to compare the structures of MASnBr_3_, FASnBr_3_, CsSnBr_3_, MASnI_3_, FASnI_3_, and CsSnI_3_ perovskites.^[^ ^58^ ^]^ For every I/Br and Br/Cl mixed ratio, the ssNMR results showed that iodide–bromide and bromide–chloride mixtures can form a stable phase. Iodide–chloride mixes, conversely, produced phase‐segregated mixtures of phases, despite being somewhat miscible.

### 2.2 Band Structure and Electrical Properties

**Figure 5:**
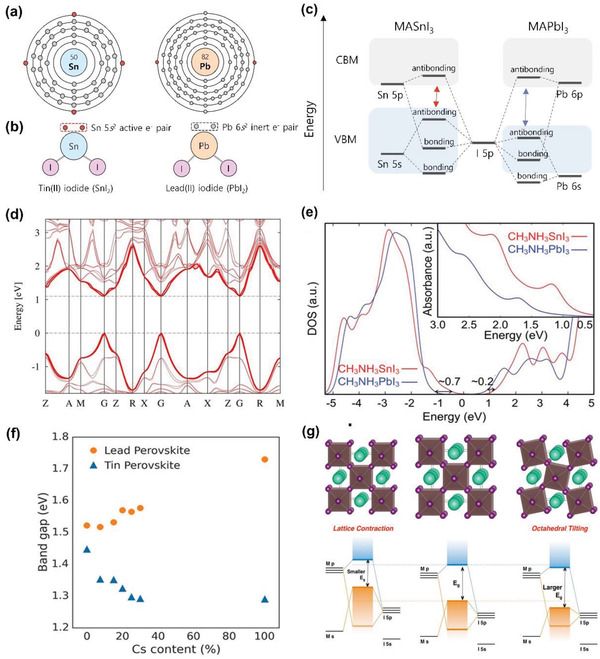
Electronic band structure of THPs. a) Electronic structure of Sn and Pb atoms. b) Schematics of *s* state valence electrons in SnI_2_ and PbI_2_. c) Energy band schematics of MASnI_3_ and MAPbI_3_. d) GW + SOC calculated band structure of MASnI_3_. e) GW + SOC calculated electronic DOSs and optical absorption spectra (inset) of MASnI_3_ and MAPbI_3_. d,e) Adapted with permission.^[^ ^48^ ^]^ Copyright 2014, Nature Publishing Group. f) Optical bandgaps of THPs and LHPs FA_1‐_ *_x_* Cs*_x_* SnI_3_ (0 ≤ *x* ≤ 1) as a function of Cs content. g) Perovskite lattice diagrams (top row) and corresponding energy level diagrams (bottom row) of lattice contraction (left panel), normal lattice (center panel), and octahedral tilting (right panel). f,g) Reproduced with permission.^[^ ^66^ ^]^ Copyright 2017, American Chemical Society.

Sn exhibits a comparable electronic configuration to Pb, as both elements possess *s* orbital lone‐pairs (**Figure** **5**a).^[^ ^48^, ^59^, ^60^, ^61^, ^62^ ^]^ Sn and Pb have similar ionic radii and optoelectronic properties, such as direct bandgaps in the visible spectrum and relatively low electron/hole effective mass.^[^ ^63^ ^]^ However, THP exhibits a different electronic band structure from that of lead halide perovskites (LHPs). The bandgap of THP is ≈1.4 eV, which is smaller than the bandgap of LHP (≈1.5 eV).^[^ ^64^ ^]^ The bandgap of THP approaches Shockley–Queisser optimal bandgap for single‐junction solar cells, and under ideal fabrication conditions, TPSCs are expected to reach a theoretical PCE up to 33.4%. Additionally, the lone‐pair state of Sn 5*s* is more reactive than Pb 6*s* because the strong lanthanide shrinkage effect of Pb makes them an inert electron pair.^[^ ^65^ ^]^ Because of the absence of this effect, Sn becomes an easily oxidized element with two active Sn 5*s* electrons (Figure 5b). This active electron pair increases the Lewis acidity of SnI_2_ over PbI_2_ and speeds up the reaction of SnI_2_ with Lewis base starting materials for perovskite such as MAI and FAI. In the following section, the formation of the energy band structure in THPs will be discussed in terms of orbital overlaps and unique properties of Sn.

#### 2.2.1 Energy Band Structure

The energy band structure is the key to analyzing the optoelectronic and electronic properties of semiconductor materials. Through combined theoretical and experimental approaches, the band structures of THP can be examined. The valence band maximum (VBM) of THP is composed of the antibonding between Sn 5*s* and I 5*p* orbitals with its main contribution from I 5*p*. Conduction band minimum (CBM) is generated from the antibonding of Sn 5*p* and I 5*p* orbitals with dominant contribution by Sn 5*p* orbital (Figure 5c).^[^ ^48^, ^60^ ^]^ Umari et al. investigated the electronic band structure of MASnI_3_ by GW + Spin‐orbital coupling (SOC) method in comparison with its Pb counterpart.^[^ ^48^ ^]^ The band structure of MASnI_3_ showed a direct bandgap of 1.1 eV (1.67 eV for MAPbI_3_ at the same point), comparable with the bandgap of ≈1.3 eV obtained experimentally (Figure 5d).^[^ ^67^, ^68^ ^]^ MASnI_3_ exhibited more robust *s*—*p* antibonding coupling near the VBM because of the shallower and more active Sn 5*s* lone‐pair states than those of Pb 6*s*. Consequently, the VBM of MASnI_3_ is more dispersive and locates ≈0.7 eV higher than that of MAPbI_3_ (Figure 5e). The calculated average hole effective mass of MASnI_3_ (0.13 m_0_) is also smaller than that of MAPbI_3_ (0.25 m_0_). In the case of CBM, the Sn 5*p* orbital is shallower and less dispersive than Pb 6*p* orbitals due to much weaker SOC, and therefore, the CBM of MASnI_3_ is less dispersive and positions at 0.2 eV higher than that of MAPbI_3_. Thus, the calculated average electron effective mass of MASnI_3_ (0.28 m_0_) is larger than that of MAPbI_3_ (0.19 m_0_).

The band structure of THP is dependent not only on B‐site cation but also on A‐site cation by modulating its molecular size. Prasanna et al. demonstrated that by reducing the ionic radius of A‐site cation, the bandgap of THP decreases in the following order: FASnI_3_ (1.41 eV), MASnI_3_ (1.30 eV), and CsSnI_3_ (1.25 eV), and a reverse trend in LHPs (Figure 5f).^[^ ^66^ ^]^ In the case of LHPs, the reduction of A‐site cation radius causes [PbI_6_]^4−^ octahedra to tilt. Consequently, the Pb‐I‐Pb angle is reduced, and the orbital overlap between Pb and I is shortened, pushing VBM to a deeper energy level and widening the bandgap. For THPs, because of the smaller size of Sn 2*p* than that of Pb 2*p*, [SnI_6_]^4−^ octahedron simply contracts instead of tilting, pushing the energy band to a shallower level, and ultimately shortens the bandgap (Figure 5g). However, the bandgap tuning with A‐site cation may only be possible in 3D perovskite structures. In 2D perovskites, the electronic state of organic cation is located deep in valence band (VB). Hence, the organic content merely participates in structural stabilization and electrostatic charge balance and does not directly contribute to electronic properties.

THPs have both advantages and disadvantages for optoelectronic applications. For example, THPs exhibit a reduced bandgap and higher absorption coefficient in the visible spectrum than LHPs. Hence, THPs are more suitable for efficient single‐junction solar cells. However, the high energy of Sn 5*s* ^2^ states easily breaks the Sn—I bonds and increases the density of Sn vacancies (*V*_Sn_). The formation of *V*_Sn_ and thus intrinsic doping in THPs will be discussed in the next section. In summary, the energy levels of Sn 5*s* and Sn 5*p* orbitals and CBM and VBM of THPs are located higher than those of Pb 6*s* orbitals and LHPs. The higher energy level of Sn 5*s* than Pb 6*s* contributes to weaker Sn—I bonding than Pb—I bonding, which increases the density of *V*_Sn_ in the thin film.^[^ ^69^ ^]^ The weak Sn—I bond also facilitates its reaction with oxygen and moisture to generate Sn—O and H—I bonds.^[^ ^70^ ^]^ Due to these subtle but critical differences in electronic band structures, THPs and LHPs exhibit different optoelectric properties and ambient stability. THPs show high hole mobility and conductivity due to smaller hole effective mass and low formation energy of *V*_Sn_, whereas LHPs show better electron transport and ambient stability as a result of their smaller electron effective mass, deep Pb 6*p* orbitals, and stronger SOC effect.^[^ ^69^, ^71^ ^]^ Hence, THPs are more suitable for *p*‐type electric devices such *p*‐channel FETs.

Electrical doping can provide additional electrons or holes into the lattice to further tune the Fermi level positions and carrier concentrations. Electrical doping within the lattice can generally be divided into two methods: intrinsic doping and extrinsic doping. Intrinsic doping is induced by self‐generated defects such as cation or anion vacancy within the constituents, whereas extrinsic doping is induced by the introduction of impurity atoms or molecules in or near the crystal lattice. Each method is discussed in the following sections.

#### 2.2.2 Intrinsic Doping

**Figure 6:**
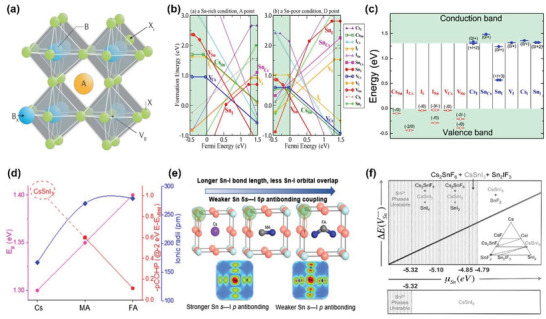
Intrinsic doping. a) Schematic of substitutional (*B_x_*), vacancy (*V*_B_), and interstitial (*X*_i_) intrinsic defects in ABX_3_ perovskite crystal lattice. Reproduced with permission.^[^ ^72^ ^]^ Copyright 2021, Nature Publishing Group. b) Calculated defect formation energy as a function of Fermi energy at Sn‐rich condition (left) and Sn‐poor condition (right). c) Calculated transition energy levels for various intrinsic defects in CsSnI_3_. b,c) Reproduced with permission.^[^ ^73^ ^]^ Copyright 2014, American Chemical Society. d) Negative associations between antibonding strength of Sn 5*s*‐I 5*p* atomic orbitals (red: measured at −2 eV below VBM via pCOHP method) and size of A‐site cations (blue) or bandgaps of corresponding Sn‐based iodide perovskites. e) Schematics of the bond length impacts on Sn 5*s*—I 5*p* antibonding coupling, also regulated by the size of A‐site cation (top), and partial charge densities around VBM level of MASnI_3_ (bottom, left) and FASnI_3_ (bottom, right). d,e) Adapted with permission.^[^ ^74^ ^]^ Copyright 2017, Royal Society of Chemistry. f) Calculated formation energy of Sn vacancy [ΔE(*V*_Sn_)] at a fixed Fermi level, as a function of Sn chemical potential (*µ*_Sn_). Reproduced with permission.^[^ ^75^ ^]^ Copyright 2014, Wiley‐VCH.

Intrinsic doping in THPs can be induced by defect formation in the ABX_3_ crystal lattice, such as vacancies (*V*_A_, *V*_B_, and *V*_X_), interstitials (*A*_i_, *B*_i_, and *X*_i_), and substitutions (*A*_B_, *B*_A_, *A*_X_, *X*_A_, *B*_X_, and *X*_B_) (**Figure** **6**a).^[^ ^72^ ^]^ Density functional theory (DFT) calculations are often used to predict the range of tuned Fermi level (*E*_F_) after defect formation and to calculate the transition level of each defect within the bandgap. For example, Xu et al. demonstrated that *V*_Sn_ is located closely below the VBM as a shallow trap.^[^ ^73^ ^]^ *V*_Sn_ is the dominant trap with low formation enthalpies, especially under Sn‐poor conditions. Hence, SnI_2_‐based perovskite films exhibit high hole density above 10^17^ cm^−3^ due to easy *V*_Sn_ formation. The *V*_Sn_ formation originates from the strong antibonding between Sn 5*s*‐I 5*p*: the stronger the antibonding is, the lower the formation energy of *V*_Sn_ becomes (Figure 6b,c).^[^ ^73^, ^74^ ^]^ The strength of Sn *s‐*I *p* antibonding was measured by crystal orbital Hamilton population (pCOHP) and showed that antibonding strength has a negative correlation with ionic radii of A‐site cation (Figure 6d). The increase in ionic radii of A‐site cation further perturbs the lattice and increases the Sn—I bond length. The optical bandgap is also affected by the extent of orbital overlap between Sn and I ions.^[^ ^60^, ^76^ ^]^ The large size A‐site cation weakens Sn *s*—I *p* antibonding and increases in energy barrier of *V*_Sn_ formation. For example, DFT calculations performed on ASnI_3_ (A = Cs, MA, and FA) demonstrated a high level of *p*‐doping under I‐rich and Sn‐poor conditions.^[^ ^73^, ^74^, ^77^ ^]^ Larger size of FA^+^ than MA^+^ resulted in a longer Sn—I bond. Consequently, Sn 5*s*—I 5*p* antibonding coupling in FASnI_3_ was weaker than that of MASnI_3_, resulting in higher *V*_Sn_ formation energy for FASnI_3_ (Figure 6e). Hence, the conductivity of FASnI_3_ can be regulated from *p*‐type to intrinsic under Sn‐rich conditions. In comparison, MASnI_3_ has high conductivity regardless of the condition. Furthermore, Ke et al. used bulky ethylenediammonium cation to modify the physical properties of MASnI_3_ and FASnI_3._^[^ ^78^, ^79^ ^]^ The high energy state and instability of Sn 5*s* ^2^ states facilitated the oxidation of Sn^2+^ to Sn^4+^. Therefore, THPs are significantly unstable in ambient air.^[^ ^80^ ^]^ The undercoordinated I^−^ ions may result in deep trap states within the bandgap because of the easy loss of Sn^2+^. Deep trap states are detrimental as non‐radiative carrier recombination centers and result in the loss of photogenerated carriers. Additionally, the deep traps degrade charge transport in FETs and provide large hysteresis in FET operation. To compare the intrinsic properties of THPs, **Table** **1** lists previously reported carrier concentrations and Hall mobilities.

**Table 1:** Intrinsic properties of THPs: carrier concentration and Hall mobility

Material	*n* [cm^−3^]	*µ*_Hall_ (p‐type) [cm^2^ V^−1^ s^−1^]	*µ*_Hall_ (n‐type) [cm^2^ V^−1^ s^−1^]	*T* [K]	Process	Measurement	Ref.
CsSnI_3_	8.73 × 10^14^	520	536	R.T.	Pressed pellet	Hall measurement	[28]
CsSnI_3_	1.00 × 10^17^	585		R.T.	Pressed powder	Hall measurement	[57]
CsSnI_3_	1.00 × 10^19^	24.5		R.T.	Thin film	Hall measurement	[90]
CsSnI_3_/BiI_3_	1.00 × 10^17^	26.9		R.T.	Thin film	Hall measurement	[90]
CsSnI_3_	3.00 × 10^16^	54		R.T.	Thin film	Hall measurement	[20]
CsSn*_x_* Pb_1‐_ *_x_* I_3_/SnF_2_	3.00 × 10^15^	486		R.T.	Thin film	Hall measurement	[20]
Cs_2_SnI_6_	1.00 × 10^14^		310	R.T.	Polycrystalline pellet	*I*–*V*/Hall measurement	[91]
Cs_2_SnI_6_	1.50 × 10^16^		79	R.T.	Thin film	Hall measurement	[92]
Cs_2_SnI_6_	6.00 × 10^16^		2.9	R.T.	Thin film	Hall measurement	[93]
Cs_2_SnI_6_	9.10 × 10^18^	20.2		R.T.	Nanocrystal solution dropcast	*I*–*V* measurement	[94]
MASnI_3_	2.00 × 10^19^	50		R.T.	Pressed pellet	Hall measurement	[83]
MASnI_3_	9.00 × 10^17^	200		250	As‐grown crystal	Hall measurement	[84]
MASnI_3_	7.94 × 10^14^	322		R.T.	Pressed pellet	Hall measurement	[28]
MASnI_3_	2.8 × 10^17^	25		R.T.	Thin film	Hall measurement	[95]
MASn(I/Br/Cl)_3_	2.2 × 10^15^	301		R.T.	Thin film	Hall measurement	[95]
(4Tm)_2_FASn_2_I_7_	5.49 × 10^18^	1.06		R.T.	Thin film	Hall measurement	[96]
FASnI_3_/(PEA)_2_SnI_4_	1.20 × 10^16^	0.21		R.T.	Thin film	Hall measurement	[97]
HC(NH_2_)_2_SnI_3_	8.38 × 10^13^		103	R.T.	Pressed pellet	Hall measurement	[28]

*n*: Carrier concentration, *µ*_Hall_: Hall mobility, T: Temperature

The accuracy of DFT calculations should be tested as it depends on several factors such as exchange–correlation functionals, the secondary‐order phase constraints, and the supercell sizes.^[^ ^81^ ^]^ Specifically, a large size supercell is necessary to omit defect–defect interaction. Although DFT calculation is an important method to determine the compositional limits of a specific material under thermodynamic equilibrium conditions, it should be verified experimentally. Intrinsic defects can be experimentally confirmed by adjusting the ratio of starting materials in various film deposition methods. The experimental results on THPs are consistent with the theoretical calculations explained above. For example, strong *p*‐doping is observed in ASnX_3_ perovskite due to the oxidation of Sn^2+^ into Sn^4+^ and easily formed *V*_Sn_ during synthesis or post‐degradation.^[^ ^47^, ^82^, ^83^, ^84^ ^]^ The density of *V*_Sn_ can be modulated in Sn‐rich conditions, such as the addition of SnF_2_ in precursor solutions. Although the excess Sn^2+^ does not incorporate into the crystal lattice, the SnF_2_ addition can increase the formation energy of *V*_Sn_ and reduce overall hole density (Figure 6f).^[^ ^75^, ^85^, ^86^, ^87^, ^88^, ^89^ ^]^

#### 2.2.3 Extrinsic Doping

**Figure 7:**
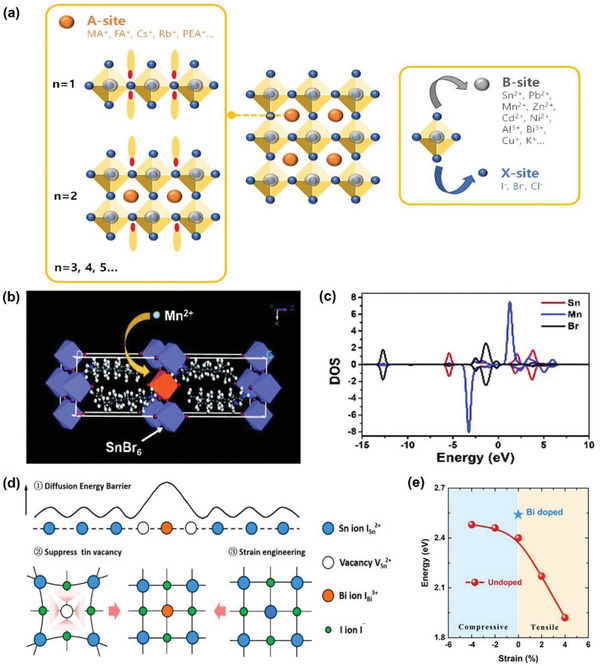
Extrinsic doping. a) Schematics of extrinsic dopants in A‐, B‐, and X‐site of ABX_3_ crystal structure. b) Schematic illustration of 2D single‐layered (C_8_H_17_NH_2_)_2_Sn_1‐_ *_x_* Mn*_x_* Br_4_ (*x* = 0.25). c) Orbital‐projected partial DOS. b,c) Reproduced with permission.^[^ ^102^ ^]^ Copyright 2020, Royal Society of Chemistry. d) Schematic representation of CsSnI_3_ crystal lattice engineering with Bi incorporation: formation of diffusion energy barrier, tin vacancy suppression, and strain engineering. e) Correlation of strain and Bi substitution on the energy barrier for Sn^2+^ diffusion. d,e) Reproduced with permission.^[^ ^90^ ^]^ Copyright 2021, American Chemical Society.

Extrinsic doping can be achieved by introducing impurity atoms or molecules in or near the perovskite lattice. **Figure** **7**a illustrates the schematic for potential extrinsic dopants in place of each atomic site in ABX_3_. The introduction of different A‐site cations in ASnX*_y_* is discussed in the previous section where the lattice contraction changes Sn—I bond length, the strength of antibonding, and the overall bandgap. The B‐site extrinsic doping in place of Sn^2+^ is especially important because the inorganic octahedral cage and its network connection theoretically have the most contribution to the electronic properties of organic–inorganic perovskites.^[^ ^60^ ^]^ Extrinsic doping of B‐site can be generally divided into two types: homovalent and heterovalent doping. Despite the significant amount of B‐site atomic doping studies for LHPs, doping studies on THPs are still premature.^[^ ^98^, ^99^, ^100^, ^101^ ^]^ To select an appropriate atomic substitutional dopant, the ionic size must be suitable to avoid host lattice distortion.

Mn^2+^ is one of the suitable candidates for homovalent doping of Sn^2+^. A previous study examined Mn^2+^ doping into octylammonium tin bromide and resulted in a red shift of PL spectrum, showing orange‐red light (Figure 7b).^[^ ^102^ ^]^ A series of measurements showed that Mn^2+^ atoms were bound to the lattice. X‐ray diffraction peaks showed a positive shift in the (001) signals, indicating Mn alloy formation with Sn.^[^ ^99^ ^]^ Additionally, according to the calculated electronic band structure and density of state (DOS), the top of VB is predominately occupied by Br orbitals and the bottom of the CB is dominated by Sn and Mn orbitals (Figure 7c). Mn orbitals are mostly located in CB enabling photogenerated electrons to jump into Mn orbitals for efficient energy transfer. Hence, the close orbital positions of Mn and Sn generate a harmonious yet competitive relationship between self‐trapped exciton emission and Mn *d*‐*d*‐transition emission. Another example candidate for homovalent doping of Sn^2+^ is Zn^2+^. Bowman et al. observed that slight addition of ZnI_2_ into the precursor solution increased carrier lifetimes, photoluminescence (PL) quantum efficiencies, and film stability in the air by reducing the formation of tin‐rich clusters, which are susceptible to oxidation.^[^ ^103^ ^]^

One of the earliest heterovalent doping studies by Takahashi et al. was based on artificially incorporating Sn^4+^ within the THP structure. The Sn^4+^ incorporation induced additional *V*_Sn_ and increased conductivity.^[^ ^104^ ^]^ Another heterovalent dopant candidate is Bi^3+^, which Zhang et al. incorporated into (PEA)_2_SnBr_4_. The Bi^3+^ incorporation resulted in a slight blue shift of the PL spectrum and reduced emission from the low energy tail.^[^ ^105^ ^]^ Zhou et al. also incorporated Bi^3+^ into the crystal lattice of CsSnI_3_. The authors demonstrated that the air instability of black phase CsSnI_3_ can be greatly lessened by Bi^3+^ dopant by restricting the direct conversion of *γ*‐CsSnI_3_ into 0D Cs_2_SnI_6_.^[^ ^90^ ^]^ The improved stability was also revealed through DFT calculations. The lattice contraction suppressed *V*_Sn_ formation and increased the energy barrier between the transformation of *γ*‐CsSnI_3_ to Cs_2_SnI_6_ (Figure 7d,e). Therefore, the introduction of an appropriate extrinsic dopant can achieve further modulate optoelectronic properties. However, heterovalent doping may not always work for all types of THPs. A combined theoretical and experimental study on the incorporation of monovalent metal cation (M^+^) into (PEA)_2_SnI_4_ revealed that the calculated formational energy of M^+^ doped‐(PEA)_2_SnI_4_ was too high and resulted in destabilized octahedral cage layer.^[^ ^106^ ^]^ Instead, experimental results showed that monovalent metal iodide existed separately within the film along the grain boundaries but was not incorporated into the lattice. Thus, monovalent metal iodides as additives enhanced the device's performance as a grain boundary passivating agent.^[^ ^106^, ^107^ ^]^ Interestingly, molecular doping, which controls the amount of charge by bringing dopant molecules closer to the perovskite lattice, is also being studied. A more in‐depth study is needed on whether this molecular doping in THP occurs through the same charge transfer as in organic semiconductors.

The effect of electrical doping of THPs is necessary to expand our knowledge of the optoelectronic and electronic properties and their tunability. Extensive theoretical and experimental studies are required to visualize the effect of intrinsic or extrinsic atomic movement on the lattice structure and the device's performance. Although doping research on LHPs is widely investigated, THPs have yet been fully explored.

## 3 Chemical Oxidation of THP

**Figure 8:**
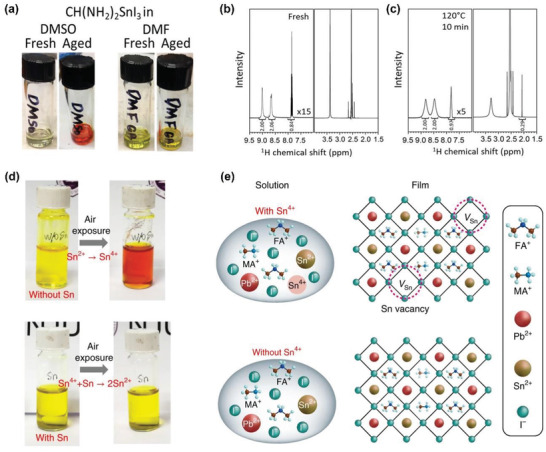
Sn oxidation in THP precursors. a) Images of fresh and aged FASnI_3_ solutions in DMSO solvent (left) and in DMF solvent (right). ^1^H NMR spectra of FASnI_3_ solutions in DMSO solvent b) before and c) after thermal treatment (120°C, 10 min). The peak at 2.07 ppm indicates the presence of DMS. a–c) Reproduced with permission.^[^ ^110^ ^]^ Copyright 2020, American Chemical Society. d) Image of Sn oxidation of Sn^2+^ to Sn^4+^ in THP solution in ambient air (top), and reduction of Sn^4+^ to Sn^2+^ with the addition of metallic Sn powder. e) Schematic representation of Sn vacancy formation due to presence of Sn^4+^ in mixed Sn‐Pb perovskite precursor solution (top) and suppression of Sn vacancy formation in Sn‐reduced precursor solution with the absence of Sn^4+^. (d,e) Reproduced with permission.^[^ ^128^ ^]^ Copyright 2019, Nature Publishing Group.

Generally, most THP films for solar cells and FETs are fabricated by spin‐coating process. To achieve good film quality, each precursor component, such as MAI, FAI, and SnX_2_ must be highly soluble in the chosen solvent. However, selecting the solvent to match the solubility of various components and to obtain high optoelectronic and electrical properties remains a challenge. For example, the solubility of Sn^2+^ compounds differs according to their halide component.^[^ ^108^ ^]^ SnCl_2_ and SnF_2_ are highly soluble in polar protic solvents such as water but are not suitable for making THP films. For the fabrication of high‐quality THP films, polar aprotic solvents are preferred because of their chemical coordination ability with all components. Because of this early research on THPs, the polar aprotic solvent *N*,*N*‐dimethylformamide (DMF) was used to fabricate THP films, despite the fact that the resulting film had poor surface morphology and limited performance when used in solar cells.^[^ ^68^ ^]^ In a follow‐up study, Hao et al. achieved better THP films by replacing DMF with dimethyl sulfoxide (DMSO) because DMSO intermediate phase controls the crystallization kinetics of the THP films.^[^ ^109^ ^]^ However, Saidaminov et al. found the irreversible redox reaction forms between dimethylsulfide (DMS) and Sn^4+^ in DMSO solution using nuclear magnetic resonance (^1^H NMR) and X‐ray absorption near‐edge spectroscopy (**Figure** **8**a–c).^[^ ^110^ ^]^ In another study reported at about the same time, Pascual et al. confirmed that Sn^4+^ tends to be prominent when a solution of FASnI_3_ in DMSO was heated at 100°C for 30 min.^[^ ^111^ ^]^ Eventually, the presence of Sn^4+^ in the precursor was identified as the main cause of defects and induces *p*‐doping in each THP film, which undesirably affects the photovoltaic performance.^[^ ^112^ ^]^ Hence, the double‐sided effects of DMSO must be carefully engineered to benefit from forming the intermediate phase with perovskite precursors and improving crystallite quality by slowing down crystallization while compensating for the accelerated Sn oxidation. In a recent study, Girolamo et al. identified 16 non‐sulfoxide solvents, 12 of which can form stable perovskite precursors at 100°C for FASnI_3_. The 12 solvents identified containing either amide, diamide, carbamate, or protic bifunctional groups were able to form SnI_2_‐solvent complexes and retard Sn oxidation to some extent. However, only mixture of *N,N*‐diethylformamide:DMPU solvent system showed some photovoltaic performances.^[^ ^113^ ^]^

The issue of Sn^2+^ oxidation into Sn^4+^ not only occurs from the chemical interaction with the solvent, but also from air exposure.^[^ ^114^, ^115^ ^]^ Upon the exposure, the highly active 5*s* ^2^ electrons of Sn^2+^ allow easy oxidation into Sn^4+^ to form *V*_Sn_ in the perovskite lattice, leaving shallow traps close to the valence band. The facile oxidation and surplus of Sn^4+^ lead to high density *p*‐type doping. This phenomenon is a critical issue for TPSCs, as oxidized perovskite layers tend to show reduced carrier lifetime and result in higher non‐radiative carrier recombination rate. Together with these dual aspects, TPSCs result in low photovoltaic performance. The easy oxidation of Sn^2+^ is also detrimental to FETs, as excess *p‐*type doping prohibits reaching an OFF‐state with extremely high hole density. Thus, to reduce the Sn^4+^ contents, several approaches have been explored, such as addition of tin halide‐derivatives (SnX_2_)_,_^[^ ^75^, ^87^, ^116^, ^117^, ^118^, ^119^, ^120^ ^]^ metallic Sn powder,^[^ ^67^, ^121^ ^]^ hydrazine,^[^ ^122^, ^123^, ^124^ ^]^ and other Lewis base reducing reagents.^[^ ^125^, ^126^, ^127^ ^]^

The fluoride in SnF_2_ increases the Sn^2+^ content of the THP films by reducing the amount of Sn^4+^ by stabilizing the oxidation caused by DMSO.^[^ ^129^ ^]^ The higher electronegativity of F or Cl can interact with the adjacent Sn^2+^ in the perovskite lattice to prevent the Sn^2+^ from losing its electron pair. Sn(CH_3_COO)_2_ has the same effect as SnF_2_ and can prevent Sn^2+^ oxidation and yield even higher photovoltaic response in TPSCs.^[^ ^130^ ^]^ Nevertheless, note that excess SnF_2_, SnCl_2_, or Sn(CH_3_COO)_2_ might induce phase segregation in THP films. Hence, an optimal amount of such additives for the fabrication of high‐quality THP films is much necessary. Lin et al. added metallic Sn powder as a reducing agent into an already oxidized, Sn^4+^ filled solution.^[^ ^128^ ^]^ The authors specifically chose Sn powder because it is insoluble in precursor solution on its own but still contributes to the perovskite lattice once oxidized by Sn^4+^ to generate Sn^2+^. Before the film fabrication, the leftover Sn residues were removed by filtering the solution. In the absence of Sn powder, the as‐prepared yellow perovskite precursor solution rapidly turned orange‐red when exposed to ambient air (Figure 8d). This color change indicates Sn^2+^ oxidation into Sn^4+^ in the solution. As previously discussed, conventional SnF_2_ additive has certain limitations in preventing Sn^2+^ oxidation, and it does not sufficiently reduce Sn^4+^ back to Sn^2+^ once oxidized. Conversely, metallic Sn readily reduces Sn^4+^ into Sn^2+^ via comproportionation reaction.

(2) $$\mathrm{Sn}+\mathrm{Sn}\left(\mathrm{IV}\right)\to 2\mathrm{Sn}\left(\mathrm{II}\right)\;\;\left(E_{0}=0.29\;V,\;\Delta G_{0}<0\right)$$

After adding metallic Sn powder into oxidized perovskite precursor solution, the orange‐red solution returned to yellow even in ambient air, indicating the successful reduction of Sn^4+^ into Sn^2+^. The addition of Sn powder can suppress the formation of *V*_Sn_ by reducing the presence of Sn^4+^, forming a Sn^4+^free precursor (Figure 8e). This method enhances the air stability of the precursor solution itself and reduces *V*_Sn_ inside the grains of the respective THP films, which has been successfully demonstrated in THP‐based FETs in a following study by Zhu et al.^[^ ^131^ ^]^

In an alternate approach, Nakamura et al. introduced a novel Sn^4+^ scavenger method by adding a tetramethyldihydropyrazine derivative (TM‐DHP) to form in situ metallic tin nanoparticles. When 1 mol% of TM‐DHP was added to the perovskite precursor solution of FA_0.75_MA_0.25_SnI_3_ in DMSO with 10 mol% of SnF_2_, the TM‐DHP reacts with the SnF_2_ rapidly and retards to Sn (0) nanoparticles. The subsequent THP film showed better film coverage with larger grains and impressive certified photovoltaic response.^[^ ^132^ ^]^ Also, Song et al. introduced hydrazine as a strong reducing reagent through vapor atmosphere, ultimately suppressing Sn^4+^ formation.^[^ ^124^ ^]^ The highly volatile hydrazine can be easily introduced and removed without thermal treatment at high temperatures. Following this report, many studies utilized hydrazine‐based materials to prohibit Sn^2+^ oxidation.^[^ ^122^, ^123^ ^]^ Alternative reducing agents with Lewis base groups, such as P—O, S—O, and C—O bonds can also form coordination interaction with Sn^2+^ in THP, inhibiting further oxidation.^[^ ^125^, ^126^ ^]^ For example, Tai et al. introduced hydroxybenzene sulfonic acid along with SnCl_2_ additive treatment as an anti‐oxidant in TPSC, showing effective control of the oxidation through aging time.^[^ ^127^ ^]^

THP devices typically used encapsulation methods for reliable operation in ambient air. The THP encapsulation methods include the coating of polymer layers, such as poly(methyl methacrylate) or Cytop, and physical methods, such as glass encapsulation.^[^ ^133^, ^134^, ^135^, ^136^ ^]^ However, these encapsulation processes can undoubtedly increase production difficulties and manufacturing costs. Based on the chemical understanding, we would like to address the following key facts that could be considered for the fabrication of THP films:

1) Solvent choice: To prevent the Sn^4+^ oxidation in the precursor solvent, a solvent system with low Sn^2+^ oxidation should be considered. This can be achieved by a single solvent or by mixing several solvents.
2) Sn^4+^ scavenger: To reduce the Sn^4+^ content in the THP films, techniques for removing Sn^4+^ from THP films should be evaluated.
3) High surface coverage: Poor film coverage occurs due to the fast film formation rate and rapid oxidation of Sn^2+^ to Sn^4+^. High film coverage can be achieved through proper solvent selection, additive addition, and control of the composition ratio of precursors.

To solve these issues, many approaches have been introduced for the development of THP‐based devices. In the following sections, we review the key development that addressed these issues for the development of TPSCs and FETs.

## 4 Application of THP for Solar Cells

### 4.1 Operation Mechanism of TPSCs

Generally, using PCE, the performance of solar cells is evaluated. The PCE of a solar cell can be obtained from the current–voltage (*J–V*) curves. Under solar light irradiation with a power intensity of *P*_in_. The *J–V* curves provide vital information on short‐circuit current density (*J*_SC_), open‐circuit voltage (*V*_OC_), and the fill factor (FF), which reveals the PCE. Briefly, PCE can be obtained by the following equation:

(3) $$\mathrm{PCE}=\frac{J_{\mathrm{SC}}\times V_{\mathrm{OC}}\times \mathrm{FF}}{P_{\mathrm{in}}}.$$

At short‐circuit conditions, the photogenerated charge carrier flows inside the solar cell, and under illumination, the total current density can be measured by incident photon flux density *J_photon_* (*λ*):

(4) $$J_{\mathrm{SC}}=q\int_{\lambda \min}^{\lambda \max}J_{\mathrm{photon}}\left(\lambda \right)\mathrm{IPCE}\left(\lambda \right)d\lambda$$

where *λ*_max_ and *λ*_min_ represent the maximum and minimum wavelength of the absorbed photons by the absorber layer, respectively, and IPCE is defined as the incident monochromatic photon‐to‐electron conversion efficiency. The IPCE of a solar cell can be described by the following equation:

(5) $$\mathrm{IPCE}\;\left(\lambda \right)=\alpha \left(\lambda \right)\eta_{c}\left(\lambda \right)\left[1-R\left(\lambda \right)\right]$$

where *α*(*λ*) is the light‐harvesting efficiency and *ηc*(*λ*) is the charge collection efficiency and *R*(*λ*) is the total reflectivity of the solar cell. From the above equations, it is eminent that all these aspects must be considered to obtain a high *J*_SC_. To obtain high light‐harvesting efficiency *α*(*λ*), the bandgap of THP film is the most critical because the photons can be absorbed only when their energy is higher than the bandgap. Additionally, for efficient photon absorption, the THP film must be thick enough with smooth surface morphology. The charge collection efficiency, *ηc*(*λ*) is determined by the carrier diffusion length and the charge‐carrier extraction capability of the adjacent charge transport layers. As mentioned in the previous sections, the THP films go through the rapid oxidation process resulting in severe *V*_Sn_, which can contribute to the lower *α*(*λ*), *ηc*(*λ*) and result in lower *J*_SC_ than the theoretical limit.

Another critical parameter for obtaining high PCE in TPSCs is the *V*_OC_, which can be described from the ideal photodiode equation:

(6) $$V_{\mathrm{OC}}=\frac{nkt}{q}\ln\frac{J_{ph}}{J_{0}}+1$$

where *J_ph_* is the reverse saturation current, which is determined by the IPCE spectrum. Therefore, to obtain high *V*_OC_, increased absorption of incident photons is necessary. To increase the photon absorption, the THP layer in the TPSC should have low bulk and surface defects. Unfortunately, the bulk and surface defects in THPs are severe due to the rapid oxidation of the Sn^2+^ states. Additionally, the *V*_OC_ of the TPSCs can be correlated to the quasi‐fermi level splitting (QFLS). Mostly, QFLS can be obtained using PL quantum yield (PLQY):

(7) $$V_{\mathrm{OC}}=\frac{\mathrm{QFLS}}{q}=\frac{1}{q}\left(E_{\mathrm{Fe}}-E_{\mathrm{Fh}}\right)$$

(8) $$\mathrm{QFLS}={\mathrm{QFLS}}_{\mathrm{rad}}+\mathrm{kTln}\left(\mathrm{PLQY}\right)$$

where *E*_Fe_ corresponds to the electron quasi‐fermi level and *E*_Fh_ corresponds to the hole quasi‐fermi level. To obtain a minimal difference between the *E*_Fe_ and *E*_Fh_, the energetic match between the THP layer and the adjacent charge transport layers is very critical. The CBM and the VBM must match well with the adjacent electron and hole charge‐carrier layers, respectively, to obtain a high *V*_OC_. The QFLS_rad_ is the radiative limit of the THP layer that is associated with the V_OC_ in zero non‐radiative recombination.^[^ ^137^ ^]^ According to Equation (8), a high PLQY can assist in obtaining high *V*_OC_. Therefore, a high PLQY of the THP layer and a suitable charge‐carrier layers with matched energy levels are a probable solution to obtaining high *V*_OC_ for TPSCs.^[^ ^138^ ^]^

The FF, as shown in equation 3 is an influential parameter to obtain a high PCE of TPSCs. The FF is mostly determined by the charge flow within the TPSC and charge transport layers. Usually, the FF correlates with the balance between the series resistance (*R*_S_) and shunt resistance (*R*_sh_) of TPSCs. To obtain a high FF, *R*_s_ should be low, and *R*_sh_ should be as large as possible to lower the power loss of the device.^[^ ^139^ ^]^ From a theoretical understanding, it is understandable that for achieving high‐performing TPSCs, it is important to consider all aspects including the THP layer fabrication with low defects and respective charge‐carrier layers with matched energy levels. Some approaches have been summarized in the following sections to obtain a high photovoltaic response.

### 4.2 Structures of TPSCs

**Figure 9:**
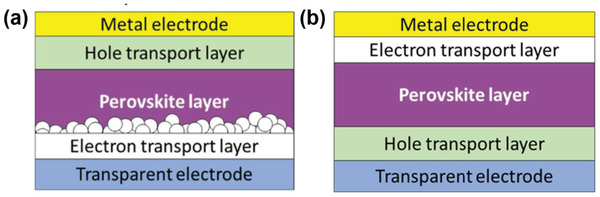
Structures of PSCs a) n‐i‐p structure and b) p‐i‐n structure.

Among the existing structures for PSCs applications, TPSCs are fabricated with mesoporous and planar (n‐i‐p) and inverted planar (p‐i‐n) designs. In an n‐i‐p structure, the THP layer is sandwiched between a compact TiO_2_ and a mesoporous TiO_2_ layer, which acts as the electron transport layer (ETL) and a hole transport layer (HTL) with a metal electrode (usually Ag and Au) (**Figure** **9**a). The compact TiO_2_ layer prevents direct contact between the two selective contacts besides being an electron transport material, whereas the mesoscopic layer serves as a basis for the nucleation and development of the THP layer, promoting charge transfer and electron collection.^[^ ^140^, ^141^ ^]^ To enable the injection of electrons, the conduction band energy level of the ETL layer must be lower than the THP layer. In this concern, apart from TiO_2_, other metal oxides such as SnO_2_ and Nb_2_O_5_ can be a suitable alternative, although they have seen limited success in TPSCs. However, the presence of oxygen vacancies at the ETL in this structure might accelerate the Sn oxidation. Conversely, the HTL layer should extract the photogenerated holes from the THP layer effectively while blocking the electrons. Generally, in Pb‐based PSCs, C_81_H_68_N_4_O_8_ (Spiro‐OMeTAD) has been widely adopted to fabricate high‐performing PSCs. However, Spiro‐OMeTAD as the HTL requires additional dopants such as lithium bis(trifluoromethanesulfonyl) imide (Li‐TFSI), 4‐tert‐Butylpyridine to extract hole efficiently from the THP layer. However, the presence of such dopants has a high influence on dissolving the THP layer. Although dopant‐free HTLs may be a probable solution for TPSC fabrication, Zhu et al. first discovered that the p‐i‐n structure is more suitable for TPSC fabrication.^[^ ^142^ ^]^ The p‐i‐n structure offers the advantage of depositing a dopant‐free ETL layer on top of the perovskite (Figure 9b). TPSC fabricated with the p‐i‐n structure exhibits less hysteresis, high PCE, and light soaking stability due to the shorter diffusion length and higher charge mobility of the THP film.^[^ ^48^, ^118^, ^143^ ^]^ In the p‐i‐n structure of TPSCs, the THP layer is fabricated on top of an HTL layer. Generally, poly(3,4‐ethylenedioxythiophene (PEDOT:PSS)^[^ ^144^ ^]^ is applied as an HTL layer with a VB level of −5.20 eV, which matches well with the VB of that THP layer.

**Figure 10:**
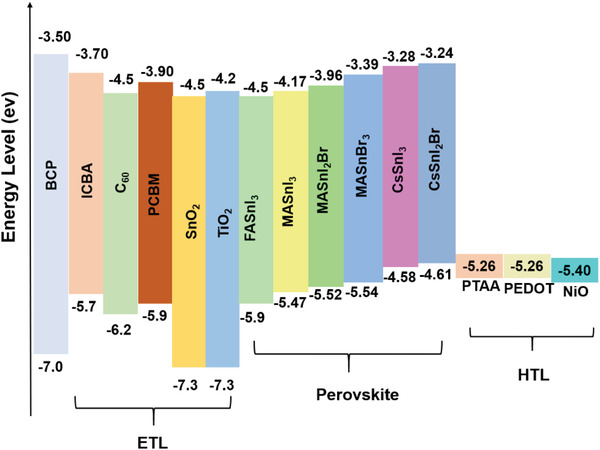
Energy level diagram of THPs with ETL and HTL.

To reduce the *V*_OC_ loss and obtain high PCE, it is important to choose suitable HTL and ETLs according to the CB and VB. Conversely, the adjacent ETL layer should have a CBM level of −4.50 eV or lower. In this aspect, [6,6]‐Phenyl C_61_ butyric acid methyl ester (PCBM) (CB = −3.90 eV)‐, C_60_ (CB = −4.50 eV)‐, and ICBA (−3.70 eV)‐based TPSCs have shown reproducible performances with impressive PCEs (**Figure** **10**). Nevertheless, note that in the respective TPSCs, the CB level of the corresponding THP was tuned by either structural modification, additive engineering, or cation displacement. Therefore, for obtaining A high PCE, a higher or lower shift of the CB or VB is expected when the THP layer is modified, and appropriate energy matching HTL or ETL is required.

### 4.3 Progress of TPSCs

#### 4.3.1 MASnX_3_‐Based Solar Cells

**Figure 11:**
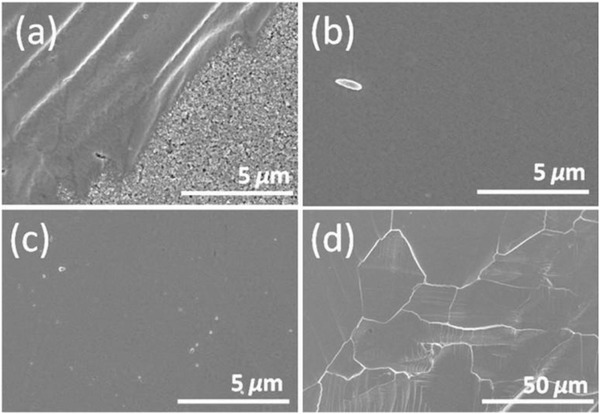
Surface morphology observed by SEM of CH_3_NH_3_SnI_3_ perovskite layer on mesoporous TiO_2_ from different solvents, a) DMF, b) NMP, and c,d) DMSO with different magnifications. a–d) Reproduced with permission.^[^ ^109^ ^]^ Copyright 2017, American Chemical Society.

The early days of TPSCs applications were conducted using MASnI_3_ as the perovskite absorber due to the structural similarity with the Pb‐based counterpart of MAPbI_3_. The energy bandgap of MASnI_3_ is 1.30 eV, which is perfect for single p–n junction solar cells. The first MASnI_3_‐based PSC was reported with a mesoporous structure and showed a PCE of 6.4%.^[^ ^68^ ^]^ The MASnI_3_ layer is deposited by the one‐step spin‐coating from a DMF precursor. The MASnI_3_ films showed high mobility and conductivity, but a substantial dark carrier concentration appeared due to severe self‐doping due to the rapid oxidation of Sn^2+^ to Sn^4+^. To reduce the dark carrier concentration and induce slower crystallization, DMSO was studied as a solvent to generate the SnI_2_·3DMSO intermediate phase.^[^ ^109^ ^]^ The DMSO‐based MASnI_3_ films exhibited a smoother surface morphology (**Figure** **11**) and high surface coverage, although their respective PSC showed significantly low PCE. Song et al. highlighted the importance of the vapor atmosphere during MASnI_3_ film fabrication and introduced hydrazine vapor to further improve the surface morphology of the spin‐coated MASnI_3_ films and reduced the Sn^4+^/Sn^2+^ ratios by 20%.^[^ ^124^ ^]^ However, the respective TPSC showed a low PCE of 3.89% in an n‐i‐p structured PSC. The low performance of the TPSCs was attributed to the lower *V*_OC_ of the devices due to the bulk defects in the THP layer. Kim et al. suggested that the large *V*_OC_ loss of MASnI_3_‐based TPSCs is due to the surface recombination at the interface rather than bulk recombination of the perovskite, which originates from the Sn^2+^ oxidation.^[^ ^145^ ^]^ A further study confirmed that the introduction of toluene instead of the diethyl ether as the antisolvent could not retard the recombination of the MASnI_3_ perovskite due to unintentional hole doping with the nonpolar solvents.^[^ ^146^ ^]^ Ke et al. proposed an alternate method by introducing ethylenediammonium (en) in the MASnI_3_ framework with a hypothesis on hollow MASnI_3_ perovskite. These {en}MASnI_3_ films showed a much lower electron‐hole recombination ratio because of the high surface coverage on top of the mesoporous TiO_2_ layer and the respective TPSCs attained a PCE of 6.63%.^[^ ^78^ ^]^ A‐cation exchange approach through a two‐step deposition process was proposed to reduce the background carrier density of the MASnI_3_ films. In the first step, a hydrazinium tin iodide (N_2_H_5_SnI_3_) layer was deposited by a simple spin‐coating technique, which was followed by a transformation into MASnI_3_ in a MA gas atmosphere through an organic cation displacement approach.^[^ ^147^ ^]^ The two‐step deposition method reduced the electron‐hole recombination of the MASnI_3_ film and resulted in dense and uniform MASnI_3_ film with micrometer‐sized grains and high crystallization and resulted in PSCs with PCE > 7%. Besides the solution‐processed mesoporous TPSCs, vapor‐assisted PSCs were evaluated by a low‐temperature vapor‐assisted solution process but showed poor photovoltaic performance.^[^ ^148^ ^]^

In an alternate attempt to reduce the bulk defect and obtain high *V*_OC_, ion exchange/insertion reactions between solid‐state SnF_2_ and gaseous methylammonium iodide was adopted for the preparation of MASnI_3_ films. Contrary to the traditional approach, the p‐i‐n was adopted where PEDOT:PSS was applied as the HTL. The respective MASnI_3_ films showed a highly uniform, pinhole‐free coverage with excess SnF_2_, and the respective film yielded a low content of Sn^4+^. This high‐quality perovskite film enables the realization of a PCE of 7.78%.^[^ ^149^ ^]^ Despite numerous efforts, the PCE of MASnI_3_‐based TPSCs is still low compared with other TPSCs. Table 1 summarizes the notable performances of MASnI_3_‐based PSCs (**Table** **2**).

**Table 2:** Development of photovoltaic performances of MASnI_3_‐based PSCs

Structure	Method	*J*_SC_ [mA cm^−2^]	*V*_OC_ [V]	FF	PCE [%]	Ref.
n‐i‐p	Solvent‐mediated crystallization by DMSO	21.40	0.32	46	3.15	[109]
n‐i‐p	Hydrazine vapor atmosphere	19.92	0.377	51	3.89	[124]
n‐i‐p	Hollow({en}MASnI_3_) perovskite as absorber	24.28	0.42	63.72	6.63	[78]
n‐i‐p	Cation exchange by HASnI_3_ with MASnI_3_	22.91	0.486	64	7.13	[147]
p‐i‐n	Ion exchange/insertion reactions between solid‐state SnF_2_ and gaseous methylammonium iodide	20.68	0.57	0.66	7.78	[149]

#### 4.3.2 FASnX_3_‐Based Solar Cells

Significant progress was achieved for TPSCs when MA cation is replaced by FA. FA cation can stabilize the crystal structure more than the MA and lead to higher photovoltaic performances.^[^ ^78^, ^79^ ^]^ Furthermore, the p‐i‐n structure is more suitable for FASnX_3_‐based TPSC fabrication. Zhu et al. compared the performance of n‐i‐p and p‐i‐n structures for FASnI_3_‐based TPSCs. Their findings highlight that the photovoltaic performance of p‐i‐n structured FASnI_3_‐based TPSCs (PCE = 7.09%) outperforms the n‐i‐p structured TPSCs (PCE = 4.34%) due to favorable energetic matching with their respective charge transport layers (ETLs and HTLs).^[^ ^142^ ^]^ Since then, almost all FASnI_3_‐based TPSCs studies have been performed utilizing the p‐i‐n structure. Hence, we only discuss here the development of FASnI_3_‐based TPSCs for p‐i‐n structure.

##### Coadditive Engineering

**Figure 12:**
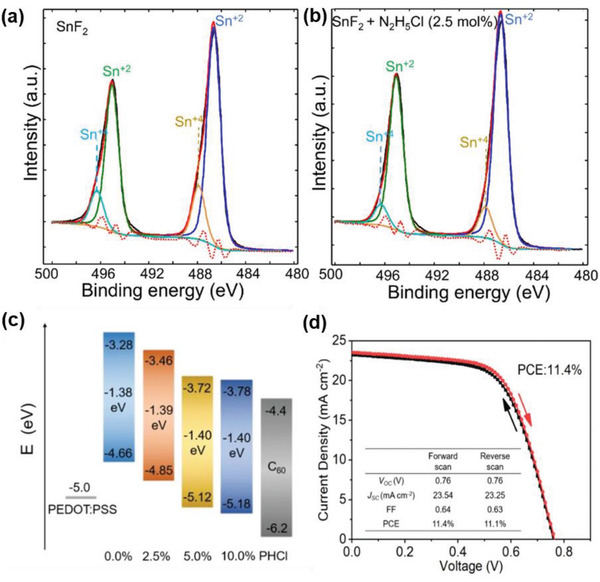
Coadditive engineering of FASnI_3_. a) High‐resolution XPS spectra (Sn 3d) of FASnI_3_ + SnF_2_ and b) FASnI_3_ + SnF_2_ + N_2_H_5_Cl (2.5 mol%) films a,b) Reproduced with permission.^[^ ^122^ ^]^ Copyright 2017, American Chemical Society. c) Potential energy diagrams (energies in eV with respect to vacuum) of PEDOT:PSS, C_60_, FASnI_3_ with 0.0%, 2.5%, 5.0%, and 10.0% of PHCl. d) The *J–V* curves of the best device with 5.0% of PHCl measured using forward and reverse scan modes under 100 mW cm^−2^ AM 1.5G irradiation c,d) Reproduced with permission.^[^ ^155^ ^]^ Copyright 2020, Wiley‐VCH.

Coadditive engineering has shown promising aspects to retard the Sn^2+^ oxidation when introducing coadditives with SnF_2_ as they tend to form a complex within the perovskite system, which can encapsulate the perovskite grains and modify the crystal structure. In the coadditive engineering approach, SnF_2_ is often used as the main additive and “other” coadditives were added. For simplicity of discussion, only “other” coadditives are discussed where mentioned. To retard the oxidization of Sn^2+^, a few reducing agents such as Catechin,^[^ ^150^ ^]^ ethylenediammonium dihypophosphite,^[^ ^151^ ^]^ and hypo‐phosphoric acid^[^ ^152^ ^]^ have been evaluated, but they failed to show a high PCE. Hydrazinium chloride showed a remarkable performance to reduce the Sn^2+^ oxidation by 20% (**Figure** **12**a,b). Besides the reduction capability of the hydrazinum, Cl^−^ contributes to achieving a pinhole‐free smooth surface morphology. The corresponding TPSCs showed 5.4% PCE and retained 65% of their initial performance after 1000 h in the N_2_ environment.^[^ ^122^ ^]^ It must be noted that by utilizing hydrazinium chloride, the shelf‐life stability for a more extended period was first observed for TPSCs. By utilizing the antioxidant properties of hydrazine, other hydrazine alternatives, such as hydrazine dihydrochloride,^[^ ^153^ ^]^ trihydrazine dihydriodide (THDH),^[^ ^154^ ^]^ and phenylhydrazine hydrochloride (PHCl) were evaluated for TPSCs. Trihydrazine dihydriodide coadditive engineered FASnI_3_ TPSCs showed improved PCE of 8.48%.^[^ ^154^ ^]^ PHCl as a coadditive showed a robust capacity to reduce the Sn^2+^ oxidation and shifted the VBM by 0.984 eV (Figure 12c), which influenced obtaining a high *J*_SC_ of 23.54 mA cm^−2^ and a remarkable *V*_OC_ of 0.76 V (Figure 12d). The presence of a hydrazino group and a hydrophobic phenyl group provided a bifunctional benefit to retard the surface defects and matched VBM with the PEDOT:PSS. The corresponding TPSCs showed PCE of 11.4% and showed stable photovoltaic performance in an N_2_ environment for 110 days.^[^ ^155^ ^]^ Interestingly, the authors noted that the incorporation of PHCl induced the self‐repairing ability of the FASnI_3_ films. Trace amount (1 mol%) of gallium acid (GA) and an excess of SnCl_2_ can lead to the formation of an amorphous GA–SnCl_2_ complex and result in the self‐encapsulated phase of the FASnI_3_ perovskite, which increases the stability of TPSCs. The large bandgap of GA‐SnCl_2_ based FASnI_3_ prohibits the transfer of both charge carriers from FASnI_3_ to the adjacent charge transport layers in TPSCs. The FASnI_3_‐based TPSCs fabricated by this complex showed a PCE of 9.03% with stable photovoltaic behavior without any degradation for 1500 h in N_2_ environment.^[^ ^156^ ^]^ Consecutively, Trimethyl thiourea (3T), with structural features of both a Lewis base and H‐bond donor can influence the fabrication of high‐quality FASnI_3_ films.^[^ ^157^ ^]^ The unique bifunctional properties of 3T as a co‐additive can successfully enhance the morphology and texture of FASnI_3_ films by spreading and joining individual crystal grains. With such attributes, the charge carrier lifetime of FASnI_3_ perovskite was increased up to 123 ns leading to low *V*_OC_ loss (only 0.2 V lower than the theoretical limit) in the respective TPSCs. Due to the high *V*_OC_ of 0.92 V, *J*_SC_ of 20.4 mA cm^−2^ and FF of 0.76, the 3T‐FASnI_3_‐based TPSCs showed a high PCE of 14.05%. It is noticeable that even with staggering *V*_OC_, the *J*_SC_ remained low, which was attributed to the low perovskite film thickness rather than the charge‐carrier diffusion length. However, even increasing the film thickness did not result in higher *J*_SC_ *_,_* rather resulted in lower *V*_OC_ *_._*

##### Controlled Crystallization

**Figure 13:**
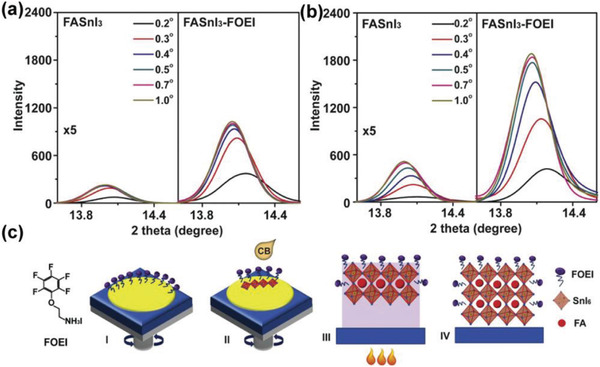
Depth‐dependent grazing incident X‐ray diffraction patterns of the FASnI_3_ and FASnI_3_‐FOEI perovskite films after antisolvent dripping a) without or b) with thermal annealing at different incident angles. The ×5 in (a) left and (b) left means that the original intensity is multiplied by 5 in this image to clearly show the tendency. c) Possible scenarios of the surface‐controlled growth of the FASnI_3_–FOEI perovskite films. a–c) Reproduced with permission.^[^ ^162^ ^]^ Copyright 2020, Elsevier.

Numerous studies on TPSCs development highlight the rapid oxidation of Sn^2+^ in FASnI_3_‐based perovskite due to the rapid crystallization of the perovskite layer. Even if the precursor solution is heat treated at 100 °C in a glovebox filled with PPM level oxygen before spin‐coating and antisolvent treatment, the organocation FA^+^ and iodide ion (I^−^) are easily volatilized and are not properly integrated into the perovskite structure. This phenomenon induces rapid oxidation and results in high crystal defects of the FASnI_3_ film. To decelerate the crystallization process, Jokar et al. highlighted ethylenediammonium diiodide (EDAI_2_) in trace amounts can lower the crystallization rate and promote the growth of highly crystalline FASnI_3_ films more slowly. During the slow crystallization process, EDAI_2_ can retard the *V*_Sn_ inside the FASnI_3_ crystal by decelerating crystal growth through a kinetic balance between nucleation and crystal growth. The 1 mol% EDAI_2_‐FASnI_3_‐based TPSCs can achieve a PCE of 8.5% and exhibit stable performance for 1400 h in an N_2_ environment.^[^ ^158^ ^]^ The *π*‐conjugated Lewis‐base molecule 2‐cyano‐3‐[5‐[4‐(diphenylamino)phenyl]‐2‐thienyl]‐propenoic acid (CDTA) shows electron‐accepting properties to reduce the oxidation and binding to the SnI_2_ at the precursor stage. This strong electron‐accepting behavior contributes to a more stable Lewis adduct during the nucleation process. The CDTA‐based FASnI_3_ films suppressed the in‐plane and out‐of‐plane rotation of [SnI_6_]^4−^ and reduced the carrier recombination of the FASnI_3_ films. Consequently, the corresponding TPSCs showed a high PCE of 10.32%, which is attributed to a 0.13 V increase in *V*_OC_ and a 10% increase in FF compared to the pristine TPSC. Controlling the crystallization via electron‐accepting small molecules of CDTA attained the first time observation of light soaking stability of the TPSCs at maximum power point tracking (MPPT) for 1000 h.^[^ ^159^ ^]^ Another molecule poly(vinyl alcohol) (PVA) with a high density of hydroxyl groups can make a strong O—H—I^−^ hydrogen bond with the FASnI_3_ system leading to nucleation sites which induce lowered crystallization rate. As a result, the FASnI_3_ perovskite crystals are more oriented and can inhibit the migration of the iodide ions to the adjacent charge transport layers. With these attributes, the *V*_OC_ value is increased from 0.55 V (for pristine TPSC) to 0.63 V (PVA based TPSC) and resulted in long‐term stable photovoltaic performance with a PCE of 8.9%.^[^ ^160^ ^]^ By taking the advantage of the slower nucleation of the H—I bond and inducing highly crystalline films, a bifunctional compound, hydroxylamine hydrochloride (HaHc) has recently been applied to reduce electronic defects of the FASnI_3_ perovskites. The hydroxyl group in the HaHc formed a hydrogen bond with iodide ion in the FASnI_3_ perovskite system to slow the crystallization, and the Cl^−^ ion coordinated with the under coordinated Sn^2+^ ions of the FASnI_3_ structure. With these bifunctional aspects of the HaHc, the corresponding HaHc‐FASnI_3_ based THP films showed lower trap assisted recombination, bimolecular recombination, and trap density as compared to the pristine FASnI_3_. With such beneficial improved electronic properties, the *V*_Sn_ was reduced and improved *V*_OC_ up to 0.676 V for the HaHc‐FASnI_3_ based TPSCs was observed with 500 h light soaking stability.^[^ ^161^ ^]^ In terms of *V*_Sn_ reduction, the H—I bond inclusion seems to be an effective approach, although the resultant TPSCs performance is still lower when compared to coadditive engineering approaches. Meng et al. proposed that the crystallization of FASnI_3_ perovskite is more vulnerable to the solution air surface. By using a tailormade fluorinated organic cation, pentafluorophen‐oxy‐ethylammonium iodide (FOEI) in the FASnI_3_ precursor solution, the surface energy of the solution/air surface was reduced, which simultaneously served as a template and resulted in highly crystalline films. When the FOEI‐FASnI_3_ films were measured using depth‐dependent grazing incident X‐ray diffraction measurements at different incident angles (**Figure** **13**a,b), the perpendicular growth at (100) plane was retarded significantly (Figure 13c). The prepared FASnI_3_‐FOEI films showed very low surface roughness and threefold increased carrier lifetime as compared to the pristine FASnI_3_ film. As a result, the FASnI_3_‐FOEI based TPSCs showed improved *V*_OC_ of 0.67 V, a *J*_SC_ of 21.59 mA cm^−2^, and a FF of 0.75, yielding a certificated PCE of 10.16%.^[^ ^162^ ^]^

Amine complex CH_3_NH_3_I·3CH_3_NH_2_ (MAI·3MA) was evaluated as potential to further stabilize the structure of the FASnI_3_ perovskite by Dai et al. When compared to the conventionalFASnI_3_:xSnF_2_ precursor, the MAI·3MA: FASnI_3_:xSnF_2_ precursor can induce chemical stability owing to due to the extra donor electron groups in the amine complex. Due to the complex formation, the crystallization rate from precursor to THP film is significantly reduced resulting in highly crystalline THP films with low Sn^2+^ oxidation and non‐radiative recombination centers. As a result, the *V*_Sn_ is reduced and the corresponding TPSC can result in *V*_OC_ of 0.65 V, leading to a PCE of 9.53% with a light soaking stability of 1000 h.^[^ ^163^ ^]^ However, in a recent breakthrough study, Jiang et al. highlighted the importance of SnI_2_ complex for regulating the crystal growth of FASnI_3_ perovskite.^[^ ^164^ ^]^ Typically SnI_2_ complexes are formed using two step synthesis process (TSS). However, SnI_2_ complexes prepared by TSS method result in SnI_2_ segregation. The segregation causes unsolvated edge‐sharing SnI_2_ clusters which limit the crystal modulation of the THP growth. To promote smooth, compact FASnI_3_ layer, and to retard the SnI_2_ segregation, a one‐step synthesis (OSS) of SnI_2_. (DMSO)*_x_* colloidal complexes were developed, where in situ, tin metal and I_2_‐DMSO in solution complex are obtained. The SnI_2_.(DMSO)*_x_* synthesized by the OSS method can be well dispersed to obtain SnI_2_ precursor without the formation of any edge‐sharing clusters. With these beneficial aspects, the stable SnI_2_. (DMSO)*_x_* colloidal complexes can assist to bind with the cations of FAI and phenylethylamonium bromide (PEABr) and promote the growth of highly crystalline and smooth THP film. As a result, the electron diffusion length of the THP film was improved to 290 ± 20 nm (versus pristine = 210 ± 20 nm). As a result, the corresponding TPSCs show a high *V*_OC_ of 0.91 V, *J*_SC_ of 20.6 mA·cm^−2^, and a FF of 0.77; leading to a PCE of 14.63%. It must be noted that by this approach TPSCs showed first time photovoltaic performance toward 15% PCE.

##### Template‐Assisted Growth

Throughout the THPs progress the solvent to film conversion rate has been highlighted as a potential source for Sn^2+^ oxidation. In recent reports, template‐assisted seed growth has shown more success to fabricate high‐quality FASnI_3_ film for TPSCs application. In a seeded growth (SG) approach proposed by Cao et al., a layer of perovskite film is first deposited by an antisolvent method, and the same step is repeated. In this method, the first spin‐coated FASnI_3_ layer acts as a template to promote the growth of uniform THP layer with low grain boundaries and highly crystalline perovskite films with compact and large grains and a longer carrier lifetime. TPSCs fabricated by the SG method showed a *V*_OC_ of 0.49 V, which is far below other conventional reported methods.^[^ ^165^ ^]^ To facilitate template assisted growth, poly(ethylene‐*co*‐vinyl acetate) (EVA) self‐sealing polymer with the ability of complex formation with SnI_2_ was tested and verified. The C=O groups of EVA act as a powerful Lewis acid–base and forms a SnI_2_ complexation with uncoordinated tin atoms in perovskite grains, which influences the improvement of the grain size, optimized grain orientation, and decrease the surface defects of FASnI_3_ films. Furthermore, The FASnI_3_.EVA based THPs show a self‐encapsulation effect which can repeal the moisture and oxygen ingression and result in humidity (60%) stable TPSCs.^[^ ^166^ ^]^

##### Treatment of FASnI_3_

**Figure 14:**
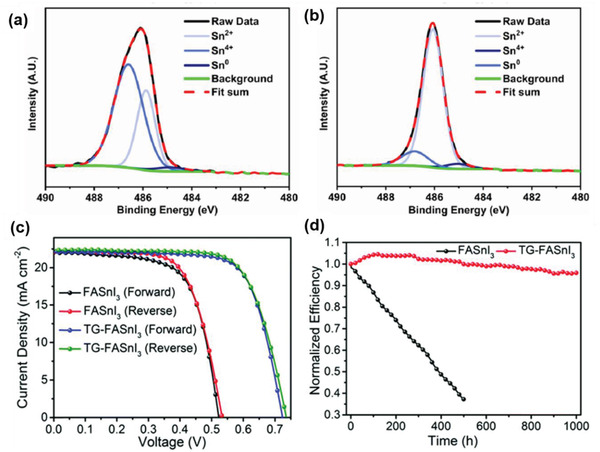
Post‐treatment–narrow XPS spectra after 10 s of argon etching of a) 0 mm edamine and b) 0.05 mm edamine‐passivated FA_0.98_EDA_0.01_SnI_3_ samples. a,b) Reproduced with permission.^[^ ^168^ ^]^ Copyright 2021, American Chemical Society. c) Pretreatment—The *J*–*V* curves of FASnI_3_ and TG‐FASnI_3_‐based TPSCs. d) The stability test of the corresponding device under simulated AM 1.5 G (100 mW cm^−2^) operating at MPPT c,d) Reproduced with permission.^[^ ^169^ ^]^ Copyright 2021, Royal Society of Chemistry.

Since the FASnI_3_ surface is more prone to Sn^2+^ oxidation and leads to extreme surface defects, the post‐treatment method was considered a possible solution. Chowdhury et al. introduced a postdeposition vapor annealing method (PDVA), where after the antisolvent treatment, the FASnI_3_ films were treated in MACl enclosed vapor system. The PDVA method induced the growth of high‐quality, defect‐free FASnI_3_ film and showed a PCE of 8.48% when implemented in TPSCs.^[^ ^167^ ^]^ A solution approach for post‐treatment by bidentate amine‐edamine was evaluated by Kamaruddin et al. Before the antisolvent treatment, edamine treatment was introduced on top of the FASnI_3_ films. The addition of edamine which has a free electron pair acts as an electron donating group and facilitates the co‐ordination with the under‐coordinated Sn^2+^ of the FASnI_3_ system (**Figure** **14**a,b). As a result, the respective THP films showed ideal crystallographic orientation near the grain boundaries and improves the surface morphology with improved grain size. With such beneficial aspects of the edamine post‐treatment, the TPSCs *J*_SC_ was improved up to 23.09 mA cm^−2^ with *V*_OC_ of 0.60 and FF of 0.73, leading to a PCE of 10.18%^[^ ^168^ ^]^ Recently, Liu et al. reported the highest certified PCE of 11.73% (by an authorized public test center) with 1000 h light soaking stability under MPPT conditions by the pretreatment method of FASnI_3_ film (Figure 14c,d) In this method, prior to the annealing of the THP film, right after the dripping of antisolvent n‐propylammonium iodide (PAI) from a mixed solvent of chloroform and DMSO was spin‐coated on top of the pre‐nucleated FASnI_3_ film.^[^ ^169^ ^]^ The PAI treatment facilitated the recrystallization of the FASnI_3_ films by forming an intermediate PAI‐FASnI_3_ phase. Although DMSO itself is known to dissolve the perovskite layer, however it was claimed that 1% v of DMSO with 100% v chloroform can successfully assist to form highly oriented FASnI_3_ crystals. With these extraordinary features, the non‐radiative recombination centers of the FAsnI_3_ film were reduced leading to high electron diffusion length. As a result, the *V*_Sn_ of the THP film was reduced and respective TPSCs showed a high *V*_OC_ of 0.73 V, leading to a PCE of 11.22%.

##### Ionic Liquid

Ionic liquids (ILs) are well known for their very low vapor pressure, making them ideal candidates for slower crystallization. When a small amount is added to the perovskite precursor, it tends to remain in the resulting perovskite film after all the solvent has been evaporated during the annealing process. With controlled crystal growth, the surface defects at the perovskite grain boundaries can be reduced effectively. Additionally, ILs can form a dipole at the perovskite/charge transport layer interface, which can improve the energy level alignment due to the reduced interfacial energy barriers.^[^ ^170^ ^]^ Lin et al. introduced 1‐butyl‐3‐methylimidazolium bromide (BMIBr) as IL to induce Ostwald ripening effect of FASnI_3_ films. During the thermal annealing of perovskite films, BMIBr IL domains generated by a lower melting point act as Ostwald ripening agents for dissolving the perovskite grains. The FASnI_3_ films fabricated by inclusion of BMIBr showed larger grain size with fewer grain boundaries which eventually reduces the defect states. The successful suppression of defect states reduces the *V*_Sn_ of the FASnI_3_ films and the corresponding TPSCs showed *V*_OC_ up to 0.68 V, with *J*_SC_ of 19.63 mA cm^−2^ and FF of 0.72 (PCE = 10.09%).^[^ ^171^ ^]^ In an alternate approach, IL formamidine acetate (FAAc) improved the FASnI_3_‐based TPSCs performance up to 9.96%. The FAAc contains CH_3_COO^−^ a group, which can form an intermediate phase and slow the nucleation of the FASnI_3_ film. Even after the thermal annealing treatment, the FAAc remained within the perovskite system and retarded the cationic vacancies of the respective THP film, helping to retain 82% of the initial PCE during the 1500 h light soaking stability test.^[^ ^172^ ^]^ *n*‐butylammonium acetate (BAAc) IL showed coordination with specific O…Sn chelating bonds and N‐H…X hydrogen bonds in the FASnI_3_ system retarded the Sn^2+^ oxidation in the precursor solution. Contrary to the other reports, the BAAc can suppress precursor Sn^2+^ oxidation even when heated at 100°C for 2 h and can form highly crystalline FASnI_3_ films with fewer surface defects. The BAAc‐FASnI_3_‐based TPSCs exhibited a PCE of 10.4% and maintained 80% of their initial photovoltaic performance for up to 100 h at 85°C.^[^ ^173^ ^]^ Note that this is the first report on TPSCs showing stable photovoltaic performance at elevated temperatures.

##### Charge Extraction Engineering

Due to the less charge extraction ability at the perovskite/ETL interface, the *V*_OC_ of the TPSCs is often low.^[^ ^174^ ^]^ Abdoul‐Sahkour et al. highlighted the importance of the charge collection loss of the FASnI_3_/ETL interface and introduced diaminomaleonitrile (DAMN) in the FASnI_3_ system to collect the photogenerated charges more effectively. The DAMN with two cyano groups which perform as an electron‐withdrawing group can successfully extract electrons effectively from the FASnI_3_ layer and transfer them to the adjacent C_60_ ETL, which was confirmed by a 42% increment of electron mobility. Consequently, the *V*_OC_ of the DAMN‐FASnI_3_‐based TPSC improved by 0.5 V compared with the pristine FASnI_3._ Due to the better charge management in the FASnI_3_ system, the DAMN‐FASnI_3_‐based TPSC showed stable photovoltaic behavior at MPPT conditions for 300 h.^[^ ^175^ ^]^ In a recent report, Chen et al. highlighted the importance of the charge transport at the HTL/FASn_0.9_Ge_0.1_I_3_ interface.^[^ ^176^ ^]^ To promote efficient charge transport at the HTL/FASnI_3,_ the FASnI_3_ perovskite was doped with 1 mol% GeI_2_. Unlike the usual dopants or additives which contribute to the surface of the perovskite layer, the GeI_2_ induces in situ formation of a thin (≈3 nm) amorphous interfacial GeO_2_ layer at the NiO*_X_*/FASn_0.9_Ge_0.1_I_3_ interface. The GeO_2_ layer prevents the Sn^2+^ oxidation at the interface, provides more robust mechanical bonding, and promotes effective charge transportation in the TPSC. As a result, the corresponding TPSCs showed a high PCE of 10.43%.

##### Cationic Displacement

**Figure 15:**
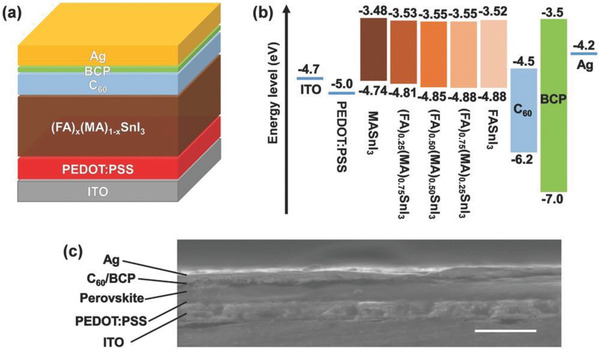
a) Schematic illustration of the device structure. b) Band alignment diagram. c) Cross‐sectional scanning electron microscope (SEM) image of a completed device (scale bar: 500 nm). a–c) Reproduced under the terms of the Creative Commons CC‐BY license.^[^ ^178^ ^]^ Copyright 2017, The Authors. Published by Wiley‐VCH.

Cationic or anionic displacement of FASnI_3_ can tune the bandgap, push the VBM to a more shallower region, increase the light‐harvesting capability of the THP films and provide a basis for high‐performance TPSC fabrication.^[^ ^177^ ^]^ Compositional engineering by cationic displacement for FASnI_3_‐based TPSCs was first evaluated by Zhao et al. where MA cation was partially introduced in the FASnI_3_ system. The increasing content of MA cation in the FASnI_3_ perovskite system shifts the VBM level and induced a favorable VBM level with the adjacent PEDOT:PSS HTL (**Figure** **15**b). The (FA)_0.75_(MA)_0.25_SnI_3_‐based TPSCs showed PCE of 8.12%, which were higher than the MASnI_3_ (PCE = 4.29%)‐ and FASnI_3_ (PCE = 6.60%)‐based TPSCs. The improved photovoltaic response was attributed to the improved *V*_OC_ by 0.15 V compared with the MASnI_3_ or FASnI_3_.^[^ ^178^ ^]^ Liu et al. discussed the importance of the appropriate antisolvent for the fabrication of FA*_X_* MA*_X_* SnI_3_ films and revealed that chlorobenzene (CB) is the more suitable antisolvent. When compared with the other antisolvents of diethyl ether and toluene, CB as an antisolvent could assist the formation of a dense, compact FA_0.75_MA_0.25_SnI_3_ layer and the respective TPSCs showed 9.06% PCE.^[^ ^179^ ^]^

In an alternate approach by vacuum‐assisted self‐healing of defects in FA_0.75_MA_0.25_SnI_3_ perovskite films to suppress the nonradiative recombination, losses were reported by Wan et al. Before the antisolvent treatment of the pre‐annealed FA_0.75_MA_0.25_SnI_3_ perovskite films were placed under high vacuum of 5 × 10^−4^ Pa. The introduction of vacuum treatment before the annealing in order to reduce the trap density reduced the nonradiative recombination and promoted interfacial charge transfer in the respective TPSCs leading to a *V*_OC_ of 0.631 V and FF of 0.75 (PCE of 10.3%).^[^ ^180^ ^]^ Inclusion of Diethylammonium iodide (DEAI) as a cation in the (DEA*_x_* FA_1–_ *_x_*)_0.98_EDA_0.01_SnI_3_ perovskite system can shift the VBM to a much deeper level (from −4.97 to −5.15 eV) and reduce the deep trap states of the respective THP film with low defect densities. With such favorable VBM level, the hole transportation between the PEDOT:PSS and the (DEA*_x_* FA_1–_ *_x_*)_0.98_EDA_0.01_SnI_3_ layer is more efficient and the *V*_OC_ loss is reduced in the respective TPSC. With an optimal 10% DEA in this mixed cation‐based TPSC a high *V*_OC_ of 0.67 V (versus pristine = 0.59 V) was attained.^[^ ^181^ ^]^ By introducing a trace amount of ethylammonium (EA) as the cation to the FASnI_3_ systems, a noteworthy improvement in TPSCs was observed by Nishimura et al. The FA_0.98_EA_0.01_SnI_3_ based THP showed a shift of 0.30 eV in VBM and 0.15 eV shift in CBM. The favorable shift of both ends facilitated obtaining a high *V*_OC_ of 0.62 V while maintaining a high *J*_SC_ of 23.05 mA cm^−2^ leading to a PCE of 10.80%. The inclusion of EA as a cation could successfully repress the trap states of the THP layer which increased charge carrier mobility. Further surface treatment of the TPSCs with EDAI elevated the *V*_OC_ up to 0.84 V and reached a PCE of 13.24%.^[^ ^182^ ^]^ However, after EDAI surface treatment, the *J*_SC_ decreased to 20.32 from 23.05 mA cm^−2^, which highlights the double‐sided effect of the surface treatment process for mixed cation THP perovskites. To overcome the drawbacks of the reduced *J*_SC_, the same research group recently introduced trimethylsilyl bromide (Me_3_SiBr) as a surface passivation agent instead of EDAI.^[^ ^183^ ^]^ Upon surface treatment with 0.15 mm Me_3_SiBr on top of the FA_0.98_EA_0.01_SnI_3_ perovskite, the surface morphology was improved, which showed lower defect densities, larger grains, and higher carrier density. As a result, the *J*_SC_ of 24.11 mA cm^−2^ can be maintained even after surface treatment. However, the *V*_OC_ of the respective TPSC was reduced to 0.70 V, leading to a PCE of 12.22%. Additionally, the Me_3_Si^+^ remains as the counter cation on the surface and forms a hydrophobic protective layer which protects the FA_0.98_EA_0.01_SnI_3_ film from oxidation which showed stable photovoltaic performance in a N_2_ glovebox for 92 days of storage in N_2_ filled glovebox by maintaining 80% of its initial efficiency.

##### Halide Exchange

By mixing the halide content of the THP, the bandgap can be tuned further. Weber et al. partially replaced the I with Br and reported that MA_0.75_FA_0.15_PEA_0.1_Sn(Br_0.33_ I_77_)_3_ can increase the bandgap from 1.29 to 1.50 eV. Although a significant improvement in TPSCs performance was expected, their fabricated TPSCs showed a low PCE of 4.63%.^[^ ^184^ ^]^ The bandgap of FASnI_3_ films was further enhanced to 1.61 eV by the addition of GuBr by Chen et al. with an optimized composition of GA_0.06_(FA_0.8_Cs_0.2_)_0.94_SnI_2_Br, which showed matched energy level alignment with the adjacent charge transport layer and reduced the trap density (both surface and bulk). The best performing TPSC showed *V_OC_* of 0.64 V, *J*_SC_ of 15.16 mA cm^−2^, and FF of 0.72 after aging.^[^ ^185^ ^]^ In a recent report, Br mixed perovskite system of EDA_0.01_(GA_0.06_(FA_0.8_Cs_0.2_)_0.94_)_0.98_SnI_2_Br, the PCE was further enhanced up to 8.66%.^[^ ^186^ ^]^ However, the improved photovoltaic performance of these wide bandgap TPSCs was attributed to the influence of replacement of PEDOT:PSS by a monolayer of 2PACZ rather than the influence of the wide bandgap THP layer (**Table** **3**).^[^ ^186^ ^]^

**Table 3:** Summary of photovoltaic performances of FASnI_3_‐based PSCs

Coadditive engineering						
Additive	Coadditive	*J*_SC_ [mA cm^−2^]	*V*_OC_ [V]	FF	PCE [%]	Ref.
SnF_2_	N_2_H_5_Cl	17.63	0.455	0.673	5.4	[122]
SnF_2_	5‐AVAI	18.89	0.592	0.623	7.0	[187]
SnCl_2_	KHSQA	17.64	0.552	0.694	6.76	[127]
SnF_2_	THDH	22.12	0.54	0.71	8.48	[154]
SnF_2_	PHCl	23.5	0.76	0.64	11.43	[155]
SnCl_2_	GA	19.75	0.64	0.714	9.03	[156]
SnF_2_	3T	20.4	0.92	0.76	14.3	[157]

Controlled crystallization						
Additive	Compound for crystallization control	*J*_SC_ [mA cm^−2^]	*V*_OC_ [V]	FF	PCE [%]	Ref.
SnF_2_	EDAI_2_	21.3	0.583	0.718	8.9	[158]
SnF_2_	CDTA	21.83	0.64	0.739	10.32	[159]
SnF_2_	PVA	20.37	0.632	0.693	8.92	[160]
SnF_2_	HaHc	19.40	0.676	0.70	9.18	[161]
SnF_2_	FOEI	21.59	0.670	0.75	10.84	[162]
SnF_2_ and NH_4_SCN	SnI_2_·(DMSO)*_x_* adduct	20.6	0.91	0.77	14.63	[164]

Template‐assisted growth						
Additive	Compound for template‐assisted growth	*J*_SC_ [mA cm^−2^]	*V*_OC_ [V]	FF	PCE [%]	Ref.
SnF_2_	EVA	22.80	0.523	0.646	7.72	[166]
SnF_2_	SG‐FASnI_3_	22.52	0.490	0.662	7.30	[165]

Ionic liquid						
Additive	Ionic liquid	*J*_SC_ [mA cm^−2^]	*V*_OC_ [V]	FF	PCE [%]	Ref.
SnF_2_	BMIBr	19.86	0.70	0.723	10.09	[171]
SnF_2_	FAAc	23.20	0.59	0.727	9.96	[172]
SnF_2_	BAAc	22.20	0.65	0.716	10.40	[173]

Cation displacement						
Additive/method	Perovskite compound	*J*_SC_ [mA cm^−2^]	*V*_OC_ [V]	FF	PCE [%]	Ref.
HAT method	(FA)_0.75_(MA)_0.25_SnI_3_	19.40	0.55	0.670	7.20	[188]
SnF_2_	(FA)_0.75_(MA)_0.25_SnI_3_	21.20	0.61	0.627	8.12	[178]
Solvent engineering	(FA)_0.75_(MA)_0.25_SnI_3_	24.30	0.55	0.673	9.06	[179]
SnF_2_ + TM‐DHP	(FA)_0.75_(MA)_0.25_SnI_3_	22.00	0.76	0.690	11.5	[132]
Vacuum‐Assisted growth	(FA)_0.75_(MA)_0.25_SnI_3_	21.62	0.63	0.755	10.3	[180]
Surface treatment with EDAI_2_	FA_0.98_EA_0.01_SnI_3_	20.32	0.84	0.78	13.24	[182]
Surface treatment with Me_3_SiBr	FA_0.98_EA_0.01_SnI_3_	24.11	0.70	0.72	12.22	[183]

#### 4.3.3 CsSnX_3_‐Based Solar Cells

Because of the absence of hydrophilic organic cations of MA and FA, CsSnX_3_ perovskites offer unique advantages in terms of structural and thermal stability. With an exciton binding energy of 12–18 meV, CsSnX_3_‐based perovskites are comparable with LHPs.^[^ ^61^, ^189^, ^190^ ^]^ However, when implemented in TPSCs, CsSnX_3_ has shown lower performances when compared with FASnI_3_‐based TPSCs. The low performance of CsSnX_3_‐based TPSCs is attributed to the *V*_Sn_, which generates severe crystal defects during the formation state of films. Distinctively, when the Sn content is increased, the *p*‐type conductivity reduces because of the reduction of accepter defects, which can influence the increase of shunt resistance and result in high‐performance TPSCs.^[^ ^73^ ^]^ To experimentally address these phenomena, Kumar et al. reported that adding 20% SnF_2_ can decrease the carrier density from 10^19^ to 10^17^ cm^−3^, which increases the formation energy of *V*_Sn_.^[^ ^75^ ^]^ Consequently, the TPSCs fabricated in n‐i‐p configuration showed a PCE of 2.02% with a *V*_OC_ of 0.24 V. An improved *V*_OC_ of 0.55 V with a PCE of 2.76% was obtained in p‐i‐n structured TPSCs when excess SnI_2_ was added to prepare the CsSnI_3_ films.^[^ ^191^ ^]^ In an alternate study, Song et al. evaluated the role of excess SnI_2_ in hydrazine vapor atmosphere and highlighted the impact on the surface morphology and crystallinity. The inclusion of excess SnI_2_ induced homogenous CsSnX_3_ film with smooth surface morphology without influencing the crystallinity. With an optimized CsI/SnI_2_ molar ratio of 0.4, a PCE of 4.8% was attained with n‐i‐p structured PSCs.^[^ ^120^ ^]^ A systematic investigation of the addition of excess SnX_2_ (X = Cl, Br, I, and F) revealed that SnCl_2_ is the most suitable compound to obtain smooth surface morphology and maintain the B‐*γ* phase of CsSnI_3_ for TPSCs applications with PCE of 3.56%. However, the partial replacement of Cl and I was not observed; rather, an ultrathin layer of SnCl_2_ layer formed on top of the perovskite layer and provided assistance to achieve stability.^[^ ^192^ ^]^

**Figure 16:**
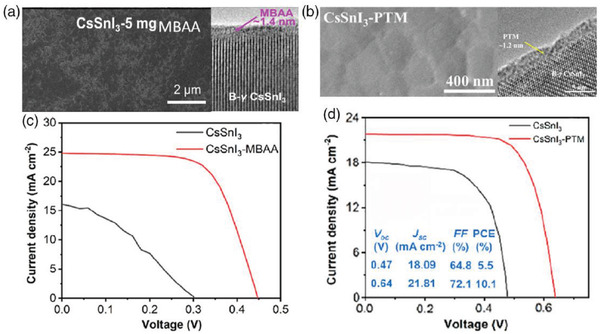
SEM and HRTEM images of a) CsSnI_3_‐MBAA and b) CsSnI_3_‐PTM. *J–V* curves of the device fabricated with c) CsSnI_3_‐MBAA and d) CsSnI_3_‐PTM. a,c) Adapted with permission.^[^ ^194^ ^]^ Copyright 2021, American Chemical Society. b,d) Adapted with permission.^[^ ^195^ ^]^ Copyright 2021, American Chemical Society.

Although the introduction of SnX_2_ additives in the CsSnI_3_ framework induced some photovoltaic response but the PCE was significantly lower than 5%. The SnX_2_ additives failed to address the critical Sn^2+^ oxidation even in an N_2_ atmosphere with a trace amount of oxygen (1 ppm). To increase the photovoltaic response, taking for example from FASnI_3_ perovskites, the coadditive strategy was adopted for CsSnI_3_ perovskite films. The addition of 2‐aminopyrazine with 20% SnF_2_ remarkably suppressed the Sn^2+^ oxidation, which was attributed to the electron‐donating capability of the amino group in the aminopyrazine and the formation of the aminopyrazine–SnF_2_ complex. Upon spin‐coating, the precursor followed by antisolvent and thermal annealing at 100°C resulted in smooth CsSnI_3_ perovskite films with compact grains. Although the *V*_Sn_ of the respective TPSC was not reduced which can be observed from the low *V*_OC_ of 0.40 V.^[^ ^193^ ^]^ A higher PCE of 7.50% was attained by utilizing the lone electron pairs of −NH and −CO functional groups of methylenebis(acrylamide) (MBAA) (**Figure** **16**a), which can coordinate with Sn^2+^ and prevent oxidation and eventually reduce the *V*_Sn_ of the B‐*γ* CsSnI_3_ films.^[^ ^194^ ^]^ Remarkably, the TPSCs showed stability for 120 h under continuous 1 sun illuminations. The lone electron pairs of –NH and two —CO units can form a trigeminal coordination bonding with Sn^2+^, which reduces the defect density of the CsSnI_3_ film. With such properties, when phthalimide (PTM) was used as a coadditive with SnF_2_, the electron density of CsSnI_3_ was successfully passivated, and smooth pinhole‐free surface morphology was observed (Figure 16b). The respective TPSCs showed the highest PCE of 10.1% for CsSnI_3_‐based TPSCs with *V*_OC_ of 0.64 V, *J*_SC_ of 21.81 mA cm^−2^, and FF of 0.721.^[^ ^195^ ^]^

In an alternate approach, B‐site engineering of the CsSnI_3_ was evaluated to tune VBM and CBM. To maintain the concept of low toxicity, Ge element with a similar electronic structure to Sn was considered and evaluated for CsSnI_3_. DFT calculations revealed that 50% of Ge inclusion in the CsSnI_3_ can induce a direct bandgap with VBM and CBM can induce a high absorption coefficient and low exciton binding energy.^[^ ^196^ ^]^ However, Ge incorporation into the precursor and thin film fabrication may not be a feasible approach for the fabrication of CsSn_0.5_Ge_0.5_I_3_‐based perovskites. Chen et al. introduced a CsSn_0.5_Ge_0.5_I_3_ perovskite powder synthesis method by solid‐state reaction in an evacuated Pyrex glass tube at 450°C.^[^ ^197^ ^]^ The as‐synthesized CsSn_0.5_Ge_0.5_I_3_ perovskite powder showed high PL intensity, a bandgap of 1.50 eV, and upshifted VBM. Interestingly, when the CsSn_0.5_Ge_0.5_I_3_ powders were evaporated to fabricate perovskite films, upon exposure to air, the formation of a Ge^4+^‐rich native‐oxide layer on the surface of the perovskite film was observed. The native‐oxide layer effectively protected the film surface, suppressed the charge recombination, and enhanced the holes extraction at the perovskite/HTL interface in an n‐i‐p‐structured TPSC. The respective TPSCs fabricated with CsSn_0.5_Ge_0.5_I_3_ achieved a PCE of 7.11% with *V*_OC_ of 0.63 V, *J*_SC_ of 18.61 mA cm^−2^, and FF of 0.606 with high ambient air stability of 500 h in an N_2_ environment.^[^ ^197^ ^]^

Apart from the typical solution processing methods, the evaporation‐assisted solution (EAS) method and the vacuum flash‐assisted solution processing (VASP) method have been evaluated for the fabrication of CsSnI_3_ films. The EAS method combines the solution process followed by vacuum evaporation of the perovskite films. In the EAS method, first, a precursor containing SnI_2_/SnF_2_ is deposited on top of the substrate by one step of spin coating and annealed at 100°C. Before annealing, CsI is thermally evaporated at 150°C for 10 min to obtain CsSnI_3_ films. The n‐i‐p‐structured TPSCs fabricated using EAS‐assisted CsSnI_3_ showed a PCE of 2.23%.^[^ ^198^ ^]^ The fabrication of CsSnI_3_ films was evaluated using the VASP method, where the CsSnI_3_ precursor solution is first deposited on top of the indium tin oxide (ITO) substrate and immediately transferred to the vacuum chamber to remove the solvent. The introduction of such a fast solvent removal technique improved the crystallization and surface properties but attained only a PCE of 3.8%.^[^ ^199^ ^]^ Yin et al. proposed a sequential vapor deposition strategy, which combined the advantages of both the vapor deposition and the surface passivation. Thiosemicarbazide (TSC) with S‐C‐N functional groups was introduced as a passivator in addition to the SnI_2_ and CsI evaporation source. The vapor deposition was performed sequentially by depositing SnI_2_, TSC, and CsI sequentially and annealing at 120°C to form a (B‐*γ*) CsSnI_3_ with large grain size and uniform coverage. The fabrication of extraordinary (B‐*γ*) CsSnI_3_ films prepared using PASVD was attributed to the strong electrostatic attachment and coordination interaction, which leads to electron cloud density and reduced the *V*_Sn_. When TPSCs were fabricated in a p‐i‐n configuration, the *V*_OC_ enhancement from 0.47 to 0.63 V was observed which is ascribed to the reduced deep level trap‐state density of the successfully passivated THP films. Interestingly, by adoption of this approach, a PCE > 8.0% was noted for CsSnI_3_‐based TPSCs with stable photovoltaic performance up to 500 h (**Table** **4**).^[^ ^200^ ^]^

**Table 4:** Development of photovoltaic performances of CsSnI_3_‐based PSCs

Structure	Method	*J*_SC_ [mA cm^−2^]	*V*_OC_ [V]	FF	PCE [%]	Ref.
n‐i‐p	SnF_2_‐MBAA	24.85	0.45	0.670	7.5	[194]
p‐i‐n	SnF_2_‐Thiosemicarbazide	19.7	0.63	0.661	8.2	[200]
p‐i‐n	SnF_2_‐Phthalimide	21.81	0.64	0.721	10.1	[195]

#### 4.3.4 2D/3D Solar Cells

2D/3D THP films exhibited a superior crystal quality when compared with 3D THP films due to the decreased background ions and carriers. To further improve the MASnI_3_ film quality, the conventional 2D/3D approach was examined. Perovskites with a Goldschmidt tolerance factor (*t*) between 0.8 and 1.0 show a photoactive black phase, which can induce high‐quality MASnI_3_ film. Ji et al. adopted EA cation into MASnI_3_ to induce a preferential orientation perpendicular to the substrate resulting in a 2D/3D MASnI_3_‐based PSC with PCE of 9.24%.^[^ ^201^ ^]^ The (EA*_X_* MA_(0.98−_ *_X_*_)_2D/3D)SnI_3_ films could successfully passivate the hole traps, induce preferential crystallization rate, produce a uniform and smooth surface, and suppress the trap‐assisted nonradiative recombination. Shao et al. introduced a lower concentration of PEAI (0.16 M) to FASnI_3_ to obtain 2D/3D structures and verified that the crystallinity of 3D THPs is significantly better when compared with the 3D FASnI_3_‐based THPs, which increased the *V*_OC_ of the respective TPSCs by 0.67 V when compared with the 3D FASnI_3_‐based TPSC and showed PCE of 9%.^[^ ^202^ ^]^ In follow‐up work, the authors evaluated the importance of stoichiometry of 2D/3D PEA_0.08_FA*_x_* SnI_3_ (where *x* refers to the concentration of the FA cation in the case of fixed PEA concentration). It was observed the concentration of FA cation significantly impacts the crystallinity of the 2D/3D films.^[^ ^203^ ^]^ A hierarchical 2D‐quasi‐2D–3D structure using NH_4_SCN as an additive was proposed to regulate the crystal growth of PEA*_x_* FA_1‐_ *_x_* SnI_3_ THP films by Wang et al.^[^ ^204^ ^]^ With regulated crystal growth, the quasi‐2D/3D THP films showed higher carrier mobility and reduced the carrier density resulting in a PCE of 9.41% in the respective TPSCs. They hypothesize that a thin layer of 2D layer of PEA_2_SnI_4_ on top of the 3D FASnI_3_ film can provide resilience against ambient air and moisture. Because of this, the respective TPSCs can maintain 90% of their initial PCE even after 600 h storage in air. Chen et al. added a bulky divalent organic cation, 4‐(aminomethyl)‐piperidinium (4‐AMP) to FASnI_3_ system. Interestingly, the addition of 15 mol% of 4‐AMP with FASnI_3_ did not influence 2D/3D formation but rather remained at the surface and grain boundaries. Interestingly, this concentration was not enough to create detectable low‐dimensional phases.^[^ ^205^ ^]^ To further elevate the PCE, Jiang et al. proposed that for the development of 2D/3D aside from the THP layer the adjacent charge carriers play a pivotal role. They replaced the ETL (PCBM) with ICBA and HTL (NiO*_X_*) with PEDOT:PSS in the PEA*_x_* FA_1‐_ *_x_* SnI_3_+NH_4_SCN‐based TPSCs and increased the *V*_OC_ up to 0.94 V, leading to a remarkable PCE of 12.4%.^[^ ^206^ ^]^ Such high *V*_OC_ was attributed to the matched energy level of the HTL/perovskite and perovskite/ETL interface.^[^ ^206^ ^]^ By using the same HTL and ETLs, the best performing TPSCs with 14.81% were reported with 4‐fluoro‐phenethylammonium cations (FPEABr) in FASnI_3_ system. It is well known that the fluorinated cations can create stronger Van der Waals interaction or hydrogen bonding reducing the Sn^2+^ oxidation and creating a 2D microstructure to influence the 3D phase growth. Additionally, fluorinated compounds provide the unique characteristics of hydrophobicity due to the presence of fluorine atoms, which resulted in the high PCE‐based TPSCs with longer stability.^[^ ^207^ ^]^ Despite the significant efforts by numerous research groups, the *V*_OC_ loss of TPSCs was minimized up to 0.45 V. Wang et al. proposed a vacuum treatment method to establish a 2D/3D stratified heterojunction which can enable charge separation at the interface. Prior to the 2D/3D stratified heterojunction formation, guanidinium thiocyanate (GuaSCN) was introduced as a co‐additive to improve the crystallinity of the 2D perovskite phase. By the synergetic effects of the vacuum treatment and GuaSCN, the respective THP films showed high hole mobility, passivated the surface traps, and prolonged carrier lifetime up to 140 ns (the highest carrier lifetime for TPSCs). The energy level diagram of the pure quasi‐2D perovskite (*n* = 2) film shows a similar valence band level *E*_V_ (−5.08 eV) to 3D FASnI_3_ film (−5.10 eV) while its conduction band level *E*_C_ (−3.26 eV) is much higher than that of FASnI_3_ (−3.71 eV), which can prohibit electron transfer across the quasi‐2D/3D mixed phase. With such beneficial aspects, the TPSCs fabricated by this method with PEA_2_FA*_n_*_− 1_Sn*_n_* I_3_ *_n_*_+ 1_(*n* = 10) showed a high *V*_OC_ of 1.01 V, which minimizes the *V*_OC_ loss of any type of TPSCs to 0.39 V (**Table** **5**).^[^ ^208^ ^]^

**Table 5:** Notable progress of photovoltaic performances of 2D/3D TPSCs

Perovskite compound	2D material	*J*_SC_ [mA cm^−2^]	*V*_OC_ [V]	FF	PCE [%]	Ref.
FASnI_3_	Evaporated PEAI	20.07	0.47	0.74	6.98	[209]
FASnI_3_	PEABr treatment	22.64	0.54	0. 64	7.86	[210]
FASnI_3_	PEAI_0.08_	24.10	0.525	0.71	9.0	[202]
FASnI_3_	PEAI‐ NH_4_SCN	22.00	0.61	0.70	9.41	[204]
FA_3_Sn_4_I_13_	BA_0.5_PEA_0.5_I	21.82	0.60	0.67	8.82	[211]
FASnI_3_	PEACl_0.12_	22.06	0.59	0.69	9.1	[212]
FASnI_3_	PEASCN_0.15_	17.40	0.94	0.75	12.4	[206]
FA*_n_*_− 1_Sn*_n_* I_3_ *_n_*_+ 1_ (*n* = 10)	PEAI_2_	20.32	1.01	0.67	13.79	[208]
FASnI_3_	FPEABr_0.10_	24.91	0.84	0.76	14.81	[207]

### 4.4 Upscaling of TPSCs

**Figure 17:**
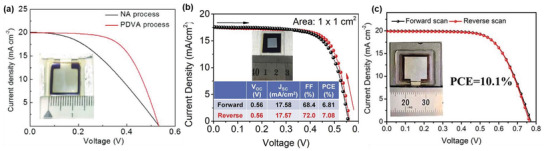
Development of 1 cm^2^‐sized TPSCs—a) PDVA processed first 1 cm^2^ TPSC. Reproduced with permission.^[^ ^167^ ^]^ Copyright 2019, Wiley‐VCH. b) Conjugated organic cation based 1 cm^2^ TPSC. Reproduced with permission.^[^ ^214^ ^]^ Copyright 2019, Elsevier. c) Two‐step deposition based on highest performance 1 cm^2^ TPSC. Reproduced with permission.^[^ ^215^ ^]^ Copyright 2022, American Chemical Society.

Despite the progress in TPSCs in terms of photovoltaic performance and stability, note that most of the performances are evaluated with a small size aperture area of 0.02–0.1 cm^2^. To realize the scalability of all types of solar cells, authorized public test centers recommend evaluating the photovoltaic behavior with an aperture area of 1 cm^2^ or more.^[^ ^213^ ^]^ However, because of the difficulty of fabricating THPs over a large aperture area, TPSCs with an aperture area greater than 1 cm^2^ have been very rarely reported. Chowdhury et al. highlighted that only a traditional approach may not be suitable for scaling of TPSCs.^[^ ^167^ ^]^ After the antisolvent treatment on the FASnI_3_ film, the PDVA process assisted by methylammonium chloride was introduced. The subsequent FASnI_3_ films fabricated by this method depicted uniform, continuous, pinhole‐free, and highly crystalline morphology, which leads to a longer PL carrier lifetime over a large aperture area. The respective TPSCs fabricated by this PDVA method showed PCE of 6.33% with *J*_SC_ = 19.59 mA cm^−2^, *V*_OC_ = 0.53 V, and FF = 0.61 with a stable performance after 200 h of storage in an N_2_ atmosphere (**Figure** **17**a).^[^ ^167^ ^]^ A conjugated organic cation containing a large volume of amines‐3‐phenyl‐2‐propen‐1‐amine (PPA) was tested for the fabrication of cm^2^‐sized TPSC by Ran et al.^[^ ^214^ ^]^ The addition of PPA to FASnI_3_ could promote the grain size, passivate the grains, reduce trap density, extract photogenerated charges, and induce preferential orientation of the perovskite film. Consequently, a PCE of 7.08% with photovoltaic parameters of *V*_OC_ of 0.56 V, *J*_SC_ of 17.57 mA cm^−2^, and FF of 0.72 (Figure 17b). Compared with the previously reported 1 cm^2^ TPSC based on the PDVA process, the conjugated PPA introduction within the FASnI_3_ framework showed a higher FF due to the better charge management of the TPSC.^[^ ^214^ ^]^ In a recent report, instead of adopting the one‐step antisolvent method, a two‐step method was evaluated as a potential route to solve the scaling issue of TPSCs. In this method, the SnI_2_ layer with a 10% SnF_2_ in DMSO was first deposited by a spin‐coating method followed by the deposition of FAI in a 2‐methyl‐2‐butanol solvent. The implementation of two‐step deposition successfully induced coverage over a large surface area with lower defects and showed PCE of 10.09% with *J*_SC_ of 19.96 mA cm^−2^, *V*_OC_ of 0.77 V, and FF values and 0.667% (Figure 17c).^[^ ^215^ ^]^ Compared with the previously reported 1 cm^2^‐sized PSCs, the TPSCs fabricated by the two‐step method showed a higher *V*_OC_ of ≈0.21 V, which was attributed to the steric hindrances of 2‐methyl‐2‐butanol to hydroxyl introduced at the second step. By lowering the crystallization process, a uniform surface morphology over a large surface area was achieved which increased the PL lifetime and influenced the photovoltaic performance.

### 4.5 Challenges and Future Perspective

Recent reports show various approaches to overcome some key bottlenecks of the TPSCs development. Nevertheless, obtaining all aspects of an efficient TPSC such as *J*_SC_, *V*_OC_, and FF remains a challenge. The literature review presented above shows that when a high *V*_OC_ is achieved, the *J*_SC_ remains low and when a favorable *J*_SC_ is achieved the *V*_OC_ remains low. With such characteristics, the PCE of TPSCs is now in the range of ≈14%, which is significantly lower than the 33% of the theoretical limit. Although numerous researches have shown promising aspects to creating a balance between the photovoltaic parameters by the approaches mentioned above, the real intrinsic mechanism to achieve high photovoltaic performances remains unknown. As can be observed, there is a significant lack of understanding of various THP material properties. Especially for high‐performing FASnI_3_ materials, many physical and electrical properties remain unknown. Therefore, apart from TPSC development, future research should be focused on understanding the THP material's optoelectronic properties, chemical behavior, and film properties. Since the complexity of TPSCs fabrication by solution‐processable methods was also highlighted, other alternative fabrication methods must be evaluated.

## 5 Application of THP for FETs

### 5.1 Operation Mechanism of FETs

**Figure 18:**
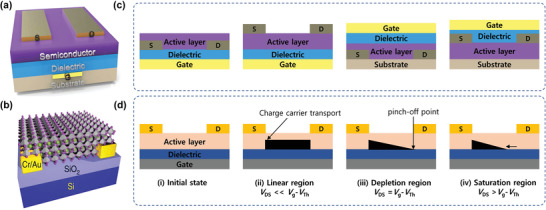
Structure and operational mechanism of FETs. a) Cross‐sectional schematic of a typical FET. Reproduced with permission.^[^ ^216^ ^]^ Copyright 2016, Wiley‐VCH. b) FET structure of hybrid perovskite FET. Reproduced with permission.^[^ ^217^ ^]^ Copyright 2016, Nature Publishing Group. c) Schematic illustrations of the four types of common FET configurations: (from left) bottom‐gate bottom‐contact (BGBC), bottom‐gate top‐contact (BGTC), top‐gate bottom‐contact (TGBC), and top‐gate top‐contact (TGTC). d) Schematic representations of FET operating regions: i) initial state, ii) linear region, iii) depletion region, and iv) saturation region.

FETs are the fundamental units in microelectronics for amplifying and switching electronic signals through modulating the electric field.^[^ ^128^, ^218^ ^]^ A typical FET is constructed with four main components: gate, dielectric, active layer, and electrodes (**Figure** **18**a). The dielectric layer separates the gate electrode from the active semiconducting layer. The most commonly used dielectric in FETs is silicon dioxide (SiO_2_).^[^ ^219^, ^220^ ^]^ However, it shows a high driving voltage because of the low dielectric constant, when the thickness is reduced, the charge carriers penetrate thin layers of SiO_2_, resulting in a high gate leakage current, which is a critical fault of device downscaling.^[^ ^221^ ^]^ Hence, materials with high dielectric constant may replace SiO_2_ in the future for the downscaling of FETs. The active layer is where charge‐carrier transport takes place and determines the field‐effect conductivity of the devices. For the n‐type active layer, amorphous metal oxides, especially indium gallium zinc oxide (IGZO), have succeeded in commercialization for backplane TFT of the large‐area organic light‐emitting display.^[^ ^222^, ^223^ ^]^ For the p‐type active layer, various materials such as silicon (Si) and other inorganic III‐V semiconductors have been developed until now but suffer from the high cost and complexity of fabrication.^[^ ^224^, ^225^ ^]^ Although the search for alternative semiconductors beyond inorganics has taken its place in organic materials with low cost, convenience, and scalability of fabrication, its low carrier transport mobility still limits the performance.^[^ ^226^, ^227^ ^]^ Recently, MHP stood out as a promising semiconductor candidate for FET active layer due to suitable features, such as high charge‐carrier transport mobility and solution processability (Figure 18b).^[^ ^228^, ^229^, ^230^, ^231^ ^]^ The charge‐carrier transport begins when a bias voltage is applied to the gate and metal contacts, which are also called electrodes. Metals that have suitable work functions when matched with the active layer can be used as source and drain electrodes. The distance between two electrodes is called the channel length *L* and the width is called channel width *W*.

As illustrated in Figure 18c, there are typically four types of transistor configurations depending on the positions of contact electrodes and gate: bottom‐contact bottom‐gate (BCBG), bottom‐contact top‐gate, top‐contact top‐gate (TCTG), and top‐contact bottom‐gate.^[^ ^232^, ^233^ ^]^ The FET performance may vary significantly based on its fabricated configuration due to differences in interface contacts, particularly one between the active layer and the contacts, and the other between the active layer and the dielectric layer. Bottom‐gate devices can achieve a good interface between the active layer and the dielectric layer and high‐quality surface. However, the active layer is directly exposed to the environment and susceptible to degradation. In comparison, top‐gate devices can reduce potential degradation from exposure; however, the active layer may be influenced by the deposition of dielectric or contact layers.^[^ ^234^ ^]^

In a typical FET, a bias voltage is applied to the gate and drain electrodes, whereas the source electrode is grounded. Gate voltage (*V*_g_) creates a potential difference between gate and source, which is a driving electric force for charge carriers to accumulate at the channel between source and gate electrode in enhancement mode devices. Drain voltage generates a potential difference between source and drain that induces the accumulated charge carriers at the channel to transport from source to drain. Gate and drain voltage are applied simultaneously and form a controllable current between source and drain. Thus, the magnitude of applied gate voltage can change the carrier density participating in transport through the channel, as well as the total current. The magnitude of applied source–drain voltage affects the charge‐carrier concentration gradient at the channel.^[^ ^218^ ^]^

The operation of FET with polycrystalline or amorphous semiconducting films can be divided into four main regions, as illustrated in Figure 18d: i) The device state before any bias voltage is applied. ii) The applied positive(negative) *V*_g_ drives electrons(holes) at the insulator/active layer interface. Some of the induced charge carriers fill in the trap states during the transport, the others are mobile carriers. The carrier density at the channel is related to the magnitude of *V*_g_ and capacitance (*C)* of the insulator. When there are sufficient mobile carriers at the active channel, *V*_g_ should be higher than *V*_Th_ (*V*_g_ − *V*_Th_ > 0). If only *V*_g_ is applied without source–drain voltage, the charge carriers at the channel are uniformly distributed. iii) When a small source–drain voltage (*V*_DS_ << *V*_g_) is applied, a linear gradient of carrier concentration is formed from source to drain electrode. *V*_DS_ will be increased to the point of *V*_DS_ = *V*_g_ − *V*_Th_, where a pinch‐off point exists at the transport channel adjacent to the drain (source) electrode. This is also called the depletion region, at which the difference between *V*_g_ and *V*_DS_ matches *V*_Th_. At this point, the electric field is not sufficient to induce additional mobile carriers to accumulate at the channel near the drain electrode. iv) When *V*_DS_ is further increased (*V*_DS_ > *V*_g_ − *V*_Th_), the same effect is enhanced, whereas the transport channel length is reduced. This is also called the saturation region, where the carriers can be driven across the narrow depletion zone and form a space‐charge‐limited current. Further boost of *V*_DS_ expands the depletion region, which cannot substantially increase source–drain current *I*_DS_ and saturates at a certain level called *I*_D,sat_.

Some of the important parameters for quantifying the FET performance include field‐effect mobility (*µ*_FE_), on/off current ratio, threshold voltage (*V*_Th_), and subthreshold swing (SS). *µ*_FE_ can explain the carrier transport capacity and can be extracted from the linear region of the transfer curve. On/off current ratio characterizes the ability to control channel current with *V*_g_ and can be calculated by the ratio of *I*_DS_ at on‐ and off‐state. *V*_Th_ describes the interface charge traps and *SS* shows the ability to switch on and off the device.^[^ ^235^, ^236^ ^]^

As discussed above, FETs are building blocks for modern electronic technologies. Through the discovery of promising semiconductor materials, innovative device structures, and electric circuit applications, FETs have successfully obtained a highly mature level of fabrication. Among the incumbent and emerging semiconductor materials, THPs offer excellent optoelectronic properties, including low effective mass, high carrier mobility, long charge‐carrier lifetime, and defect tolerance character.^[^ ^237^, ^238^, ^239^ ^]^ These attractive properties enabled the development of high‐performance, highly reliable THP‐based FETs over the last two decades, which will be discussed in the next section.

### 5.2 Recent Progress in THPs in FETs

#### 5.2.1 2D THP: (PEA)_2_SnI_4_

**Figure 19:**
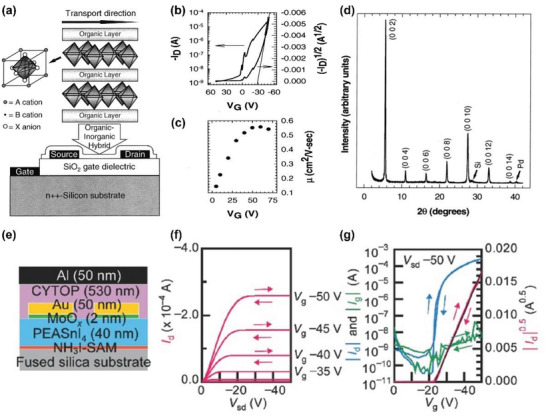
2D (PEA)_2_SnI_4_‐based FETs. a) Schematic illustration of a BGBC FET structure with a layered (PEA)_2_SnI_4_ channel. b) Corresponding transfer characteristics at *V*_D_ = −100 V. c) Corresponding extracted hole mobility. d) XRD spectrum of a layered (PEA)_2_SnI_4_ thin film. a–d) Reproduced with permission.^[^ ^240^ ^]^ Copyright 1999, American Association for the Advancement of Science. e) Schematic representation of a TGTC FET structure with (PEA)_2_SnI_4_ semiconducting channel, SAM‐treated substrate, and MoO*_x_* hole‐injection layer. f) Corresponding FET output curves. g) Corresponding transfer characteristics. e–g) Reproduced with permission.^[^ ^238^ ^]^ Copyright 2016, Wiley‐VCH.

2D layered THPs are highly stable with long‐chain organic bulky ligand in between the inorganic slabs to suppress ion migration and moisture penetration. Additionally, this family of materials is especially attractive for allowing versatile structure and tunable optoelectronic properties through compositional engineering of organic cations.^[^ ^241^, ^242^ ^]^ Because of these advantages, the first‐ever made perovskite transistor was based on 2D THP than any other 3D perovskites or LHPs. Almost two decades ago, Kagan et al. used (PEA)_2_SnI_4_ as the channel layer for BGBC FET, as illustrated in **Figure** **19**a.^[^ ^240^ ^]^ The device showed pure *p*‐channel characteristics with mobility of *µ*_h_ = 0.62 cm^2^ V^−1^ s^−1^ and a reasonable on/off current ratio of 10^4^ at room temperature (Figure 19b,c). This work highlighted the potential for fabricating MHP‐based FETs with a cost‐effective solution process. Through spin‐coating, the perovskite layer showed a high orientation in the (001) direction, which favors hole transport in the 2D crystal plane parallel to the device channel (Figure 19d). Additionally, the microstructures of perovskite film, including crystallinity and grain boundaries, are critical for device performance. Mitzi et al. introduced (PEA)_2_SnI_4_‐based BGBC FETs fabricated through melt process with silicon and polyimide substrates.^[^ ^243^ ^]^ The melt‐processed channel layer exhibited considerably enlarged grain size relative to spin‐coated films. Consequently, the fabricated device showed both improved saturation and linear regime hole mobilities of 2.6 and 1.7 cm^2^ V^−1^ s^−1^, respectively, at room temperature. Despite the initial success in 1999, research based on (PEA)_2_SnI_4_ accelerated only in the recent few years encouraged by remarkable advances in solar cells and LEDs. Recently, the performance of 2D THP‐based FETs remarkably developed through precise engineering of film morphology, device structure/interface engineering, crystal formation, and organic spacer synthesis.

Matsushima et al. first fabricated (PEA)_2_SnI_4_ by vacuum deposition on top of octadecyltricholorosilane (OTS)‐treated Si/SiO_2_ substrates.^[^ ^244^, ^245^ ^]^ By optimizing the growth rate and temperature of OTS on substrates, the film morphology was much improved and the fabricated FETs exhibited hole mobility of *µ*_h_ = 0.78 cm^2^ V^−1^ s^−1^ and an on/off current ratio of 10^5^ at room temperature. The same group proposed a significant development in solution‐processed (PEA)_2_SnI_4_ FETs by applying a series of optimization processes, including a self‐assembled monolayer (SAM)‐treatment on substrates and the addition of MoO*_x_* as a hole‐injection layer between the channel and the electrode (Figure 19e).^[^ ^238^ ^]^ Specifically, NH_3_I‐SAM treatment induced more well‐developed perovskite crystallites and reduced the amount of residual starting materials and ions. Also, TGTC configuration was formed with Cytop as a dielectric, which reduced resistance for charge carriers to travel through an alternating small‐bandgap inorganic layer and a large‐bandgap organic layer, and diminished hole trap density near the dielectric/perovskite interface. The optimized transistor exhibited high hole mobility of *µ*_h_ = 15 cm^2^ V^−1^ s^−1^ and a high on/off current ratio of 10^6^ at room temperature (Figure 19f,g). The authors also investigated the intrinsic *µ*_h_ of (PEA)_2_SnI_4_‐based FET by increasing *L* to reduce the contact resistance (*R*_c_) between the perovskite semiconductor and source/drain electrodes while using the same TCTG device configuration.^[^ ^246^ ^]^ With a negligible contribution of *R*_c_ relative to the total resistance, the hole mobility increased and saturated to a value of 26 cm^2^ V^−1^ s^−1^.

The performance of polycrystalline film devices is often intrinsically limited by grain boundaries, where carrier transport is slowed down or prohibited. To solve this issue, Matsushima et al. fabricated a BGBC transistor with exfoliated crystals of (PEA)_2_SnI_4_.^[^ ^247^ ^]^ Due to the reduced structural disorder and amount of grain boundaries, the fabricated device exhibited tremendously high hole mobility of 40 cm^2^ V^−1^ s^−1^ at room temperature. Despite such high performance, the restrictions on reproducing good quality crystals led to less than 1% yield of an operating transistor. Furthermore, their efforts in discovering the unique nature of (PEA)_2_SnI_4_ expanded to fabricating n‐type TGTC FETs.^[^ ^248^ ^]^ By inserting C_60_ as a buffer layer in between the semiconductor and the low‐work‐function metal (Al) electrodes, the authors presented an *n*‐type transistor with electron mobility of *µ*_e_ = 2.1 cm^2^ V^−1^ s^−1^ and an on/off current ratio of 10^4^ at room temperature. This work presented the possibility of ambipolar charge transport with (PEA)_2_SnI_4_ with a newly designed device structure.

Additionally, interface engineering was often approached to enhance the performance of (PEA)_2_SnI_4_ transistors. Zhang et al. fabricated a solution‐processed (PEA)_2_SnI_4_ transistor with PVA and cross‐linking poly(4‐vinylphenol) (CL‐PVP) as the dielectric and ITO as the gate electrode. The optimized device exhibited hole mobility of 0.28 and 0.33 cm^2^ V^−1^ s^−1^ in the forward and reverse voltage scan, and an on/off current ratio of 10^3^ at room temperature.^[^ ^249^ ^]^ The negligible hysteresis was highlighted due to the beneficial properties of PVA/CL‐PVP dielectric, including its high quality, good compatibility with perovskite, and suppressed ion migration in perovskite film.

**Figure 20:**
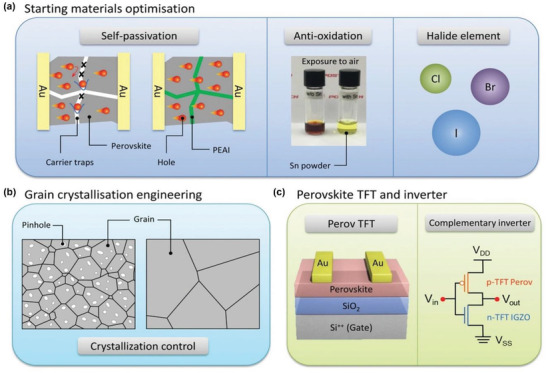
A systematic study on 2D (PEA)_2_SnI_4_ FET development. a) Optimization of starting materials: i) self‐passivation by adding slightly excess PEAI, ii) anti‐oxidation using metallic Sn powder, and iii) effect of halide element. b) Grain crystallization engineering through an adduct approach. c) Device fabrication, trace oxygen doping effect on FETs, and the first perovskite‐IGZO FET‐based complementary inverter. a–c) Reproduced with permission.^[^ ^131^ ^]^ Copyright 2020, Wiley‐ICH.

Although various approaches have enhanced the device performance of (PEA)_2_SnI_4_ FETs, the fragility of the material and other unexplored factors limited the overall operating device yield. Recently, Zhu et al. demonstrated a systematic study from starting materials to film and finally to FETs. The authors provided a set of universal methods for highly reproducible, high‐performance (PEA)_2_SnI_4_ FET with hole mobility of 3.51 cm^2^ V^−1^ s^−1^ and an on/off current ratio of 10^6^.^[^ ^131^ ^]^ The methods included self‐passivation of grain boundaries by using excess PEAI, control of grain crystallization by adding Lewis‐base adducts, and passivation of iodide vacancy through oxygen treatment (**Figure** **20**a). The passivation of grain boundaries and enlargement of grain size enabled the reproducibility and reliability of FET performance (Figure 20b). Notably, the authors fabricated the first perovskite‐based complementary inverters with optimized *p*‐channel (PEA)_2_SnI_4_ FET and *n*‐channel IGZO FETs, which showed a high gain of over 30 with an excellent noise margin (Figure 20c). Following this work, a wide range of solvent and additive engineering techniques have been applied to (PEA)_2_SnI_4_ FETs.^[^ ^106^, ^107^, ^250^, ^251^ ^]^

(PEA)_2_SnI_4_ is also one of the first THPs to be studied as a channel layer for a phototransistor.^[^ ^252^ ^]^ Phototransistors are a special type of transistors, as well as being categorized into photodetectors, with the capability to modulate channel conductance through electrical bias and irradiated photons.^[^ ^253^ ^]^ THPs are an especially attractive material for phototransistors, owing to their large optical absorption coefficient, long exciton diffusion length, and high charge carrier mobility.^[^ ^254^, ^255^, ^256^ ^]^ The detailed mechanism of the operation of halide perovskite phototransistors and photodetectors is thoroughly covered in a few previous review papers.^[^ ^33^, ^256^, ^257^, ^258^ ^]^ Throughout this work, some of the important achievements made in THP‐based phototransistors will be highlighted for several THPs. Zhu et al. significantly improved the photo detecting ability of (PEA)_2_SnI_4_‐based phototransistor by incorporating binary solvent of DMF and ethyl acetate (EA).^[^ ^250^ ^]^ The antisolvent addition smoothened the film formation through nucleation and consecutive oriented grain ripening, allowing efficient charge transport through full‐coverage film. Through amplified transistor function and photogate properties, the DMF/EA incorporated (PEA)_2_SnI_4_ phototransistor showed photoresponsivity of 1.6 × 10^5^ A W^−1^ and remarkably high detectivity of 3.2 × 10^17^ Jones.

#### 5.2.2 2D THPs: Synthesis of New Organic Spacers

**Figure 21:**
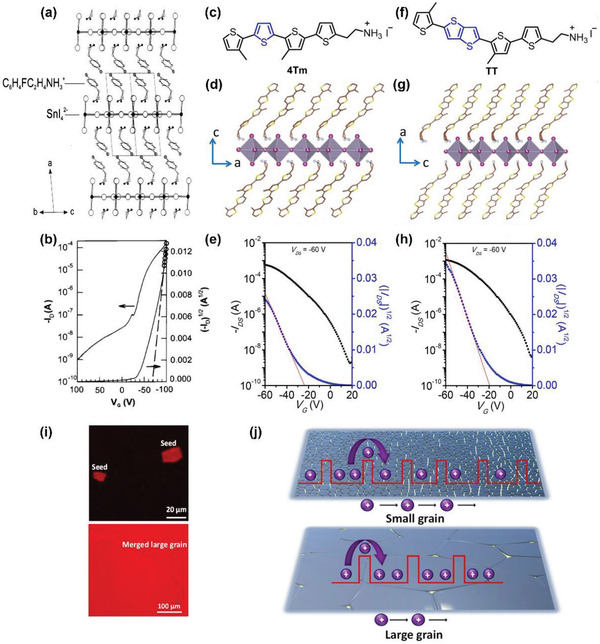
2D organic spacer synthesis and FET fabrication. a) (4‐FPEA)_2_SnI_4_ crystal structure, viewed along the (011) axis. b) Corresponding BGBC FET transfer characteristics. a,b) Reproduced with permission.^[^ ^27^ ^]^ Copyright 2001, American Chemical Society. c) Chemical structure of 4Tm. d) Side‐view of (4Tm)_2_SnI_4_ crystal structure. e) Corresponding FET transfer characteristics. f) Chemical structure of TT. g) Side‐view of (TT)_2_SnI_4_ crystal structure. h) Corresponding FET transfer characteristics. i) PL images of (TT)_2_SnI_4_ thin films after thermal annealing treatment at 155°C for 4 min (top) and 15 min (bottom) (scale bar = 20 µm). j) Schematic representation of carrier transport in small grain (top) and large grain (bottom)‐based (TT)_2_SnI_4_ thin film. c–j) Reproduced with permission.^[^ ^259^ ^]^ Copyright 2021, American Chemical Society.

Although extensive efforts were made to achieve high‐performance (PEA)_2_SnI_4_‐based FETs, most of these techniques focused on device engineering rather than the perovskite material itself. One of the earliest attempts in molecular engineering of 2D THPs was by Mitzi et al., who reported a study on tuning the electronic properties of (PEA)_2_SnI_4_ by fluorination of PEA ligand.^[^ ^27^ ^]^ By substituting a hydrogen atom on the phenyl ring of PEA with a fluorine atom, the authors composed m‐fluorophenylethyl ammonium tin iodide (m‐FPEA)_2_SnI_4_ (*m* = 2, 3, and 4, representing the position of the fluorine atom) (**Figure** **21**a). The different positions of fluorinated atoms induced subtle changes in Sn—I—Sn bond angle and fabricated BGBC FET hole mobilities: 156.41° and 0.56 cm^2^ V^−1^ s^−1^ (*m* = 4), 154.21° and 0.51 cm^2^ V^−1^ s^−1^ (*m* = 3), and 153.31°, and 0.24 cm^2^ V^−1^ s^−1^ (*m* = 2). (Figure 21b). Although the device performance did not show evident improvement, this study was one of the earliest approaches to associate cation molecular engineering with electronic devices.

Recently, Gao et al. proposed a breakthrough in novel organic spacer synthesis for 2D THPs.^[^ ^260^ ^]^ The authors modulated the highest occupied molecular orbital (HOMO) and lowest unoccupied molecular orbital energy levels of the ligands, which altered the overall quantum well structure as well as the charge injection. Inspired by organic semiconductors, polythiophene, for example, the authors featured linear *π*‐conjugated oligothiophene ligand, 2‐(3‴,4′‐dimethyl‐[2,2′:5′,2″:5″,2‴‐quaterthiophen]‐5‐yl)ethan‐1‐ammonium, also known as 4Tm (Figure 21c). The novel 4Tm ligand exhibited a suitable HOMO level that matched the work function of Au better than PEA or BA and reduced contact resistance to enhance hole injection. Also, 4Tm was incorporated into 2D THP layered structure (4Tm)_2_SnI_4_ and applied as a *p*‐channel layer to BGTC FETs, exhibiting hole mobility of *µ*_h_ = 2.32 cm^2^ V^−1^ s^−1^ (Figure 21d,e). This performance was achieved without additional treatments, which highlighted the excellence of the material itself. Moreover, the incorporation of 4Tm increased grain size up to a micrometer scale, which reduced charge scattering at grain boundaries. Furthermore, the device held remarkable air stability for up to 30 days, in contrast to the survival of pristine (PEA)_2_SnI_4_‐based FET for less than 1 day. The air stability greatly increased due to the hydrophobic and bulky properties of the *π*‐conjugated spacer, which can restrict oxygen and moisture penetration. The strong intermolecular interactions between the staggering conjugated 4Tm ligands also increased the intrinsic stability of the material.

The same group extended their studies to synthesizing thienothiophene derivative cation, 2‐(4′‐methyl‐5′‐(5‐(3‐methylthiophen‐2‐yl)thieno[3,2‐b]thiophen2‐yl)‐[2,2′‐bithiophen]‐5yl) ethan‐1‐ammonium, also known as TT (Figure 21f).^[^ ^259^ ^]^ Their champion device of (TT)_2_SnI_4_ exhibited hole mobility of *µ*_h_ = 9.35 cm^2^ V^−1^ s^−1^ and an on/off current ratio of 10^6^ (Figure 21g,h). The performance enhancement was induced by extended *π*‐conjugation and increased planarity of the TT ligand, which slowed down the nucleation process and significantly enlarged grain size to almost a millimeter scale (Figure 21i,j). Their variety of works in molecular engineering of organic spacers extended the potential application of highly stable 2D THPs in electronic devices.

#### 5.2.3 2D/3D Hybrid THPs

**Figure 22:**
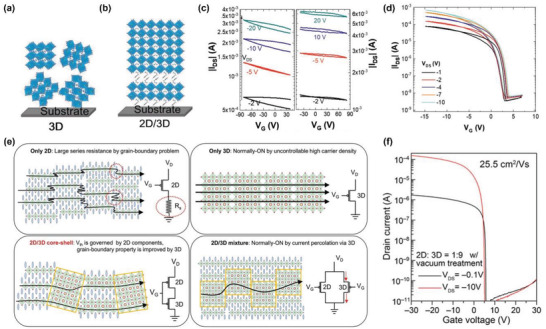
2D/3D hybrid FASnI_3_/(PEA)_2_SnI_4_ FET. Schematic representation of phase distribution and orientation in a) only 3D film. b) 2D/3D film. Transfer characteristics of BGBC FET with c) only 3D FASnI_3_ as an active layer d) 2D/3D hybrid (PEA)_2_SnI_4_/FASnI_3_ as an active layer with optimized thickness. a–d) Reproduced with permission.^[^ ^97^ ^]^ Copyright 2021, Wiley‐VCH. e) Schematic illustration of grain morphologies in cross‐sectional view of 2D (top‐left), 3D (top‐right), 2D/3D core–shell (bottom‐left) and 2D/3D mixture (bottom‐right) Sn‐based perovskite thin films. f) Transfer characteristics of 2D/3D core–shell FET with 2D/3D ratio of 1:9 and vacuum treatment. e,f) Reproduced under the terms of the Creative Commons CC‐BY license.^[^ ^261^ ^]^ Copyright 2022, The Authors. Published by Wiley‐VCH.

From the first works of THPs to the recent extension of studies, 2D THPs demonstrated remarkable enhancement in device performance and air stability. Despite its progress, the fundamental intrinsic limits of 2D perovskites are yet to be solved. Due to the quantum and dielectric confinement effect in 2D perovskites, the charge transport is mostly confined in the corner‐sharing inorganic octahedral cage layer, whereas the charge carrier perpendicular to the inorganic layer is strongly restricted. Contrary to 2D perovskites, 3D perovskites have a higher potential for faster charge transport in absence of the quantum and dielectric confinement effect. However, 3D THPs are notorious for high trap density and high self‐*p*‐doping. Until the works of Shao et al., there were no reports that used 3D THPs as the semiconducting layer for FETs. The authors successfully reduced the hole density of 3D FASnI_3_ by incorporating a small amount of 2D (PEA)_2_SnI_4_.^[^ ^97^ ^]^ The addition of (PEA)_2_SnI_4_ greatly enhanced the crystallinity and orientation of the 3D perovskite phase, which was critical for FET application (**Figure** **22**a,b). The highly crystalline 2D/3D film exhibited a much smaller amount of trap states than its 3D counterpart. The reduced trap states were particularly Sn vacancies, which are the dominant source of high *p*‐doping in 3D Sn‐based perovskite (Figure 22c). Through the control of crystallinity and orientation, along with reduced trap density, (PEA)_2_SnI_4_‐incorporated FASnI_3_‐based FET exhibited hole mobility of 0.21 cm^2^ V^−1^ s^−1^ and an on/off current ratio of 10^4^ (Figure 22d).

Recently, Kim et al. also reported a study on the incorporation of 2D (PEA)_2_SnI_4_ into 3D FASnI_3_ from an alternative perspective.^[^ ^261^ ^]^ The authors presented a new concept of 2D–3D core–shell structure, where the 3D core is fully isolated by the 2D counterpart. The suggested structure provided two major benefits: independent control of *V*_Th_ by 2D component and significantly improved grain boundary resistance by the 2D/3D interface. The 2D (PEA)_2_SnI_4_ FETs suffer from abnormally saturated on‐current caused by large series resistance at the grain boundaries, whereas 3D THP‐based FET exhibits the excessive carrier density that only permits on‐state (Figure 22e). This 2D–3D core–shell simultaneously solves both issues by first controlling the on‐state by 2D component and forming a smoother match between 2D/3D lattices. By adding SnF_2_ to facilitate the formation of core–shell structure and vacuum treatment, (PEA)_2_SnI_4_‐FASnI_3_‐based FET showed hole mobility of 25 cm^2^ V^−1^ s^−1^ and an on/off current ratio of 10^6^ (Figure 22f). Additionally, the authors combined the optimized *p*‐channel FET with *n*‐channel IGZO FET to fabricate CMOS with a gain of over 200. Moreover, Shen et al. proposed a synthesis of 2D incorporated 3D Cs‐based THP crystal. A millimeter‐sized (PEA)_2_CsSn_2_I_7_ crystal was applied as a channel layer to BCBG FET, which exhibited hole mobility of 34 cm^2^ V^−1^ s^−1^ at a low temperature of 77 K.^[^ ^262^ ^]^

In another perspective, FASnI_3_ itself has high hole concentration and hole mobility due to self *p*‐doping nature of 3D THP perovskite, which is outstanding to be used as a phototransistor. Recently, Liu et al. developed a high‐performance FASnI_3_‐based phototransistor with a high responsivity of ≈10^5^ A W^−1^ at a low operating voltage.^[^ ^263^ ^]^ The authors also incorporated FASnI_3_/PEDOT:PSS heterojunction due to the enhanced photogate effect from the heterojunction, reaching a maximum responsivity of 2.6 × 10^6^ A W^−1^.^[^ ^264^ ^]^

#### 5.2.4 3D THP: CsSnI_3_

**Figure 23:**
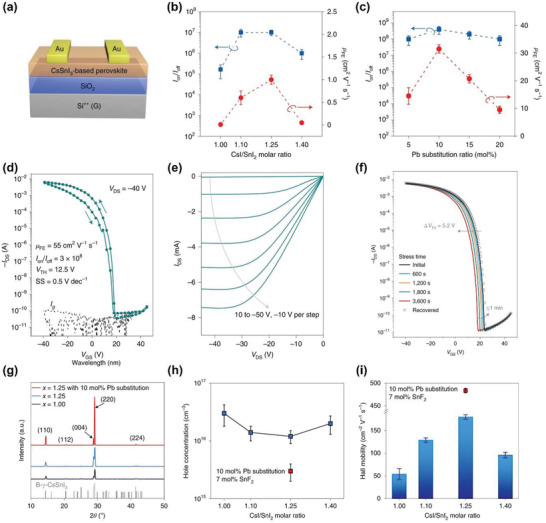
3D CsSnI_3_‐based FET. a) Schematic illustration of BGTC TFT structure. b) *µ*_FE_ and *I*_on_/*I*_off_ of CsSnI_3_‐based TFTs with different molar ratios of CsI/SnI_2_. c) *µ*_FE_ and *I*_on_/*I*_off_ of CsI‐rich (*x* = 1.25) precursors with various ratios of Pb substitution. d) Transfer characteristics of optimized CsSnI_3_‐based TFT (*x* = 1.25, 10 mol% Pb substitution, and 7 mol% of SnF_2_). e) Corresponding output curves. f) Transfer curves of one optimized CsSnI_3_‐based TFT under negative bias–stress measurement for various durations and the recovery behavior at *V*_GS_ = *V*_DS_ = −40 V. g) XRD patterns of perovskite films deposited from precursors with different ratios of CsI/SnI_2_ and that from CsI‐rich (*x* = 1.25) precursor with 10 mol% Pb substitution and 7 mol% SnF_2_. Hall measurement derived parameters, including h) hole concentration of thin films deposited from precursors with different CsI/SnI_2_ ratios (without SnF_2_) and that from optimized CsI‐rich (*x* = 1.25), 10 mol% Pb substitution, and 7 mol% SnF_2_ precursor and i) corresponding Hall mobility. a–i) Reproduced with permission.^[^ ^20^ ^]^ Copyright 2022, Nature Publishing Group.

Groundbreaking development in *p*‐type tin perovskite‐based transistors was recently accomplished by our group through developing a 3D CsSnI_3_‐based channel layer.^[^ ^20^ ^]^ The fabricated champion device achieved a record performance with hole mobility over 50 cm^2^ V^−1^ s^−1^ and on/off current ratios exceeding 10^8^ (**Figure** **23**a–e). These values meet the requirements for the backplane of high‐end displays and integrated logic circuits. Additionally, the optimized CsSnI_3_‐based TFTs highlighted high operational stability and reproducibility (Figure 23f). Using bias–stress test, the authors discovered a strikingly fast performance recovery within 1 min after the measurement. This unique feature of CsSnI_3_‐based TFTs is a drastic improvement from the commercialized *p*‐type polycrystalline Si or *n*‐type metal oxide‐based TFTs, which require hour‐long recovery time or extra thermal treatments.^[^ ^265^ ^]^ The authors developed a series of methods to accentuate the fast carrier transport and compensate for the fundamental self‐*p*‐doping issues of 3D THPs. The methods include SnF_2_ incorporation in CsI‐rich precursor and a small portion of SnI_2_ substitution with PbI_2_. SnF_2_ incorporation has already demonstrated efficient suppression of Sn^2+^ oxidation and control of crystallization in THPs.^[^ ^75^, ^86^, ^89^ ^]^ Additionally, CsSnI_3_ solution with slight excess CsI improved film morphology, as well as electrical performance. Moreover, due to the lower Lewis acidity of Pb^2+^ compared with Sn^2+^, a small amount of Pb substitution in the precursor slowed down the conversion to the perovskite phase, ultimately improving the film crystalline quality (Figure 23g).^[^ ^266^, ^267^, ^268^, ^269^, ^270^ ^]^ Hall‐effect measurements were conducted to further evaluate the electrical properties of the film. CsI‐rich films demonstrated lower hole concentration but significantly increased Hall mobilities due to improved film uniformity/crystallinity and reduced defect density, such as *V*_Sn_ (Figure 23h,i). This series of techniques can be a stepping stone toward the development of high‐performance *p*‐type THP‐based transistors and complementary electronics.

#### 5.2.5 3D THP: MASnI_3_

**Figure 24:**
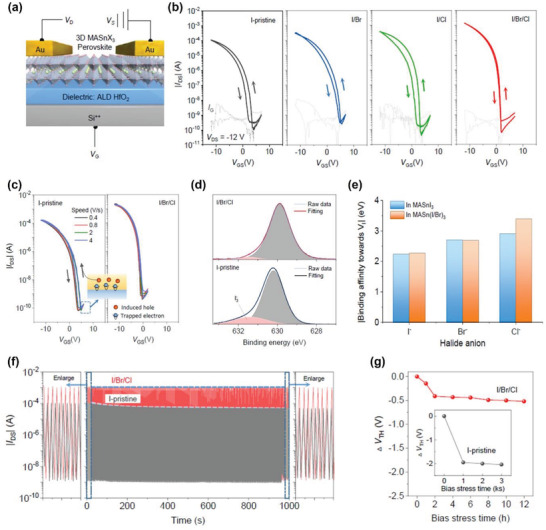
3D MASnI_3_‐based FET: Removal of hysteresis through anion engineering. a) Transistor structure. b) Transfer characteristics with different perovskite channel layers. c) Transfer characteristics of I‐pristine and I/Br/Cl transistors measured at various scan speeds. d) I 3*d*_3/2_ core level XPS spectra. e) Calculated binding affinities of halide anions with *V*_I_‐site in MASnI_3_ and MASn(I/Br)_3_. f) On/off switching stability of I‐pristine and I/Br/Cl transistors. g) Variation of *V*_Th_ under bias–stress. a–g) Reproduced with permission.^[^ ^95^ ^]^ Copyright 2022, Nature Publishing Group.

The first demonstration of *p*‐channel perovskite transistors based on 3D MASnI_3_ was recently reported as shown in **Figure** **24**a.^[^ ^95^ ^]^ By rationalizing the effects of halide (I/Br/Cl) anion engineering on the improvement of film morphology and suppression of tin/iodide vacancy, the authors developed high‐performance hysteresis‐free TFTs with a high hole mobility of 20 cm^2^ V^−1^ s^−1^ and on/off ratio exceeding 10^7^ (Figure 24b). The optimized device demonstrated a threshold voltage of 0 V, which is an ideal enhancement mode where no applied bias voltage is required to turn off the transistor. This behavior is a highly desirable trait for simplifying circuit design and minimizing power consumption in electronic applications.^[^ ^236^, ^271^ ^]^ The authors revealed that contrary to previous studies on LHPs, ion migration had a negligible contribution to hysteresis in THP‐based transistors, but minority carrier traps induced by iodine vacancy *V*_I_ were the primary cause. The deep electron traps induced by *V*_I_ defects were significantly reduced by Br/Cl co‐substitution with strong binding affinities to *V*_I_ sites and stabilized the overall lattice structure (Figure 24c–e). Furthermore, operational stability is another crucial figure of merit for practical applications. Through on/off switching stability and bias–stress stability test, the I‐pristine device suffered from serious carrier trapping, whereas the I/Br/Cl optimized device showed much‐improved stability, almost comparable to those of stable organic and amorphous silicon‐based FETs (Figure 24f,g).^[^ ^239^, ^272^ ^]^ By further combining the optimized *p*‐channel perovskite FETs with *n*‐channel IGZO FETs, the authors developed monolithically integrated high‐gain complementary inverters and highlighted their high compatibility and processability for electronic applications.

#### 5.2.6 Sn^2+^‐Free THP: Vacancy‐Ordered Double Perovskite

**Figure 25:**
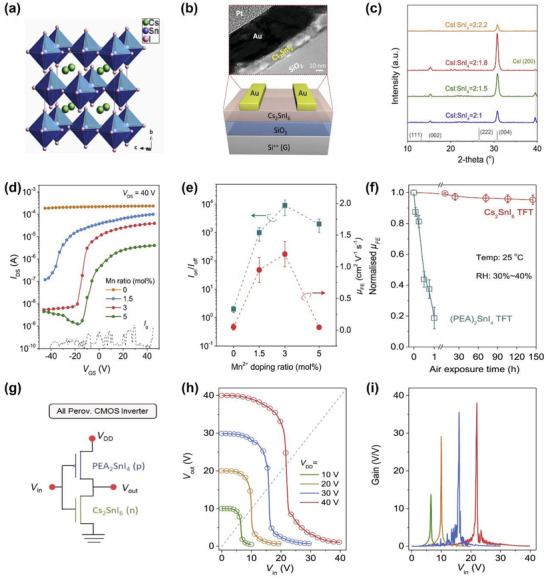
Sn^2+^‐free perovskite FET. a) The crystal lattice structure of vacancy‐ordered double perovskite Cs_2_SnI_6_. b) TFT structure and corresponding cross‐sectional TEM image. c) X‐ray diffraction peak patterns of Cs_2_SnI_6_ as a function of the CsI:SnI_4_ ratio in the precursor solution. d) Transfer characteristics of Cs_2_SnI_6_ TFT with various ratios of Mn^2+^ doping. e) Summary of electron mobility *µ*_e_ and *I*_on_/*I*_off_ of TFTs. f) Normalized *µ*_FE_ of Sn^2+^‐based (PEA)_2_SnI_4_ TFT and Sn(IV)‐based Cs_2_SnI_6_ TFTs as a function of ambient air exposure time. g) Schematic diagram of CMOS inverter with combined p‐channel (PEA)_2_SnI_4_ TFT and n‐channel Mn^2+^‐doped Cs_2_SnI_6_ TFT. Corresponding h) voltage transfer and i) gain voltage curves at different *V*_DD_s. a–i) Reproduced with permission.^[^ ^92^ ^]^ Copyright 2022, Cell Press.

Despite the extensive efforts to improve the air stability of THP film, its application in an inert atmosphere remains limited.^[^ ^69^, ^273^ ^]^ An alternative approach to developing an air‐stable THP device is to modulate the perovskite material composition itself. Vacancy‐ordered halide double perovskites are attractive alternatives for their toxic lead and unstable Sn^2+^‐based counterparts.^[^ ^91^, ^93^, ^274^ ^]^ Despite the early discovery of this composition and continuous theoretical studies, the application on electronic devices was rare until a recent report by Liu et al.^[^ ^92^ ^]^ The authors demonstrated a synergetic solution process method to modulate the film quality of Cs_2_SnI_6_ and its feasibility in FETs (**Figure** **25**a,b). The incorporation of slight excess SnI_4_ in a mixed DMF/DMSO solvent‐based precursor generated improved crystallinity, uniform morphology, and high electrical properties (Figure 25c). Also, the authors used Mn^2+^ doping to further tune the electrical properties for TFT application and achieved electron mobility of *µ*_e_ = 1.2 cm^2^ V^−1^ s^−1^ and an on/off current ratio of 10^4^ (Figure 25d,e). Most importantly, Cs_2_SnI_6_‐based TFT exhibited ambient air stability with reliable operation for more than 1 week, which is an impressive improvement from previous 2D THP‐based TFTs (Figure 25f). The dramatic enhancement in air stability benefited from the attractive chemical compositions of Cs_2_SnI_6_, including strong Sn—I covalent bonding and a stable Sn tetravalent state.^[^ ^275^, ^276^ ^]^ Thus, the authors highlighted Cs_2_SnI_6_ as a promising alternative material for high‐performance and high‐stability THP‐based transistors. Furthermore, the authors expanded the potential electronic applications of *n*‐channel Cs_2_SnI_6_ TFT by combining it with *p*‐channel (PEA)_2_SnI_4_ TFT to develop the first all‐perovskite complementary inverter with a high‐gain voltage over 38 (Figure 25g–i).

### 5.3 Summary and Future Perspective

**Figure 26:**
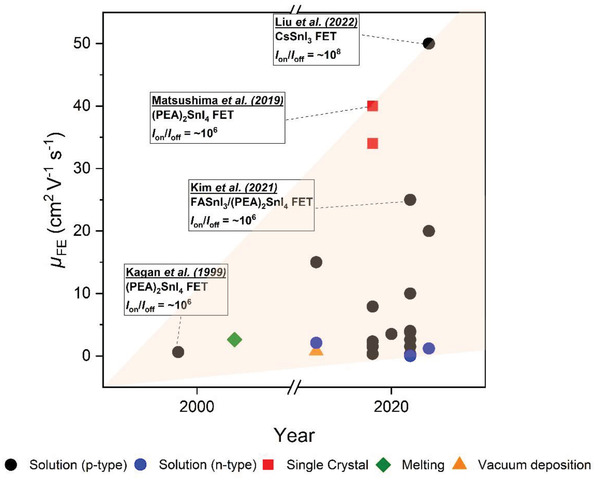
Development of THP‐based FETs over the past two decades.

From the first‐ever reported (PEA)_2_SnI_4_‐based transistor to recent success in CsSnI_3_‐based transistor, two decades of research in THPs have shown a striking increase in device performance, along with efforts to enhance its stability. **Table** **6** and **Figure** **26** illustrate the extension of studies on THP‐based FETs with major milestones from 2D to 3D materials, achieved by multiple research groups. The majority of the reported devices are solution‐processed, and only a few used other methods, such as exfoliated crystals grown from solution or melting process from grown crystals. Only one report has used vacuum deposition to fabricate the THP layer. Each of these methods has its advantages and disadvantages. For example, solution process is a simple and cost‐effective fabrication method, advantageous for industrial applications. However, the solution process has relatively low reproducibility because the quality of the solution has a critical effect on the fabricated film and patterning issue. The recent achievement of high‐performance THP‐based FETs with hole mobility as high as over 50 cm^2^ V^−1^ s^−1^ by Liu et al. was also fabricated via a solution process. Through this method, the work highlighted a great potential in the industrial application of THP‐based *p*‐type transistors, with comparable performance with commercialized *n*‐type transistors. The next step in the development of THP‐based FETs will be to greatly increase the reproducibility of high performances through vacuum deposition. As shown through the success of the OLED industry, vacuum deposition can fabricate highly reproducible devices in mass production. THP devices may follow the pathway toward commercialization with such high performance and reproducibility.

**Table 6:** Summary of device performance of THP‐based transistors

Material	Dielectric/contact	Structure	Deposition/treatment	*µ*_h_ [cm^2^ V^−1^ s^−1^]	*µ*_e_ [cm^2^ V^−1^ s^−1^]	*I*_on_/*I*_off_	*T*	Ref.
(PEA)_2_SnI_4_	SiO_2_/Au	BGBC	–	0.62		≈10^4^	R.T.	[240]
PEASnI_4_	Cytop/Au	TCTG	NH_3_I‐SAM, MoO*_x_* HIL	15		≈10^6^	R.T.	[238]
PEASnI_4_	SiO_2_/Au	BGTC	Vacuum deposition, OTS‐SAM	0.78		≈10^5^	R.T.	[244]
PEASnI_4_	Cytop/Al	TCTG	NH_3_I‐SAM, C60 EIL		2.1	≈10^4^	R.T.	[248]
(PEA)_2_SnI_4_	SiO_2_/Au	BGTC	MoO*_x_* HIL	7.9		≈10^7^	R.T.	[135]
(PEA)_2_SnI_4_	SiO_2_/Au	BGBC	Single crystal, MoO*_x_* HIL	40		≈10^6^	R.T.	[247]
(PEA)_2_SnI_4_	PVA/Cl‐PVP/Au	BGTC	Polymer dielectric	0.28/0.33		10^2^≈10^3^	R.T.	[249]
(PEA)_2_SnI_4_/PVP	Cl‐PVP/Au	BGTC	Polymer dielectric	0.31		≈10^3^	R.T.	[277]
(PEA)_2_SnI_4_/PVP, PEO	Cl‐PVP/Au	BGTC	Polymer mixed with precursor	0.013	0.0068	≈10^4^	R.T.	[278]
(PEA)_2_Sn*_x_* Pb_1‐_ *_x_* I_4_	PVA/Cl‐PVP/Au	BGTC	Sn/Pb ratio	0.02 (*x* = 0.7)		≈10^2^	R.T.	[279]
(PEA)_2_SnI_4_/CNT	SiO_2_/Au	BGTC	CNT mixed with precursor	1.51		≈10^5^	R.T.	[280]
(PEA)_2_SnI_4_	SiO_2_/Au	BGTC	Oxygen treatment	3.51		≈10^6^	R.T.	[131]
(PEA)_2_SnI_4_/CB/EA	SiO_2_/Au	BGTC	Binary solvent	3.8		≈10^5^	R.T.	[250]
(PEA)_2_SnI_4_/Urea	SiO_2_/Au	BGTC	Urea mixed with precursor	4		≈10^5^	R.T.	[251]
(PEA)_2_SnI_4_/CuI	SiO_2_/Au	BGTC	CuI mixed with precursor	2.61		≈10^6^	R.T.	[106]
(4‐MeO‐PEA)_2_SnI_4_	SiO_2_/Polyimide/Au	BGBC	Melt process	2.6		≈10^6^	R.T.	[243]
(m‐FPEA)_2_SnI_4_	SiO_2_/Pd	BGBC	–	0.2 ≈ 0.6		≈10^5^	R.T.	[27]
(PEA)_2_CsSnI_7_	SiO_2_/Cr/Au	BGBC	Single crystal	34		–	77 K	[262]
(4Tm)_2_SnI_4_	SiO_2_/Au	BGTC	–	2.32		≈10^6^	R.T.	[260]
(TT)_2_SnI_4_	SiO_2_/Au	BGTC	–	10		≈10^6^	R.T.	[259]
(STm)_2_SnI_4_	SiO_2_/Au	BGTC	–	1.52		≈10^6^	R.T.	[281]
FASnI_3_/(PEA)_2_SnI_4_	PMMA/Al_2_O_3_/Au	TGBC	–	0.21		≈10^4^	R.T.	[97]
FASnI_3_/(PEA)_2_SnI_4_	SiO_2_/Pt	BGBC	Vacuum treatment	25		≈10^6^	R.T.	[261]
MASnI_3_	HfO_2_/Au	BGTC	I/Br/Cl anion engineering	20		≈10^7^	R.T	[95]
Cs_2_SnI_6_	SiO_2_/Au	BGTC	Mn^2+^ doping		1.2	≈10^4^	R.T.	[92]
CsSnI_3_	SiO_2_/Au	BGTC	Small Pb substitution	55		≈10^8^	R.T.	[20]

Channel layers are solution‐processed unless mentioned otherwise, *µ*_h_: Hole mobility, *µ*_e_: Electron mobility, *I*_on_/*I*_off_: On/off current ratio, T: Temperature

## 6 Conclusion

To conclude, we reviewed the structural property relationship, device performance, and stability of several THPs (MASnI_3_, FASnX_3_, and CsSnX_3_) and presented their current issues and perspective opportunities. Thus far, despite the high device performance of the potential of THPs, there has been no report showing the ambient stability for the commercialization level. Therefore, to further improve our standing on device performance and its stability, we must indeed pay attention to overcoming several challenges including i) pure and non‐oxidized initial chemicals and non‐oxidizing solvents, ii) fully understanding of film formation dynamics, iii) precise control of cation and anion defects in the film, iv) suppression of Sn^2+^ oxidation, v) commercial‐grade device operation stability in the air ambient, and vi) better energy band alignment. The vital difference between THPs and its Pb‐based counterparts originated from the instability of Sn^2+^ in 3D structure and the tendency to realize *V*_Sn_. Thus, to aid our discussion, we hence also review the mechanism governing Sn oxidation, as well as the efficient route to reduce Sn^4+^ and the encapsulation approach to alleviating the poor stability, especially in ambient air. It is understood that the under‐performance of THP‐based devices is due to the formation of *V*_Sn_; thus, a greater understanding of the Sn oxidation would lead to better‐performing THP‐based devices. To date, numerous techniques have been introduced to reduce such effects, namely, the use of additives to act as reducing agents, control crystallization, partial ion substitution, and reduced dimensionality, and we indeed believe that an important breakthrough will be reported in the near future. Henceforward, finding new, stable, and effective solvents for THP‐based devices, having a deep understanding of the Sn chemistry via film formation, interfacial engineering to better match the energy bands of THPs, and stabilizing Sn^2+^ through device fabrication procedures are greatly important. Alternatively, the implementation of high‐performing devices using vacuum‐deposited THPs can be another breakthrough to fundamentally escape from the issues of various solubility of precursors and additives or stable and effective solvents. Last, although huge progress has recently been made in THP‐based optoelectronics devices, a deep understanding of the photo‐physics and photo‐chemistry processes is hardly obtained. Many approaches have been introduced to improve the performance of those devices including employing reducing agents, morphological control, compositional engineering, device engineering as well as interfacial engineering, but the impact of these approaches on the structural properties and device performance requires more systematic investigation. This will ultimately improve not only device performance but also device stability, which is critical to commercialization.

## Conflict of Interest

The authors declare no conflict of interest.

## References

[1] Z. Wang , Z. Zhang , L. Xie , S. Wang , C. Yang , C. Fang , F. Hao , Adv. Opt. Mater. 2022, 10, 2101822.

[2] H. P. Kim , M. Vasilopoulou , H. Ullah , S. Bibi , A. E. X. Gavim , A. G. Macedo , W. J. da Silva , F. K. Schneider , A. A. Tahir , M. A. M. Teridi , Nanoscale 2020, 12, 7641. 32207472 10.1039/c9nr10745b

[3] M. Vasilopoulou , M. Yusoff , M. Daboczi , J. Conforto , A. E. X. Gavim , W. J. da Silva , A. G. Macedo , A. Soultati , G. Pistolis , F. K. Schneider , Y. Dong , P. Jacoutot , G. Rotas , J. Jang , G. C. Vougioukalakis , C. L. Chochos , J.‐S. Kim , N. Gasparini , Nat. Commun. 2021, 12, 4868. 34381038 10.1038/s41467-021-25135-z PMC8357948

[4] H. P. Wang , S. Li , X. Liu , Z. Shi , X. Fang , J. H. He , Adv. Mater. 2021, 33, 2003309. 10.1002/adma.202003309 33346383

[5] L. Zhang , J. Miao , J. Li , Q. Li , Adv. Funct. Mater. 2020, 30, 2003653.

[6] H. Dong , C. Zhang , X. Liu , J. Yao , Y. S. Zhao , Chem. Soc. Rev. 2020, 49, 951. 31960011 10.1039/c9cs00598f

[7] M. Vasilopoulou , B. S. Kim , H. P. Kim , W. J. da Silva , F. K. Schneider , M. A. Mat Teridi , P. Gao , A. R. b. Mohd Yusoff , M. K. Nazeeruddin , Nano Lett. 2020, 20, 5081. 32492348 10.1021/acs.nanolett.0c01270

[8] H. Park , C. Ha , J.‐H. Lee , J. Mater. Chem. A 2020, 8, 24353.

[9] J. Tian , Q. Xue , Q. Yao , N. Li , C. J. Brabec , H. L. Yip , Adv. Energy Mater. 2020, 10, 2000183.

[10] J. Jeong , M. Kim , J. Seo , H. Lu , P. Ahlawat , A. Mishra , Y. Yang , M. A. Hope , F. T. Eickemeyer , M. Kim , Y. J. Yoon , I. W. Choi , B. P. Darwich , S. J. Choi , Y. Jo , J. H. Lee , B. Walker , S. M. Zakeeruddin , L. Emsley , U. Rothlisberger , A. Hagfeldt , D. S. Kim , M. Grätzel , J. Y. Kim , Nature 2021, 592, 381. 33820983 10.1038/s41586-021-03406-5

[11] D. H. Kang , N. G. Park , Adv. Mater. 2019, 31, 1805214.

[12] E. Aydin , M. De Bastiani , S. De Wolf , Adv. Mater. 2019, 31, 1900428. 10.1002/adma.201900428 31062907

[13] C. M. Wolff , P. Caprioglio , M. Stolterfoht , D. Neher , Adv. Mater. 2019, 31, 1902762. 10.1002/adma.201902762 31631441

[14] W. W. Liu , T. H. Wu , M. C. Liu , W. J. Niu , Y. L. Chueh , Adv. Mater. Interfaces 2019, 6, 1801758.

[15] R. K. Raman , S. A. Gurusamy Thangavelu , S. Venkataraj , A. Krishnamoorthy , Renewable Sustainable Energy Rev. 2021, 151, 111608.

[16] W. Gao , C. Chen , C. Ran , H. Zheng , H. Dong , Y. Xia , Y. Chen , W. Huang , Adv. Funct. Mater. 2020, 30, 2000794.

[17] P. Mariyappan , T. H. Chowdhury , S. Subashchandran , I. Bedja , H. M. Ghaithan , A. Islam , Sustainable Energy Fuels 2020, 4, 5042.

[18] R. Chiara , M. Morana , L. Malavasi , ChemPlusChem 2021, 86, 879. 34126001 10.1002/cplu.202100191

[19] M. Abdel‐Shakour , T. H. Chowdhury , K. Matsuishi , I. Bedja , Y. Moritomo , A. Islam , Sol. RRL 2021, 5, 2000606.

[20] A. Liu , H. Zhu , S. Bai , Y. Reo , T. Zou , M.‐G. Kim , Y.‐Y. Noh , Nat. Electron. 2022, 5, 78.

[21] D. E. Scaife , P. F. Weller , W. G. Fisher , J. Solid State Chem. 1974, 9, 308.

[22] S. J. Clark , C. D. Flint , J. D. Donaldson , J. Phys. Chem. Solids 1981, 42, 133.

[23] D. E. Parry , M. J. Tricker , J. D. Donaldson , J. Solid State Chem. 1979, 28, 401.

[24] K. Yamada , Y. Kuranaga , K. Ueda , G. Shusaku , O. Tsutomu , F. Yoshihiro , Bull. Chem. Soc. Jpn. 1998, 71, 127.

[25] K. Yamada , T. Matsui , T. Tsuritani , T. Okuda , S. Ichiba , Z. Naturforsch., A: Phys. Sci. 1990, 45, 307.

[26] K. Yamada , S. Nose , T. Umehara , T. Okuda , S. Ichiba , Bull. Chem. Soc. Jpn. 1988, 61, 4265.

[27] D. B. Mitzi , C. D. Dimitrakopoulos , L. L. Kosbar , Chem. Mater. 2001, 13, 3728.

[28] C. C. Stoumpos , C. D. Malliakas , M. G. Kanatzidis , Inorg. Chem. 2013, 52, 9019. 23834108 10.1021/ic401215x

[29] H. Dong , C. Ran , W. Gao , N. Sun , X. Liu , Y. Xia , Y. Chen , W. Huang , Adv. Energy Mater. 2022, 12, 2102213.

[30] T. Imran , S. Rauf , H. Raza , L. Aziz , R. Chen , S. Liu , J. Wang , M. A. Ahmad , S. Zhang , Y. Zhang , Z. Liu , W. Chen , Adv. Energy Mater. 2022, 12, 2200305.

[31] M. Li , F. Li , J. Gong , T. Zhang , F. Gao , W.‐H. Zhang , M. Liu , Small Struct. 2022, 3, 2100102.

[32] J. Wang , Z. Gao , J. Yang , M. Lv , H. Chen , D.‐J. Xue , X. Meng , S. Yang , Adv. Energy Mater. 2021, 11, 2102131.

[33] F. Cao , L. Li , Adv. Funct. Mater. 2021, 31, 2008275.

[34] Y. Gao , Y. Pan , F. Zhou , G. Niu , C. Yan , J. Mater. Chem. A 2021, 9, 11931.

[35] G. Kieslich , S. Sun , A. K. Cheetham , Chem. Sci. 2015, 6, 3430. 28706705 10.1039/c5sc00961h PMC5492664

[36] D.‐F. Weng , Z.‐M. Wang , S. Gao , Chem. Soc. Rev. 2011, 40, 3157. 21283846 10.1039/c0cs00093k

[37] V. M. Goldschmidt , Naturwissenschaften 1926, 14, 477.

[38] D. B. Mitzi , J. Chem. Soc., Dalton Trans. 2001, 1.

[39] R. D. Shannon , Acta Crystallogr., Sect. A: Found. Adv. 1976, 32, 751.

[40] M. A. Green , A. Ho‐Baillie , H. J. Snaith , Nat. Photonics 2014, 8, 506.

[41] G. Kieslich , S. Sun , A. K. Cheetham , Chem. Sci. 2014, 5, 4712.

[42] P. Gao , A. R. Bin Mohd Yusoff , M. K. Nazeeruddin , Nat. Commun. 2018, 9, 5028. 30487520 10.1038/s41467-018-07382-9 PMC6261957

[43] J.‐P. Correa‐Baena , M. Saliba , T. Buonassisi , M. Grätzel , A. Abate , W. Tress , A. Hagfeldt , Science 2017, 358, 739. 29123060 10.1126/science.aam6323

[44] W. Gao , C. Ran , J. Li , H. Dong , B. Jiao , L. Zhang , X. Lan , X. Hou , Z. Wu , J. Phys. Chem. Lett. 2018, 9, 6999. 30499301 10.1021/acs.jpclett.8b03194

[45] D. Mitzi , S. Wang , C. Feild , C. Chess , A. Guloy , Science 1995, 267, 1473. 17743545 10.1126/science.267.5203.1473

[46] J. Even , L. Pedesseau , J. M. Jancu , C. Katan , Phys. Status Solidi RRL 2014, 8, 31.

[47] Y. Takahashi , R. Obara , Z.‐Z. Lin , Y. Takahashi , T. Naito , T. Inabe , S. Ishibashi , K. Terakura , Dalton Trans. 2011, 40, 5563. 21494720 10.1039/c0dt01601b

[48] P. Umari , E. Mosconi , F. De Angelis , Sci. Rep. 2014, 4, 4467. 24667758 10.1038/srep04467 PMC5394751

[49] G. Laurita , D. Fabini , C. Stoumpos , M. Kanatzidis , R. Seshadri , Chem. Sci. 2017, 8, 5628. 28989600 10.1039/c7sc01429e PMC5621007

[50] E. S. Parrott , R. L. Milot , T. Stergiopoulos , H. J. Snaith , M. B. Johnston , L. M. Herz , J. Phys. Chem. Lett. 2016, 7, 1321. 26990282 10.1021/acs.jpclett.6b00322

[51] V. K. Sharma , V. Kanchana , M. K. Gupta , R. Mittal , J. Solid State Chem. 2020, 290, 121541.

[52] M. Dawson , Ribeiro, C. , Morelli, M. R. , Mater. Res. 2022, 25, e20210441.

[53] D. B. Mitzi , K. Liang , J. Solid State Chem. 1997, 134, 376.

[54] E. C. Schueller , G. Laurita , D. H. Fabini , C. C. Stoumpos , M. G. Kanatzidis , R. Seshadri , Inorg. Chem. 2018, 57, 695. 29278493 10.1021/acs.inorgchem.7b02576

[55] S. Kahmann , O. Nazarenko , S. Shao , O. Hordiichuk , M. Kepenekian , J. Even , M. V. Kovalenko , G. R. Blake , M. A. Loi , ACS Energy Lett. 2020, 5, 2512.

[56] M. Rameez , E. Y.‐R. Lin , P. Raghunath , S. Narra , D. Song , M.‐C. Lin , C.‐H. Hung , E. W.‐G. Diau , ACS Appl. Mater. Interfaces 2020, 12, 21739. 32295339 10.1021/acsami.0c03704

[57] I. Chung , J.‐H. Song , J. Im , J. Androulakis , C. D. Malliakas , H. Li , A. J. Freeman , J. T. Kenney , M. G. Kanatzidis , J. Am. Chem. Soc. 2012, 134, 8579. 22578072 10.1021/ja301539s

[58] D. J. Kubicki , D. Prochowicz , E. Salager , A. Rakhmatullin , C. P. Grey , L. Emsley , S. D. Stranks , J. Am. Chem. Soc. 2020, 142, 7813. 32242661 10.1021/jacs.0c00647 PMC7311059

[59] E. L. da Silva , J. M. Skelton , S. C. Parker , A. Walsh , Phys. Rev. B 2015, 91, 144107.

[60] I. Borriello , G. Cantele , D. Ninno , Phys. Rev. B 2008, 77, 235214.

[61] L.‐y. Huang , W. R. L. Lambrecht , Phys. Rev. B 2013, 88, 165203.

[62] A. Stroppa , D. Di Sante , P. Barone , M. Bokdam , G. Kresse , C. Franchini , M.‐H. Whangbo , S. Picozzi , Nat. Commun. 2014, 5, 5900. 25533044 10.1038/ncomms6900

[63] F. Giustino , H. J. Snaith , ACS Energy Lett. 2016, 1, 1233.

[64] W. Shockley , H. J. Queisser , J. Appl. Phys. 1961, 32, 510.

[65] R. S. Drago , J. Phys. Chem. 1958, 62, 353.

[66] R. Prasanna , A. Gold‐Parker , T. Leijtens , B. Conings , A. Babayigit , H.‐G. Boyen , M. F. Toney , M. D. McGehee , J. Am. Chem. Soc. 2017, 139, 11117. 28704048 10.1021/jacs.7b04981

[67] F. Hao , C. C. Stoumpos , D. H. Cao , R. P. H. Chang , M. G. Kanatzidis , Nat. Photonics 2014, 8, 489.

[68] N. K. Noel , S. D. Stranks , A. Abate , C. Wehrenfennig , S. Guarnera , A.‐A. Haghighirad , A. Sadhanala , G. E. Eperon , S. K. Pathak , M. B. Johnston , A. Petrozza , L. M. Herz , H. J. Snaith , Energy Environ. Sci. 2014, 7, 3061.

[69] Z. Xiao , Z. Song , Y. Yan , Adv. Mater. 2019, 31, 1803792. 10.1002/adma.201803792 30680809

[70] G. Xie , L. Xu , L. Sun , Y. Xiong , P. Wu , B. Hu , J. Mater. Chem. A 2019, 7, 5779.

[71] Z. Xiao , Y. Yan , Adv. Energy Mater. 2017, 7, 1701136.

[72] J. Euvrard , Y. Yan , D. B. Mitzi , Nat. Rev. Mater. 2021, 6, 531.

[73] P. Xu , S. Chen , H.‐J. Xiang , X.‐G. Gong , S.‐H. Wei , Chem. Mater. 2014, 26, 6068.

[74] T. Shi , H.‐S. Zhang , W. Meng , Q. Teng , M. Liu , X. Yang , Y. Yan , H.‐L. Yip , Y.‐J. Zhao , J. Mater. Chem. A 2017, 5, 15124.

[75] M. H. Kumar , S. Dharani , W. L. Leong , P. P. Boix , R. R. Prabhakar , T. Baikie , C. Shi , H. Ding , R. Ramesh , M. Asta , M. Graetzel , S. G. Mhaisalkar , N. Mathews , Adv. Mater. 2014, 26, 7122. 25212785 10.1002/adma.201401991

[76] Z. Xiao , W. Meng , J. Wang , D. B. Mitzi , Y. Yan , Mater. Horiz. 2017, 4, 206.

[77] D. Meggiolaro , D. Ricciarelli , A. A. Alasmari , F. A. S. Alasmary , F. De Angelis , J. Phys. Chem. Lett. 2020, 11, 3546. 32298590 10.1021/acs.jpclett.0c00725

[78] W. Ke , C. C. Stoumpos , I. Spanopoulos , L. Mao , M. Chen , M. R. Wasielewski , M. G. Kanatzidis , J. Am. Chem. Soc. 2017, 139, 14800. 28953381 10.1021/jacs.7b09018

[79] W. Ke , C. C. Stoumpos , M. Zhu , L. Mao , I. Spanopoulos , J. Liu , O. Y. Kontsevoi , M. Chen , D. Sarma , Y. Zhang , M. R. Wasielewski , M. G. Kanatzidis , Sci. Adv. 2017, 3, e1701293. 28875173 10.1126/sciadv.1701293 PMC5576879

[80] T. Leijtens , R. Prasanna , A. Gold‐Parker , M. F. Toney , M. D. McGehee , ACS Energy Lett. 2017, 2, 2159.

[81] C. Freysoldt , B. Grabowski , T. Hickel , J. Neugebauer , G. Kresse , A. Janotti , C. G. Van de Walle , Rev. Mod. Phys. 2014, 86, 253.

[82] M. A. Haque , L. H. Hernandez , B. Davaasuren , D. R. Villalva , J. Troughton , D. Baran , Adv. Energy Sustainability Res. 2020, 1, 2000033.

[83] D. B. Mitzi , C. A. Feild , Z. Schlesinger , R. B. Laibowitz , J. Solid State Chem. 1995, 114, 159.

[84] Y. Takahashi , H. Hasegawa , Y. Takahashi , T. Inabe , J. Solid State Chem. 2013, 205, 39.

[85] S. Gupta , T. Bendikov , G. Hodes , D. Cahen , ACS Energy Lett. 2016, 1, 1028.

[86] S. Gupta , D. Cahen , G. Hodes , J. Phys. Chem. C 2018, 122, 13926.

[87] W. Liao , D. Zhao , Y. Yu , C. R. Grice , C. Wang , A. J. Cimaroli , P. Schulz , W. Meng , K. Zhu , R.‐G. Xiong , Y. Yan , Adv. Mater. 2016, 28, 9333. 27571446 10.1002/adma.201602992

[88] R. L. Milot , M. T. Klug , C. L. Davies , Z. Wang , H. Kraus , H. J. Snaith , M. B. Johnston , L. M. Herz , Adv. Mater. 2018, 30, 1804506. 10.1002/adma.201804506 30222220

[89] K. J. Savill , A. M. Ulatowski , M. D. Farrar , M. B. Johnston , H. J. Snaith , L. M. Herz , Adv. Funct. Mater. 2020, 30, 2005594.

[90] S. Zhou , S. Zhu , J. Guan , R. Wang , W. Zheng , P. Gao , X. Lu , J. Phys. Chem. Lett. 2021, 12, 10996. 34739250 10.1021/acs.jpclett.1c03170

[91] B. Lee , C. C. Stoumpos , N. Zhou , F. Hao , C. Malliakas , C.‐Y. Yeh , T. J. Marks , M. G. Kanatzidis , R. P. H. Chang , J. Am. Chem. Soc. 2014, 136, 15379. 25299304 10.1021/ja508464w

[92] A. Liu , H. Zhu , Y. Reo , M.‐G. Kim , H. Y. Chu , J. H. Lim , H.‐J. Kim , W. Ning , S. Bai , Y.‐Y. Noh , Cell Rep. Phys. Sci. 2022, 3, 100812.

[93] B. Saparov , J.‐P. Sun , W. Meng , Z. Xiao , H.‐S. Duan , O. Gunawan , D. Shin , I. G. Hill , Y. Yan , D. B. Mitzi , Chem. Mater. 2016, 28, 2315.

[94] A. Wang , X. Yan , M. Zhang , S. Sun , M. Yang , W. Shen , X. Pan , P. Wang , Z. Deng , Chem. Mater. 2016, 28, 8132.

[95] H. Zhu , A. Liu , K. I. Shim , H. Jung , T. Zou , Y. Reo , H. Kim , J. W. Han , Y. Chen , H. Y. Chu , J. H. Lim , H.‐J. Kim , S. Bai , Y.‐Y. Noh , Nat. Commun. 2022, 13, 1741. 35365628 10.1038/s41467-022-29434-x PMC8975846

[96] S.‐N. Hsu , W. Zhao , Y. Gao , Akriti , M. Segovia , X. Xu , B. W. Boudouris , L. Dou , Nano Lett. 2021, 21, 7839. 34469174 10.1021/acs.nanolett.1c02890

[97] S. Shao , W. Talsma , M. Pitaro , J. Dong , S. Kahmann , A. J. Rommens , G. Portale , M. A. Loi , Adv. Funct. Mater. 2021, 31, 2008478.

[98] R. Begum , M. R. Parida , A. L. Abdelhady , B. Murali , N. M. Alyami , G. H. Ahmed , M. N. Hedhili , O. M. Bakr , O. F. Mohammed , J. Am. Chem. Soc. 2017, 139, 731. 27977176 10.1021/jacs.6b09575

[99] W. Liu , Q. Lin , H. Li , K. Wu , I. Robel , J. M. Pietryga , V. I. Klimov , J. Am. Chem. Soc. 2016, 138, 14954. 27756131 10.1021/jacs.6b08085

[100] M. Saliba , T. Matsui , K. Domanski , J.‐Y. Seo , A. Ummadisingu , S. M. Zakeeruddin , J.‐P. Correa‐Baena , W. R. Tress , A. Abate , A. Hagfeldt , M. Grätzel , Science 2016, 354, 206. 27708053 10.1126/science.aah5557

[101] W. Zhao , Z. Yao , F. Yu , D. Yang , S. Liu , Adv. Sci. 2018, 5, 1700131. 10.1002/advs.201700131 PMC5827644 29610718

[102] L. Hou , Y. Zhu , J. Zhu , Y. Gong , C. Li , J. Mater. Chem. C 2020, 8, 8502.

[103] A. R. Bowman , M. T. Klug , T. A. S. Doherty , M. D. Farrar , S. P. Senanayak , B. Wenger , G. Divitini , E. P. Booker , Z. Andaji‐Garmaroudi , S. Macpherson , E. Ruggeri , H. Sirringhaus , H. J. Snaith , S. D. Stranks , ACS Energy Lett. 2019, 4, 2301. 31544151 10.1021/acsenergylett.9b01446 PMC6748266

[104] Y. Takahashi , R. Obara , K. Nakagawa , M. Nakano , J.‐y. Tokita , T. Inabe , Chem. Mater. 2007, 19, 6312.

[105] R. Zhang , X. Mao , P. Cheng , Y. Yang , S. Yang , T. Wumaier , W. Deng , K. Han , J. Energy Chem. 2019, 36, 1.

[106] Y. Reo , H. Zhu , J.‐Y. Go , K. I. Shim , A. Liu , T. Zou , H. Jung , H. Kim , J. Hong , J. W. Han , Y.‐Y. Noh , Chem. Mater. 2021, 33, 2498.

[107] J.‐Y. Go , H. Zhu , Y. Reo , H. Kim , A. Liu , Y.‐Y. Noh , ACS Appl. Mater. Interfaces 2022, 14, 9363. 35147020 10.1021/acsami.1c19368

[108] P. J. Smith , Chemistry of Tin, Springer, New York 2012.

[109] F. Hao , C. C. Stoumpos , P. Guo , N. Zhou , T. J. Marks , R. P. H. Chang , M. G. Kanatzidis , J. Am. Chem. Soc. 2015, 137, 11445. 26313318 10.1021/jacs.5b06658

[110] M. I. Saidaminov , I. Spanopoulos , J. Abed , W. Ke , J. Wicks , M. G. Kanatzidis , E. H. Sargent , ACS Energy Lett. 2020, 5, 1153.

[111] J. Pascual , G. Nasti , M. H. Aldamasy , J. A. Smith , M. Flatken , N. Phung , D. Di Girolamo , S.‐H. Turren‐Cruz , M. Li , A. Dallmann , R. Avolio , A. Abate , Mater. Adv. 2020, 1, 1066.

[112] Y. Zhou , I. Poli , D. Meggiolaro , F. De Angelis , A. Petrozza , Nat. Rev. Mater. 2021, 6, 986.

[113] D. Di Girolamo , J. Pascual , M. H. Aldamasy , Z. Iqbal , G. Li , E. Radicchi , M. Li , S.‐H. Turren‐Cruz , G. Nasti , A. Dallmann , F. De Angelis , A. Abate , ACS Energy Lett. 2021, 6, 959.

[114] J. Cao , F. Yan , Energy Environ. Sci. 2021, 14, 1286.

[115] T. Wang , F. Yan , Chem. ‐ Asian J. 2020, 15, 1524. 32212294 10.1002/asia.202000160

[116] I. Chung , B. Lee , J. He , R. P. H. Chang , M. G. Kanatzidis , Nature 2012, 485, 486. 22622574 10.1038/nature11067

[117] T. M. Koh , T. Krishnamoorthy , N. Yantara , C. Shi , W. L. Leong , P. P. Boix , A. C. Grimsdale , S. G. Mhaisalkar , N. Mathews , J. Mater. Chem. A 2015, 3, 14996.

[118] L. Ma , F. Hao , C. C. Stoumpos , B. T. Phelan , M. R. Wasielewski , M. G. Kanatzidis , J. Am. Chem. Soc. 2016, 138, 14750. 27750426 10.1021/jacs.6b09257

[119] K. P. Marshall , M. Walker , R. I. Walton , R. A. Hatton , Nat. Energy 2016, 1, 16178.

[120] T.‐B. Song , T. Yokoyama , S. Aramaki , M. G. Kanatzidis , ACS Energy Lett. 2017, 2, 897.

[121] F. Gu , S. Ye , Z. Zhao , H. Rao , Z. Liu , Z. Bian , C. Huang , Sol. RRL 2018, 2, 1800136.

[122] M. E. Kayesh , T. H. Chowdhury , K. Matsuishi , R. Kaneko , S. Kazaoui , J.‐J. Lee , T. Noda , A. Islam , ACS Energy Lett. 2018, 3, 1584.

[123] F. Li , C. Zhang , J.‐H. Huang , H. Fan , H. Wang , P. Wang , C. Zhan , C.‐M. Liu , X. Li , L.‐M. Yang , Y. Song , K.‐J. Jiang , Angew. Chem., Int. Ed. 2019, 58, 6688. 10.1002/anie.201902418 30884017

[124] T.‐B. Song , T. Yokoyama , C. C. Stoumpos , J. Logsdon , D. H. Cao , M. R. Wasielewski , S. Aramaki , M. G. Kanatzidis , J. Am. Chem. Soc. 2017, 139, 836. 27977193 10.1021/jacs.6b10734

[125] J. Cao , Q. Tai , P. You , G. Tang , T. Wang , N. Wang , F. Yan , J. Mater. Chem. A 2019, 7, 26580.

[126] W. Li , J. Li , J. Li , J. Fan , Y. Mai , L. Wang , J. Mater. Chem. A 2016, 4, 17104.

[127] Q. Tai , X. Guo , G. Tang , P. You , T.‐W. Ng , D. Shen , J. Cao , C.‐K. Liu , N. Wang , Y. Zhu , C.‐S. Lee , F. Yan , Angew. Chem., Int. Ed. 2019, 58, 806. 10.1002/anie.201811539 30499609

[128] R. Lin , K. Xiao , Z. Qin , Q. Han , C. Zhang , M. Wei , M. I. Saidaminov , Y. Gao , J. Xu , M. Xiao , A. Li , J. Zhu , E. H. Sargent , H. Tan , Nat. Energy 2019, 4, 864.

[129] J. Pascual , M. Flatken , R. Félix , G. Li , S.‐H. Turren‐Cruz , M. H. Aldamasy , C. Hartmann , M. Li , D. Di Girolamo , G. Nasti , E. Hüsam , R. G. Wilks , A. Dallmann , M. Bär , A. Hoell , A. Abate , Angew. Chem., Int. Ed. 2021, 60, 21583. 10.1002/anie.202107599 PMC8518082 34228886

[130] Z. Dai , T. Lv , J. Barbaud , W. Tang , T. Wang , L. Qiao , H. Chen , R. Zheng , X. Yang , L. Han , Sci. China Mater. 2021, 64, 2645.

[131] H. Zhu , A. Liu , K. I. Shim , J. Hong , J. W. Han , Y.‐Y. Noh , Adv. Mater. 2020, 32, 2002717. 10.1002/adma.202002717 32584475

[132] T. Nakamura , S. Yakumaru , M. A. Truong , K. Kim , J. Liu , S. Hu , K. Otsuka , R. Hashimoto , R. Murdey , T. Sasamori , H. D. Kim , H. Ohkita , T. Handa , Y. Kanemitsu , A. Wakamiya , Nat. Commun. 2020, 11, 3008. 32546736 10.1038/s41467-020-16726-3 PMC7297727

[133] D. Li , H.‐C. Cheng , Y. Wang , Z. Zhao , G. Wang , H. Wu , Q. He , Y. Huang , X. Duan , Adv. Mater. 2017, 29, 1601959. 10.1002/adma.201601959 27859707

[134] F. Li , C. Ma , H. Wang , W. Hu , W. Yu , A. D. Sheikh , T. Wu , Nat. Commun. 2015, 6, 8238. 26345730 10.1038/ncomms9238 PMC4569843

[135] T. Matsushima , S. Terakawa , M. R. Leyden , T. Fujihara , C. Qin , C. Adachi , J. Appl. Phys. 2019, 125, 235501.

[136] L. J. Sutherland , H. C. Weerasinghe , G. P. Simon , Adv. Energy Mater. 2021, 11, 2101383.

[137] Y. He , I. Abdellaoui , M. Abdel‐Shakour , T. H. Chowdhury , M. A. Kamarudin , A. F. Nogueira , Q. Shen , S. Hayase , A. Islam , T. Sakurai , Jpn. J. Appl. Phys. 2021, 60, SBBF13.

[138] X. Zhang , S. Wang , W. Zhu , Z. Cao , A. Wang , F. Hao , Adv. Funct. Mater. 2022, 32, 2108832.

[139] T. Wu , X. Liu , X. Luo , X. Lin , D. Cui , Y. Wang , H. Segawa , Y. Zhang , L. Han , Joule 2021, 5, 863.

[140] Z. Shi , A. H. Jayatissa , Materials 2018, 11, 729. 29734667 10.3390/ma11050729 PMC5978106

[141] Z. Song , S. C. Watthage , A. B. Phillips , M. J. Heben , J. Photonics Energy 2016, 6, 022001.

[142] Z. Zhu , C. C. Chueh , N. Li , C. Mao , A. K. Y. Jen , Adv. Mater. 2018, 30, 1703800. 10.1002/adma.201703800 29250846

[143] H. Dixit , B. Boro , S. Ghosh , M. Paul , A. Kumar , T. Singh , Phys. Status Solidi RRL 2022, 219, 2100823.

[144] D. Huang , T. Goh , J. Kong , Y. Zheng , S. Zhao , Z. Xu , A. D. Taylor , Nanoscale 2017, 9, 4236. 28291270 10.1039/c6nr08375g

[145] H. D. Kim , Y. Miyamoto , H. Kubota , T. Yamanari , H. Ohkita , Chem. Lett. 2017, 46, 253.

[146] T. Handa , T. Yamada , H. Kubota , S. Ise , Y. Miyamoto , Y. Kanemitsu , J. Phys. Chem. C 2017, 121, 16158.

[147] F. Li , C. Zhang , J. H. Huang , H. Fan , H. Wang , P. Wang , C. Zhan , C. M. Liu , X. Li , L. M. Yang , Angew. Chem., Int. Ed. 2019, 58, 6688. 10.1002/anie.201902418 30884017

[148] T. Yokoyama , D. H. Cao , C. C. Stoumpos , T.‐B. Song , Y. Sato , S. Aramaki , M. G. Kanatzidis , J. Phys. Chem. Lett. 2016, 7, 776. 26877089 10.1021/acs.jpclett.6b00118

[149] P. Wang , F. Li , K.‐J. Jiang , Y. Zhang , H. Fan , Y. Zhang , Y. Miao , J.‐H. Huang , C. Gao , X. Zhou , F. Wang , L.‐M. Yang , C. Zhan , Y. Song , Adv. Sci. 2020, 7, 1903047. 10.1002/advs.201903047 PMC7201265 32382478

[150] W.‐F. Yang , J.‐J. Cao , C. Dong , M. Li , Q.‐S. Tian , Z.‐K. Wang , L.‐S. Liao , Appl. Phys. Lett. 2021, 118, 023501.

[151] S. Shahbazi , M.‐Y. Li , A. Fathi , E. W.‐G. Diau , ACS Energy Lett. 2020, 5, 2508.

[152] Y. Dang , Y. Zhou , X. Liu , D. Ju , S. Xia , H. Xia , X. Tao , Angew. Chem., Int. Ed. 2016, 55, 3447. 10.1002/anie.201511792 26889919

[153] J. You , M. Wang , C. Xu , Y. Yao , X. Zhao , D. Liu , J. Dong , P. Guo , G. Xu , C. Luo , Y. Zhong , Q. Song , Sustainable Energy Fuels 2021, 5, 2660.

[154] F. Li , H. Fan , J. Zhang , J.‐H. Huang , P. Wang , C. Gao , L.‐M. Yang , Z. Zhu , A. K.‐Y. Jen , Y. Song , K.‐J. Jiang , Sol. RRL 2019, 3, 1900285.

[155] C. Wang , F. Gu , Z. Zhao , H. Rao , Y. Qiu , Z. Cai , G. Zhan , X. Li , B. Sun , X. Yu , B. Zhao , Z. Liu , Z. Bian , C. Huang , Adv. Mater. 2020, 32, 1907623. 10.1002/adma.201907623 32583926

[156] T. Wang , Q. Tai , X. Guo , J. Cao , C.‐K. Liu , N. Wang , D. Shen , Y. Zhu , C.‐S. Lee , F. Yan , ACS Energy Lett. 2020, 5, 1741.

[157] Z. Zhu , X. Jiang , D. Yu , N. Yu , Z. Ning , Q. Mi , ACS Energy Lett. 2022, 7, 2079.

[158] E. Jokar , C.‐H. Chien , A. Fathi , M. Rameez , Y.‐H. Chang , E. W.‐G. Diau , Energy Environ. Sci. 2018, 11, 2353.

[159] T. Wu , X. Liu , X. He , Y. Wang , X. Meng , T. Noda , X. Yang , L. Han , Sci. China: Chem. 2020, 63, 107.

[160] X. Meng , J. Lin , X. Liu , X. He , Y. Wang , T. Noda , T. Wu , X. Yang , L. Han , Adv. Mater. 2019, 31, 1903721. 10.1002/adma.201903721 31495977

[161] T. H. Chowdhury , R. Kaneko , T. Kaneko , K. Sodeyama , J.‐J. Lee , A. Islam , Chem. Eng. J. 2022, 431, 133745.

[162] X. Meng , Y. Wang , J. Lin , X. Liu , X. He , J. Barbaud , T. Wu , T. Noda , X. Yang , L. Han , Joule 2020, 4, 902.

[163] Z. Dai , W. Tang , T. Wang , T. Lv , X. Luo , D. Cui , R. Sun , L. Qiao , H. Chen , R. Zheng , X. Yang , L. Han , Sci. China Mater. 2021, 64, 1849.

[164] X. Jiang , H. Li , Q. Zhou , Q. Wei , M. Wei , L. Jiang , Z. Wang , Z. Peng , F. Wang , Z. Zang , K. Xu , Y. Hou , S. Teale , W. Zhou , R. Si , X. Gao , E. H. Sargent , Z. Ning , J. Am. Chem. Soc. 2021, 143, 10970. 34196528 10.1021/jacs.1c03032

[165] K. Cao , Y. Cheng , J. Chen , Y. Huang , M. Ge , J. Qian , L. Liu , J. Feng , S. Chen , W. Huang , ACS Appl. Mater. Interfaces 2020, 12, 41454. 32829633 10.1021/acsami.0c11253

[166] G. Liu , C. Liu , Z. Lin , J. Yang , Z. Huang , L. Tan , Y. Chen , ACS Appl. Mater. Interfaces 2020, 12, 14049. 32129060 10.1021/acsami.0c01311

[167] T. H. Chowdhury , M. E. Kayesh , J.‐J. Lee , Y. Matsushita , S. Kazaoui , A. Islam , Sol. RRL 2019, 3, 1900245.

[168] M. A. Kamarudin , D. Hirotani , Z. Wang , K. Hamada , K. Nishimura , Q. Shen , T. Toyoda , S. Iikubo , T. Minemoto , K. Yoshino , S. Hayase , J. Phys. Chem. Lett. 2019, 10, 5277. 31423786 10.1021/acs.jpclett.9b02024

[169] X. Liu , T. Wu , J.‐Y. Chen , X. Meng , X. He , T. Noda , H. Chen , X. Yang , H. Segawa , Y. Wang , L. Han , Energy Environ. Sci. 2020, 13, 2896.

[170] J.‐Y. Seo , T. Matsui , J. Luo , J.‐P. Correa‐Baena , F. Giordano , M. Saliba , K. Schenk , A. Ummadisingu , K. Domanski , M. Hadadian , A. Hagfeldt , S. M. Zakeeruddin , U. Steiner , M. Grätzel , A. Abate , Adv. Energy Mater. 2016, 6, 1600767.

[171] Z. Lin , Y. Su , R. Dai , G. Liu , J. Yang , W. Sheng , Y. Zhong , L. Tan , Y. Chen , ACS Appl. Mater. Interfaces 2021, 13, 15420. 33759500 10.1021/acsami.1c01408

[172] R. Xu , H. Dong , P. Li , X. Cao , H. Li , J. Li , Z. Wu , ACS Appl. Mater. Interfaces 2021, 13, 33218. 34228914 10.1021/acsami.1c05097

[173] G. Li , Z. Su , M. Li , F. Yang , M. H. Aldamasy , J. Pascual , F. Yang , H. Liu , W. Zuo , D. Di Girolamo , Z. Iqbal , G. Nasti , A. Dallmann , X. Gao , Z. Wang , M. Saliba , A. Abate , Adv. Energy Mater. 2021, 11, 2101539.

[174] A. M. Boehm , T. Liu , S. M. Park , A. Abtahi , K. R. Graham , ACS Appl. Mater. Interfaces 2020, 12, 5209. 31887000 10.1021/acsami.9b17535

[175] M. Abdel‐Shakour , T. H. Chowdhury , K. Matsuishi , M. A. Karim , Y. He , Y. Moritomo , A. Islam , ACS Appl. Energy Mater. 2021, 4, 12515.

[176] M. Chen , Q. Dong , C. Xiao , X. Zheng , Z. Dai , Y. Shi , J. M. Luther , N. P. Padture , ACS Energy Lett. 2022, 7, 2256.

[177] B. P. Nguyen , H. R. Jung , K. Y. Ryu , K. Kim , W. Jo , J. Phys. Chem. C 2019, 123, 30833.

[178] Z. Zhao , F. Gu , Y. Li , W. Sun , S. Ye , H. Rao , Z. Liu , Z. Bian , C. Huang , Adv. Sci. 2017, 4, 1700204. 10.1002/advs.201700204 PMC5700647 29201617

[179] X. Liu , K. Yan , D. Tan , X. Liang , H. Zhang , W. Huang , ACS Energy Lett. 2018, 3, 2701.

[180] Z. Wan , S. Ren , H. Lai , Y. Jiang , X. Wu , J. Luo , Y. Wang , R. He , Q. Chen , X. Hao , Y. Wang , L. Wu , I. Constantinou , W.‐H. Zhang , J. Zhang , D. Zhao , Adv. Mater. Interfaces 2021, 8, 2100135.

[181] Z. Zhang , A. Kumar Baranwal , S. Razey Sahamir , G. Kapil , Y. Sanehira , M. Chen , K. Nishimura , C. Ding , D. Liu , H. Li , Y. Li , M. Akmal Kamarudin , Q. Shen , T. S. Ripolles , J. Bisquert , S. Hayase , Sol. RRL 2021, 5, 2100633.

[182] K. Nishimura , M. A. Kamarudin , D. Hirotani , K. Hamada , Q. Shen , S. Iikubo , T. Minemoto , K. Yoshino , S. Hayase , Nano Energy 2020, 74, 104858.

[183] Z. Zhang , L. Wang , A. Kumar Baranwal , S. Razey Sahamir , G. Kapil , Y. Sanehira , M. Akmal Kamarudin , K. Nishimura , C. Ding , D. Liu , Y. Li , H. Li , M. Chen , Q. Shen , T. S. Ripolles , J. Bisquert , S. Hayase , J. Energy Chem. 2022, 71, 604.

[184] S. Weber , T. Rath , B. Kunert , R. Resel , T. Dimopoulos , G. Trimmel , Monatsh. Chem. 2019, 150, 1921.

[185] M. Chen , M. A. Kamarudin , A. K. Baranwal , G. Kapil , T. S. Ripolles , K. Nishimura , D. Hirotani , S. R. Sahamir , Z. Zhang , C. Ding , Y. Sanehira , J. Bisquert , Q. Shen , S. Hayase , ACS Appl. Energy Mater. 2021, 4, 5615.

[186] M. Chen , G. Kapil , L. Wang , S. Razey Sahamir , A. K. Baranwal , K. Nishimura , Y. Sanehira , Z. Zhang , M. Akmal Kamarudin , Q. Shen , S. Hayase , Chem. Eng. J. 2022, 436, 135196.

[187] M. E. Kayesh , K. Matsuishi , R. Kaneko , S. Kazaoui , J.‐J. Lee , T. Noda , A. Islam , ACS Energy Lett. 2018, 4, 278.

[188] J. Liu , M. Ozaki , S. Yakumaru , T. Handa , R. Nishikubo , Y. Kanemitsu , A. Saeki , Y. Murata , R. Murdey , A. Wakamiya , Angew. Chem., Int. Ed. 2018, 57, 13221. 10.1002/anie.201808385 30110137

[189] M. Konstantakou , T. Stergiopoulos , J. Mater. Chem. A 2017, 5, 11518.

[190] Z. Chen , C. Yu , K. Shum , J. J. Wang , W. Pfenninger , N. Vockic , J. Midgley , J. T. Kenney , J. Lumin. 2012, 132, 345.

[191] K. P. Marshall , R. I. Walton , R. A. Hatton , J. Mater. Chem. A 2015, 3, 11631.

[192] K. Marshall , M. Walker , R. Walton , R. Hatton , Nat. Energy 2016, 1, 16178.

[193] H. Ban , T. Zhang , X. Gong , Q. Sun , X.‐L. Zhang , N. Pootrakulchote , Y. Shen , M. Wang , Sol. RRL 2021, 5, 2100069.

[194] T. Ye , K. Wang , Y. Hou , D. Yang , N. Smith , B. Magill , J. Yoon , R. R. H. H. Mudiyanselage , G. A. Khodaparast , K. Wang , S. Priya , J. Am. Chem. Soc. 2021, 143, 4319. 33705120 10.1021/jacs.0c13069

[195] T. Ye , X. Wang , K. Wang , S. Ma , D. Yang , Y. Hou , J. Yoon , K. Wang , S. Priya , ACS Energy Lett. 2021, 6, 1480.

[196] M.‐G. Ju , J. Dai , L. Ma , X. C. Zeng , J. Am. Chem. Soc. 2017, 139, 8038. 28537073 10.1021/jacs.7b04219

[197] M. Chen , M.‐G. Ju , H. F. Garces , A. D. Carl , L. K. Ono , Z. Hawash , Y. Zhang , T. Shen , Y. Qi , R. L. Grimm , D. Pacifici , X. C. Zeng , Y. Zhou , N. P. Padture , Nat. Commun. 2019, 10, 16. 30604757 10.1038/s41467-018-07951-y PMC6318336

[198] P. Zhu , C. Chen , S. Gu , R. Lin , J. Zhu , Sol. RRL 2018, 2, 1700224.

[199] J. Li , J. Huang , A. Zhao , Y. Li , M. Wei , J. Mater. Chem. C 2020, 8, 8840.

[200] B. Li , H. Di , B. Chang , R. Yin , L. Fu , Y.‐N. Zhang , L. Yin , Adv. Funct. Mater. 2021, 31, 2007447.

[201] L. Ji , D. Liu , Y. Wang , T. Zhang , H. Chen , Y. Li , H. Zheng , Y. Yang , Z. D. Chen , W. Yang , Chem. Eng. J. 2020, 402, 125133.

[202] S. Shao , J. Liu , G. Portale , H.‐H. Fang , G. R. Blake , G. H. ten Brink , L. J. A. Koster , M. A. Loi , Adv. Energy Mater. 2018, 8, 1702019.

[203] S. Shao , M. Nijenhuis , J. Dong , S. Kahmann , G. H. ten Brink , G. Portale , M. A. Loi , J. Mater. Chem. A 2021, 9, 10095.

[204] F. Wang , X. Jiang , H. Chen , Y. Shang , H. Liu , J. Wei , W. Zhou , H. He , W. Liu , Z. Ning , Joule 2018, 2, 2732.

[205] M. Chen , Q. Dong , F. T. Eickemeyer , Y. Liu , Z. Dai , A. D. Carl , B. Bahrami , A. H. Chowdhury , R. L. Grimm , Y. Shi , Q. Qiao , S. M. Zakeeruddin , M. Grätzel , N. P. Padture , ACS Energy Lett. 2020, 5, 2223.

[206] X. Jiang , F. Wang , Q. Wei , H. Li , Y. Shang , W. Zhou , C. Wang , P. Cheng , Q. Chen , L. Chen , Z. Ning , Nat. Commun. 2020, 11, 1245. 32144245 10.1038/s41467-020-15078-2 PMC7060347

[207] B.‐B. Yu , Z. Chen , Y. Zhu , Y. Wang , B. Han , G. Chen , X. Zhang , Z. Du , Z. He , Adv. Mater. 2021, 33, 2102055. 10.1002/adma.202102055 34296476

[208] T. Wang , H.‐L. Loi , J. Cao , Z. Qin , Z. Guan , Y. Xu , H. Cheng , M. G. Li , C.‐S. Lee , X. Lu , F. Yan , Adv. Sci. 2022, 9, 2200242. 10.1002/advs.202200242 PMC9218751 35460202

[209] C. Ran , J. Xi , W. Gao , F. Yuan , T. Lei , B. Jiao , X. Hou , Z. Wu , ACS Energy Lett. 2018, 3, 713.

[210] M. Liao , B.‐B. Yu , Z. Jin , W. Chen , Y. Zhu , X. Zhang , W. Yao , T. Duan , I. Djerdj , Z. He , ChemSusChem 2019, 12, 5007. 31468722 10.1002/cssc.201902000

[211] J. Qiu , Y. Xia , Y. Zheng , W. Hui , H. Gu , W. Yuan , H. Yu , L. Chao , T. Niu , Y. Yang , X. Gao , Y. Chen , W. Huang , ACS Energy Lett. 2019, 4, 1513.

[212] M. Li , W.‐W. Zuo , Y.‐G. Yang , M. H. Aldamasy , Q. Wang , S. H. T. Cruz , S.‐L. Feng , M. Saliba , Z.‐K. Wang , A. Abate , ACS Energy Lett. 2020, 5, 1923.

[213] W. Chen , Y. Wu , Y. Yue , J. Liu , W. Zhang , X. Yang , H. Chen , E. Bi , I. Ashraful , M. Grätzel , Science 2015, 350, 944. 26516198 10.1126/science.aad1015

[214] C. Ran , W. Gao , J. Li , J. Xi , L. Li , J. Dai , Y. Yang , X. Gao , H. Dong , B. Jiao , I. Spanopoulos , C. D. Malliakas , X. Hou , M. G. Kanatzidis , Z. Wu , Joule 2019, 3, 3072.

[215] X. Liu , T. Wu , X. Luo , H. Wang , M. Furue , T. Bessho , Y. Zhang , J. Nakazaki , H. Segawa , L. Han , ACS Energy Lett. 2022, 7, 425.

[216] B. Kang , F. Ge , L. Qiu , K. Cho , Adv. Electron. Mater. 2017, 3, 1600240.

[217] D. Li , G. Wang , H.‐C. Cheng , C.‐Y. Chen , H. Wu , Y. Liu , Y. Huang , X. Duan , Nat. Commun. 2016, 7, 11330. 27098114 10.1038/ncomms11330 PMC4844678

[218] J. Zaumseil , H. Sirringhaus , Chem. Rev. 2007, 107, 1296. 17378616 10.1021/cr0501543

[219] R. A. McKee , F. J. Walker , M. F. Chisholm , Phys. Rev. Lett. 1998, 81, 3014.

[220] L. G. Meiners , J. Vac. Sci. Technol. 1981, 19, 373.

[221] M. L. Green , T. W. Sorsch , G. L. Timp , D. A. Muller , B. E. Weir , P. J. Silverman , S. V. Moccio , Y. O. Kim , Microelectron. Eng. 1999, 48, 25.

[222] A. Liu , H. Zhu , H. Sun , Y. Xu , Y.‐Y. Noh , Adv. Mater. 2018, 30, 1706364. 10.1002/adma.201706364 29904984

[223] K. Nomura , H. Ohta , A. Takagi , T. Kamiya , M. Hirano , H. Hosono , Nature 2004, 432, 488. 15565150 10.1038/nature03090

[224] A. Liu , H. Zhu , Y.‐Y. Noh , Mater. Sci. Eng., R 2019, 135, 85.

[225] H. Sirringhaus , Adv. Mater. 2014, 26, 1319. 24443057 10.1002/adma.201304346 PMC4515091

[226] D. Khim , H. Han , K.‐J. Baeg , J. Kim , S.‐W. Kwak , D.‐Y. Kim , Y.‐Y. Noh , Adv. Mater. 2013, 25, 4302. 23580467 10.1002/adma.201205330

[227] H. Sirringhaus , T. Kawase , R. H. Friend , T. Shimoda , M. Inbasekaran , W. Wu , E. P. Woo , Science 2000, 290, 2123. 11118142 10.1126/science.290.5499.2123

[228] G. Giorgi , J.‐I. Fujisawa , H. Segawa , K. Yamashita , J. Phys. Chem. Lett. 2013, 4, 4213. 26296167 10.1021/jz4023865

[229] C. S. Ponseca , T. J. Savenije , M. Abdellah , K. Zheng , A. Yartsev , T. Pascher , T. Harlang , P. Chabera , T. Pullerits , A. Stepanov , J.‐P. Wolf , V. Sundström , J. Am. Chem. Soc. 2014, 136, 5189. 24654882 10.1021/ja412583t

[230] H. Zhu , A. Liu , Y.‐Y. Noh , Nat. Electron. 2020, 3, 662.

[231] H. Zhu , A. Liu , Y.‐Y. Noh , J. Inf. Disp. 2021, 22, 257.

[232] Y. Liu , P.‐A. Chen , Y. Hu , J. Mater. Chem. C 2020, 8, 16691.

[233] C. Zhang , P. Chen , W. Hu , Small 2016, 12, 1252. 26833896 10.1002/smll.201502546

[234] M. Kim , S. U. Ryu , S. A. Park , K. Choi , T. Kim , D. Chung , T. Park , Adv. Funct. Mater. 2020, 30, 1904545.

[235] T. Yang , Q. Wu , F. Dai , K. Huang , H. Xu , C. Liu , C. Chen , S. Hu , X. Liang , X. Liu , Y.‐Y. Noh , C. Liu , Adv. Funct. Mater. 2020, 30, 1903889.

[236] H. Zhu , E.‐S. Shin , A. Liu , D. Ji , Y. Xu , Y.‐Y. Noh , Adv. Funct. Mater. 2020, 30, 1904588.

[237] Y.‐H. Lin , P. Pattanasattayavong , T. D. Anthopoulos , Adv. Mater. 2017, 29, 1702838. 10.1002/adma.201702838 29024040

[238] T. Matsushima , S. Hwang , A. S. D. Sandanayaka , C. Qin , S. Terakawa , T. Fujihara , M. Yahiro , C. Adachi , Adv. Mater. 2016, 28, 10275. 27605061 10.1002/adma.201603126

[239] S. P. Senanayak , M. Abdi‐Jalebi , V. S. Kamboj , R. Carey , R. Shivanna , T. Tian , G. Schweicher , J. Wang , N. Giesbrecht , D. D. Nuzzo , H. E. Beere , P. Docampo , D. A. Ritchie , D. Fairen‐Jimenez , R. H. Friend , H. Sirringhaus , Sci. Adv. 2020, 6, eaaz4948. 32300658 10.1126/sciadv.aaz4948 PMC7148112

[240] C. R. Kagan , D. B. Mitzi , C. D. Dimitrakopoulos , Science 1999, 286, 945. 10542146 10.1126/science.286.5441.945

[241] E. Shi , Y. Gao , B. P. Finkenauer , Akriti , A. H. Coffey , L. Dou , Chem. Soc. Rev. 2018, 47, 6046. 29564440 10.1039/C7CS00886D

[242] W. Zhao , S.‐N. Hsu , B. W. Boudouris , L. Dou , ACS Appl. Electron. Mater. 2021, 3, 5155.

[243] D. B. Mitzi , C. D. Dimitrakopoulos , J. Rosner , D. R. Medeiros , Z. Xu , C. Noyan , Adv. Mater. 2002, 14, 1772.

[244] T. Matsushima , K. Fujita , T. Tsutsui , Jpn. J. Appl. Phys. 2004, 43, L1199.

[245] T. Matsushima , T. Yasuda , K. Fujita , C. Adachi , J. Appl. Phys. 2016, 120, 233301.

[246] T. Matsushima , S. Hwang , S. Terakawa , T. Fujihara , A. S. D. Sandanayaka , C. Qin , C. Adachi , Appl. Phys. Express 2017, 10, 024103.

[247] T. Matsushima , M. R. Leyden , T. Fujihara , C. Qin , A. S. D. Sandanayaka , C. Adachi , Appl. Phys. Lett. 2019, 115, 120601.

[248] T. Matsushima , F. Mathevet , B. Heinrich , S. Terakawa , T. Fujihara , C. Qin , A. S. D. Sandanayaka , J.‐C. Ribierre , C. Adachi , Appl. Phys. Lett. 2016, 109, 253301.

[249] F. Zhang , H. Zhang , L. Zhu , L. Qin , Y. Wang , Y. Hu , Z. Lou , Y. Hou , F. Teng , J. Mater. Chem. C 2019, 7, 4004.

[250] H. Zhu , A. Liu , H. Kim , J. Hong , J.‐Y. Go , Y.‐Y. Noh , Chem. Mater. 2021, 33, 1174.

[251] H. Zhu , A. Liu , T. Zou , H. Jung , S. Heo , Y. Y. Noh , Mater. Today Energy 2021, 21, 100722.

[252] C. Chen , X. Zhang , G. Wu , H. Li , H. Chen , Adv. Opt. Mater. 2017, 5, 1600539.

[253] C. Xie , C.‐K. Liu , H.‐L. Loi , F. Yan , Adv. Funct. Mater. 2020, 30, 1903907.

[254] H. Gu , S.‐C. Chen , Q. Zheng , Adv. Opt. Mater. 2021, 9, 2001637.

[255] X. Huang , Y. Guo , Y. Liu , Chem. Commun. 2021, 57, 11429. 10.1039/d1cc04447h 34642713

[256] Y. Li , Z. Shi , W. Liang , J. Ma , X. Chen , D. Wu , Y. Tian , X. Li , C. Shan , X. Fang , Mater. Horiz. 2021, 8, 1367. 34846447 10.1039/d0mh01567a

[257] X. Li , X. Gao , X. Zhang , X. Shen , M. Lu , J. Wu , Z. Shi , V. L. Colvin , J. Hu , X. Bai , W. W. Yu , Y. Zhang , Adv. Sci. 2021, 8, 2003334. 10.1002/advs.202003334 PMC7887601 33643803

[258] Y. Zhang , Y. Ma , Y. Wang , X. Zhang , C. Zuo , L. Shen , L. Ding , Adv. Mater. 2021, 33, 2006691. 10.1002/adma.202006691 34028107

[259] A. Liang , Y. Gao , R. Asadpour , Z. Wei , B. P. Finkenauer , L. Jin , J. Yang , K. Wang , K. Chen , P. Liao , C. Zhu , L. Huang , B. W. Boudouris , M. A. Alam , L. Dou , J. Am. Chem. Soc. 2021, 143, 15215. 34516736 10.1021/jacs.1c06337

[260] Y. Gao , Z. Wei , P. Yoo , E. Shi , M. Zeller , C. Zhu , P. Liao , L. Dou , J. Am. Chem. Soc. 2019, 141, 15577. 31525969 10.1021/jacs.9b06276

[261] J. Kim , Y.‐S. Shiah , K. Sim , S. Iimura , K. Abe , M. Tsuji , M. Sasase , H. Hosono , Adv. Sci. 2022, 9, 2104993. 10.1002/advs.202104993 PMC8844482 34927379

[262] H. Shen , J. Li , H. Wang , J. Ma , J. Wang , H. Luo , D. Li , J. Phys. Chem. Lett. 2019, 10, 7. 30556387 10.1021/acs.jpclett.8b03381

[263] C.‐K. Liu , Q. Tai , N. Wang , G. Tang , H.‐L. Loi , F. Yan , Adv. Sci. 2019, 6, 1900751. 10.1002/advs.201900751 PMC6724360 31508281

[264] C.‐K. Liu , Q. Tai , N. Wang , G. Tang , Z. Hu , F. Yan , ACS Appl. Mater. Interfaces 2020, 12, 18769. 32212606 10.1021/acsami.0c01202

[265] E. Fortunato , P. Barquinha , R. Martins , Adv. Mater. 2012, 24, 2945. 22573414 10.1002/adma.201103228

[266] F. Hao , C. C. Stoumpos , R. P. H. Chang , M. G. Kanatzidis , J. Am. Chem. Soc. 2014, 136, 8094. 24823301 10.1021/ja5033259

[267] M. T. Klug , R. L. Milot , J. B. Patel , T. Green , H. C. Sansom , M. D. Farrar , A. J. Ramadan , S. Martani , Z. Wang , B. Wenger , J. M. Ball , L. Langshaw , A. Petrozza , M. B. Johnston , L. M. Herz , H. J. Snaith , Energy Environ. Sci. 2020, 13, 1776.

[268] J. Wang , K. Datta , J. Li , M. A. Verheijen , D. Zhang , M. M. Wienk , R. A. J. Janssen , Adv. Energy Mater. 2020, 10, 2000566.

[269] M. Xiao , F. Huang , W. Huang , Y. Dkhissi , Y. Zhu , J. Etheridge , A. Gray‐Weale , U. Bach , Y.‐B. Cheng , L. Spiccia , Angew. Chem., Int. Ed. 2014, 53, 9898. 10.1002/anie.201405334 25047967

[270] F. Zuo , S. T. Williams , P.‐W. Liang , C.‐C. Chueh , C.‐Y. Liao , A. K.‐Y. Jen , Adv. Mater. 2014, 26, 6454. 25123496 10.1002/adma.201401641

[271] Y. S. Rim , H. Chen , B. Zhu , S.‐H. Bae , S. Zhu , P. J. Li , I. C. Wang , Y. Yang , Adv. Mater. Interfaces 2017, 4, 1700020.

[272] S. Park , S. H. Kim , H. H. Choi , B. Kang , K. Cho , Adv. Funct. Mater. 2020, 30, 1904590.

[273] J. Xi , M. A. Loi , ACS Energy Lett. 2021, 6, 1803. 34056109 10.1021/acsenergylett.1c00289 PMC8155387

[274] S. Ullah , J. Wang , P. Yang , L. Liu , J. khan , S.‐E. Yang , T. Xia , H. Guo , Y. Chen , Sol. RRL 2021, 5, 2000830.

[275] Y. Chu , Y. Hu , Z. Xiao , J. Phys. Chem. C 2021, 125, 9688.

[276] J. C.‐R. Ke , D. J. Lewis , A. S. Walton , B. F. Spencer , P. O'Brien , A. G. Thomas , W. R. Flavell , J. Mater. Chem. A 2018, 6, 11205.

[277] F. Zhang , Q. Zhang , X. Liu , Y. Hu , Z. Lou , Y. Hou , F. Teng , ACS Appl. Mater. Interfaces 2021, 13, 24272. 33983724 10.1021/acsami.1c03041

[278] F. Zhang , Q. Zhang , X. Liu , L. Qin , Y. Hu , Z. Lou , Y. Hou , F. Teng , J. Mater. Chem. A 2021, 9, 22842.

[279] C. Qin , F. Zhang , L. Qin , X. Liu , H. Ji , L. Li , Y. Hu , Z. Lou , Y. Hou , F. Teng , Adv. Electron. Mater. 2021, 7, 2100384.

[280] H. Zhu , A. Liu , H. L. Luque , H. Sun , D. Ji , Y.‐Y. Noh , ACS Nano 2019, 13, 3971. 30844243 10.1021/acsnano.8b07567

[281] Z. Wei , K. Wang , W. Zhao , Y. Gao , Q. Hu , K. Chen , L. Dou , Chem. Commun. 2021, 57, 11469. 10.1039/d1cc04679a 34652357

